# Novel d-Annulated Pentacyclic Steroids: Regioselective Synthesis and Biological Evaluation in Breast Cancer Cells

**DOI:** 10.3390/molecules25153499

**Published:** 2020-07-31

**Authors:** Svetlana K. Vorontsova, Anton V. Yadykov, Alexander M. Scherbakov, Mikhail E. Minyaev, Igor V. Zavarzin, Ekaterina I. Mikhaevich, Yulia A. Volkova, Valerii Z. Shirinian

**Affiliations:** 1 N.D. Zelinsky Institute of Organic Chemistry, Russian Academy of Sciences, Leninsky Prosp. 47, 119991 Moscow, Russia; vorontsova_s@mail.ru (S.K.V.); antonyadykov@gmail.com (A.V.Y.); mminyaev@mail.ru (M.E.M.); igorzavarzin@yandex.ru (I.V.Z.); svbegunt@mail.ru (V.Z.S.); 2 N.N. Blokhin National Medical Research Center of Oncology, Kashirskoye Shosse 24, 115522 Moscow, Russia; alex.scherbakov@gmail.com (A.M.S.); k.mihaevich@gmail.com (E.I.M.)

**Keywords:** interrupted Nazarov cyclization, pentacyclic steroids, antiproliferative activity, d-annulated steroids, Lewis acid

## Abstract

The acid-catalyzed cyclization of benzylidenes based on 16-dehydropregnenolone acetate (16-DPA) was studied. It was found that these compounds readily undergo regioselective interrupted Nazarov cyclization with trapping chloride ion and an efficient method of the synthesis of d-annulated pentacyclic steroids based on this reaction was proposed. The structures of the synthesized pentacyclic steroids were determined by NMR and X-ray diffraction. It was found that the reaction affords a single diastereomer, but the latter can crystallize as two conformers depending on the structure. Antiproliferative activity of synthesized compounds was evaluated against two breast cancer cell lines: MCF-7 and MDA-MB-231. All tested compounds showed relatively high antiproliferative activity. The synthetic potential of the protocol developed was illustrated by the gram-scale experiment.

## 1. Introduction

**Scheme 1:**
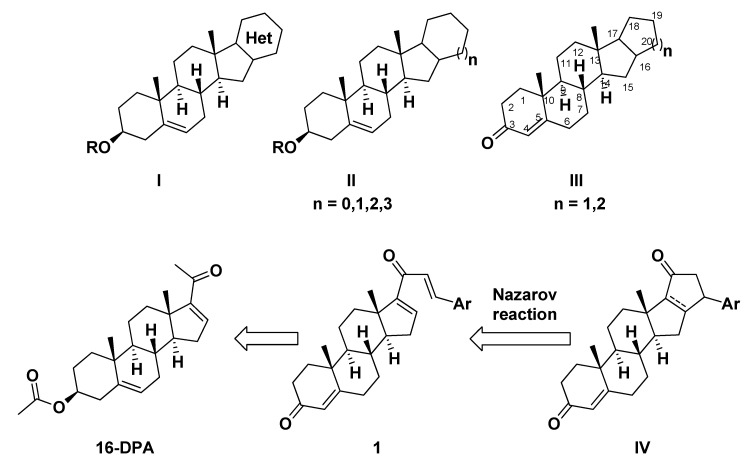
Pentacyclic steroids.

Steroids are an important class of both natural and synthetic products exhibiting various biological activities [1,2,3,4,5,6,7,8,9,10,11]. In the past decades, great efforts were made to accomplish further modification to synthesize structurally new and biologically interesting compounds [12,13,14,15,16,17]. The chemical modification of steroids is among the most efficient and attractive approaches to the design of new biologically active compounds, including pharmaceuticals [1,2,18,19]. An important application of chemical modification of steroid molecules is to synthesize compounds containing an additional fused ring. Steroids bearing an additional fused ring are common skeletons involved in many natural products [20,21,22,23,24] and pharmaceuticals [25,26,27]. It should be noted that some of these compounds exhibit no significant hormonal activity [28,29,30,31]. Both heterocyclic analogs **I** [32,33,34,35,36,37,38,39] and carbocyclic analogs **II** [40,41] of pentacyclic steroids have attracted considerable attention (Scheme 1).

Steroids bearing an additional carbocycle annulated at the 16 and 17 positions of the ring D (**III**) are characterized by unique biological activity [42,43,44,45]. Most of the known D-annulated pentacyclic steroids contain the cyclohexane or cyclopentane moiety as an additional ring. A number of synthetic strategies can be used to construct pentacyclic steroids, including Diels–Alder reactions and intermolecular condensation [40,41,46,47,48,49]. Commercially available 16-dehydropregnenolone acetate (16-DPA), which is a versatile building block for the preparation of various semi-synthetic steroidal drugs [50,51], is commonly utilized in the synthesis of D-annulated steroids. However, despite the availability of 16-DPA, most of the known methods for the construction of an additional carbocycle based on 16-DPA cannot be applied on a preparative scale and often suffer from drawbacks such as low yields, the lack of atom-economy or step efficiency, a narrow field of application, etc.

In a continuation of our research on the synthesis of semi-synthetic steroid derivatives and evaluation of their antitumor activity [36,37,52,53], we focused on the development of a convenient method for the synthesis of D-annulated pentacyclic steroid of the progesterone series **IV** bearing the cyclopentanone moiety as an additional ring. The Nazarov cyclization reaction represents one of the most effective methods for the construction of five-membered carbocyclic rings, and it has been applied in the total synthesis of many useful natural products. Due to high stereoselectivity, high yields, and readily available starting compounds, this reaction is widely used in organic synthesis for the preparation of diverse biologically active substances [54,55,56,57,58,59,60,61,62,63,64,65,66,67,68,69,70,71,72]. However, to the best of our knowledge, this reaction was not applied for the annulation of an additional cyclopentanone ring onto the steroid skeleton. Another important issue, which prompted us to perform this study, is that the starting benzylidenes **1** are readily available by the method proposed in our recent work [52,53]. Besides, the synthesized benzylidenes exhibited relatively high antiproliferative activity. Since the structure of the starting dienone does not undergo significant changes in the course of Nazarov cyclization, this modification would be expected to improve useful properties.

**Scheme 2:**
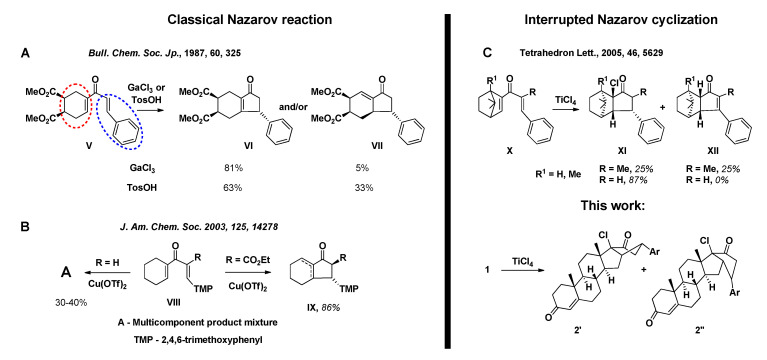
Nazarov reaction of dienones in the presence of various catalysts (**A**—GaCl_3_ or TosOH, **B**—Cu(OTf)_2_, **C**—TiCl_4_)

Despite the absence of data on the synthesis of pentacyclic steroids by the Nazarov reaction, several examples of the application of this transformation both in the synthesis and modification of steroids were reported [73,74,75,76,77]. However, since the pathway of the Nazarov reaction and the yields of the target products strongly depend on the nature of substituents and the catalyst, we analyzed the cyclization of compounds structurally similar to benzylidenes **1**, such as dienones, in which one vinyl moiety is involved in the carbocycle, while another vinyl moiety contains an aryl group at the *β*-position (compound **V**, Scheme 2A). Literature data analysis showed that there are only a few examples of cyclization of structurally related dienones (Scheme 2).

The cyclization of dienones in the presence of various Lewis acids (SnCl_4_, TiCl_4_, AlCl_3_, GaCl_3_, BF_3_*Et_2_O) and TsOH was studied [78]. It was found that despite the absence of the second α-substituent, the cyclization affords a classical Nazarov reaction product (Scheme 2A). The best yields were achieved using gallium chloride, which gave the minimum amount of the second isomer (5%). The use of p-toluenesulfonic acid as the catalyst leads to an increase in the amount of the minor isomer. It is worth noting that, despite the absence of the second α-substituent, the formation of interrupted Nazarov cyclization products was not observed. Dienone **VIII** (R = CO_2_Et) also underwent classical Nazarov cyclization in the presence of copper (II) triflate (Scheme 2B) [79]. This reaction also produced two isomers. Under similar conditions, the cyclization of α-unsubstituted substrate **VIII** (R = H) affords a difficult-to-separate mixture. Interesting results were obtained in the study of the cyclization of camphor- and nopinone-derived dienones **X** [80]. The interrupted Nazarov cyclization via nucleophilic halide trapping was found to occur in the presence of halogen-contained Lewis acids (TiCl_4_). The nucleophilic halide trapping was observed not only in the presence of titanium chloride but also in the presence of bulky bromide and iodide anions (TiBr_4_ and TiI_4_); the exception was the fluoride anion. The interrupted Nazarov cyclization proceeds with α-unsubstituted compounds. The introduction of the methyl group at this position facilitates the competitive classical Nazarov reaction, resulting in the formation of a mixture of products in moderate yields. It is worth noting that this is one of a few studies on the Nazarov reaction, in which halide trapping in the presence of Lewis acids took place [81,82].

Such transformations are also known for other cationic rearrangements [83,84,85,86,87]. Therefore, the analysis of the literature data shows that the pathway of the cyclization of such dienones strongly depends on both their structure and the nature of the catalyst. Since camphor-derived dienones **X** (R = Me) are structurally most similar to benzylidenes **1**, we decided to study the cyclization of these steroids under similar conditions.

## 2. Results and Discussion

### 2.1. Synthesis

**Scheme 3:**
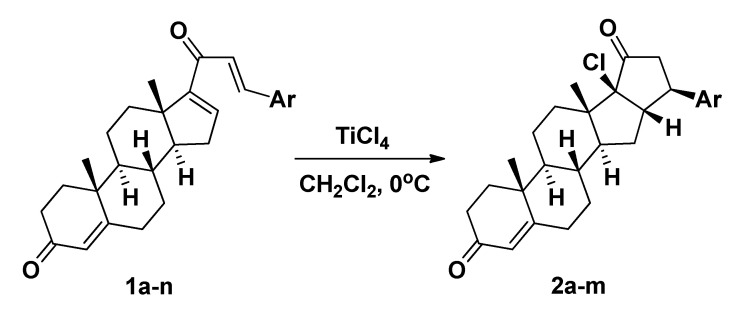
Synthesis of pentacyclic steroids **2a**–**m** by the interrupted Nazarov cyclization.

We started our research by studying a series of Lewis acids (TiCl_4_, SnCl_4_, AlCl_3_, FeCl_3_) and hydrogen chloride (HCl). 4-Chlorophenyl-substituted dienone **1c** was used as a model substrate and dichloromethane, dichloroethane, and benzene as the solvents. In all cases, except for titanium (IV) chloride, the reaction was very slow or gave an unidentified mixture that is difficult to separate. Thus, the reaction in the presence of aluminum and iron chlorides was very slow at room temperature and gave a multicomponent mixture of products at elevated temperatures (the monitoring of the reaction in the presence of AlCl_3_ is given in the SM, Figure S6). The reaction using tin (IV) chloride and hydrogen chloride affords a difficult-to-separate mixture of products even at 0 °C; a decrease in the temperature leads to a decrease in the reaction rate but does not improve the selectivity (see Table S1 in the Supplementary Materials (SM). Apparently, this is due to the low solubility of the steroid studied in dichloromethane at low temperatures (<−10 °C). Good results were obtained in the cyclization of benzylidenes in the presence of titanium (IV) chloride giving a single reaction product. The spectral analysis (mass spectrometry, ^1^H and ^13^C-NMR spectroscopy) of this product showed that it does not have a double bond but contains a chlorine atom. We suggested that, like the cyclization of camphor derived dienones [80], this reaction proceeds via interrupted Nazarov cyclization to form chloro-substituted pentacyclic steroid **2**. The optimization of the reaction conditions allowed us to synthesize products based on other benzylidenes in high yields (Scheme 3, Table 1). The reaction was performed in dichloromethane in the presence of freshly distilled titanium (IV) chloride as the catalyst under an inert atmosphere (argon) at a temperature from −5 °C to 20 °C (the catalyst was added at −5 to 0 °C). It should be noted that the use of freshly distilled TiCl_4_ is a necessary condition to achieve high yields. Besides, the solvent and the substrate should be thoroughly dried because the presence of even traces of moisture leads to a significant decrease in the yield of the desired products.

**Figure 1:**
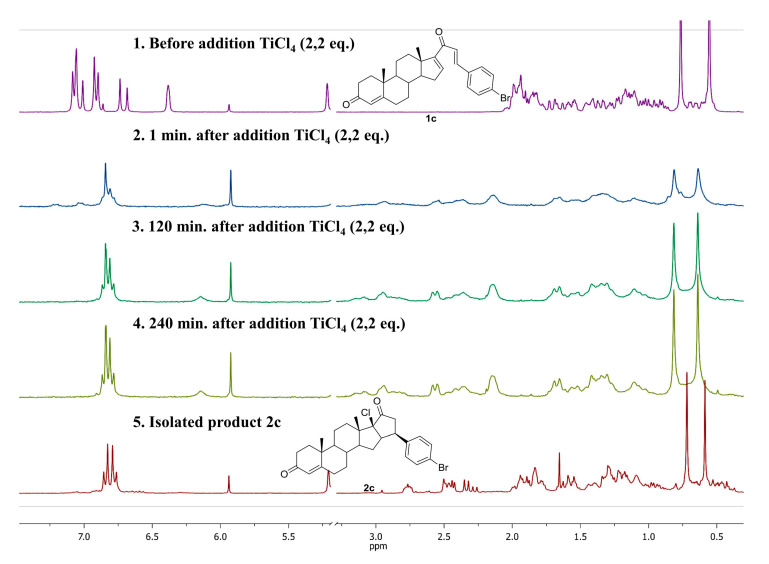
^1^H-NMR monitoring of the cyclization of benzylidene **1c**.

As can be seen in Table 1, the cyclization of benzylidenes was efficient and gave products in high yields, as evidenced by ^1^H-NMR analysis of the reaction mixture after the completion of the reaction (Table 1). The highest yields were obtained for halogen-substituted benzylidenes, the cyclization of which proceeds smoothly and with minimal side processes. The modest average yields of methoxy-substituted benzylidenes can be explained by the partial hydrolysis of methoxy groups promoted by a strong Lewis acid. Only the furan derivative was obtained in a low yield (35%) (Entry 14, Table 1). We failed to isolate this compound and characterize it as an individual product. Apparently, this is due to the high sensitivity of the furan derivative to Lewis acids, in particular, to titanium (IV) chloride, although the reaction of thiophenebenzylidene proved to be efficient and gave the target product in 64% yield. Despite high yields determined from the ^1^H NMR data, the isolated yields were moderate (Table 1). Significant losses during the purification by column chromatography are apparently due to the sensitivity of labile functional groups (tertiary alkyl chlorides, carbonyl groups, and activated methylene moiety) to silica gel. It is worth noting that the utilization of neutral alumina instead of silica gel did not lead to a significant improvement in the outcome. The efficiency of this process and the absence of by-products were also demonstrated by NMR monitoring of the cyclization of benzylidene **1c** (Figure 1).

As can be seen in Figure 1, the reaction is not accompanied by side processes and gives a single isomer, although we initially expected that this reaction would afford two isomers structurally similar to the *exo* and *endo* isomers prepared by the cyclization of camphor-derived dienones [80]. (The absence of any other by-products is evidenced by the presence of a single pair of signals both in the aromatic region and for methyl groups; for more information see Figure S4 in the SM). An analysis of the ^1^H and ^13^C-NMR spectra showed that the second isomer was absent in all other reactions as well (see copies of the ^1^H and ^13^C-NMR spectra of the synthesized pentacyclic steroids in the SM). We performed additional NMR studies, which confirmed the formation of a single conformer in solution (the ^1^H-NMR spectra were recorded in a wide temperature range from 20 to 100 °C, see Figure S5 in the SM). The heating of a solution of pentacyclic steroid **2m** in acetonitrile under reflux for 10 h also did not lead to any changes in the ^1^H NMR spectrum. The formation of a single conformer is confirmed by the ^1^H NMR spectrum of the crude mixture (see Figures S7 and S8 in the SM). The formation of one diastereomer can be explained by the rigidity of the steroid backbone, in which case the nucleophile always attacks with the formation of an *anti*-diastereomer. Similarly, the formation of a single diastereomer was observed in the case of bicyclic camphor and nopione structures [80].

### 2.2. Gram-Scale Synthesis of Pentacyclic Steroid ***2g***

**Scheme 4:**
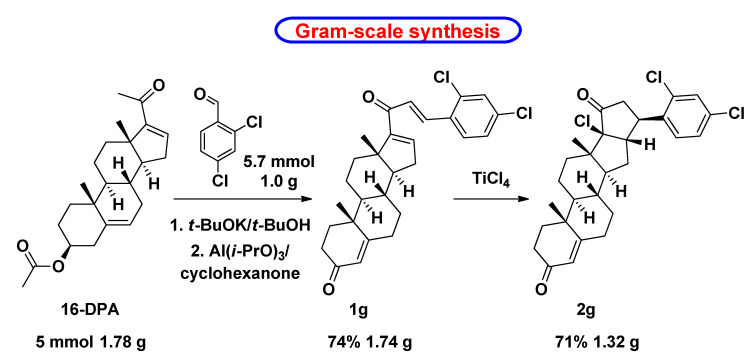
Gram-scale synthesis of pentacyclic steroid **2g**.

To demonstrate the scalability and application potential of the method for the synthesis of pentacyclic steroids, we performed a 5 mmol scale experiment for lead compound **2g**. The gram-scale synthetic protocol was carried out under the normal laboratory setup starting from commercially available 16-DPA under the optimized reaction conditions (Scheme 4).

To our delight, pentacyclic steroid **2g** was obtained without a significant decrease in yield (total 3 steps yield is 52%), which clearly demonstrates the practical applicability of our proposed method. (Benzylidene **1g** was obtained according to a previously developed method [53]).

### 2.3. X-ray Diffraction Study and Structure Optimization

**Figure 2:**
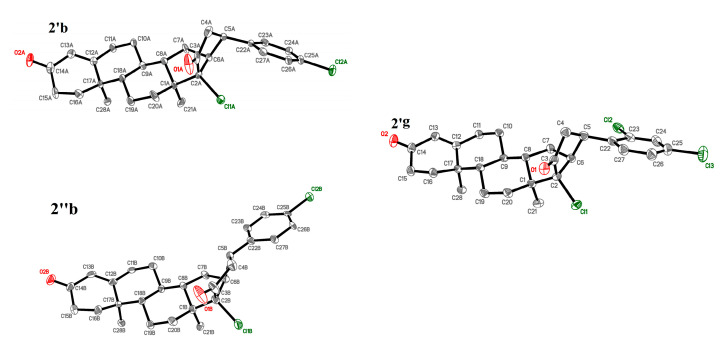
Crystal structures of **2b** (two conformers – **2′b** and **2″b**) and **2g** (a single conformer **2′g**). Two crystallographically independent molecules of **2′b** and molecule **2′g** are shown in a similar orientation, with thermal displacement ellipsoids drawn at the 50% probability level.

The crystal structures of products **2** were determined by X-ray diffraction study of compounds **2b** and **2g** (Figure 2) [88]. Absolute configurations of studied molecules were established by anomalous X-ray scattering in both cases, which allowed us not only to confirm configurations of unchanged chiral centers but also to find configurations of two new chiral centers generated during cyclization. The unit cell of compound **2b** comprises two conformers (**2′b** and **2″b**) in a ratio of 1:1 (Figure 2), whereas compound **2g** crystallizes as a single conformer **2′g** (Figure 2). 

The new chiral centers at the 16 and 17 positions of the steroid system (the C2 and C6 atoms in Figure 2) adopt the same configuration in molecules **2′b**, **2″b**, and **2′g**. The geometry of conformer **2′g** is similar to that of **2′b**. An analysis of the geometry shows that the presence of two conformers of **2b** can be nearly entirely attributed to the conformational flexibility of the terminal five-membered ring. It should be noted that the five-membered ring D and the six-membered rings A–C of the steroid system in the two conformers (**2′b** and **2″b**) have a very similar geometry. In both conformers, the terminal five-membered ring adopts an envelope conformation. The C5A atom bound to the phenyl group deviates from the plane defined by the other four atoms of the ring in **2′b**, whereas the C4B atom located between the carbonyl group and the >CHAr group deviates from the corresponding plane in **2″b**. An analysis of the van der Waals contacts in the conformationally different moieties of molecules **2′b** and **2″b** shows that intramolecular repulsive forces in these molecules are similar in strength (see Table S6 in the SM). Hence, the conformers should have similar energies. It is worth noting that there are numerous intermolecular non-valence interactions (H···H, O···H, C_Ph_···H_Ph_, Cl···Cl), which can affect the molecular geometry in the crystal. Intramolecular van der Waals repulsion in molecule **2′g** is somewhat stronger compared to **2′b**. Besides, there are short contacts between hydrogen atoms and the *ortho*-chlorine atom of the phenyl group (H5···Cl2 and H6···Cl2, Table S6 in the SM) in **2′g**. In all three molecules (**2′b**, **2″b**, and **2′g**), the aryl substituents are in a similar orientation to the C4–C5 bond of the five-membered ring; the C4–C5–C22–C27 torsion angles vary in the range of 15.7°–16.7°. It should be noted that undetected conformer **2″g**, which is structurally similar to **2″b** should be much less stable than **2′g** due to intramolecular van der Waals contacts (see section IV in the SM).

The relative energies of **2′b** and **2″b** and the energy of the transition state between these structures were evaluated by DFT calculations. The geometry optimization and energy calculations for **2′b** and **2″b** were performed at the ωB97X-D/6-311++G(d,p)//ωB97X-D/6-31+G(d,p) level of theory, according to the literature data [89]. The energy difference between the conformers is rather small (0.2 kcal/mol) and the energy barrier for conformational transitions is ca. 1.1 kcal/mol (see section V in the SM). Therefore, a low activation barrier makes it impossible to detect each conformer in solution by NMR methods even at low temperatures because of fast exchange on the NMR time scale.

**Figure 3:**
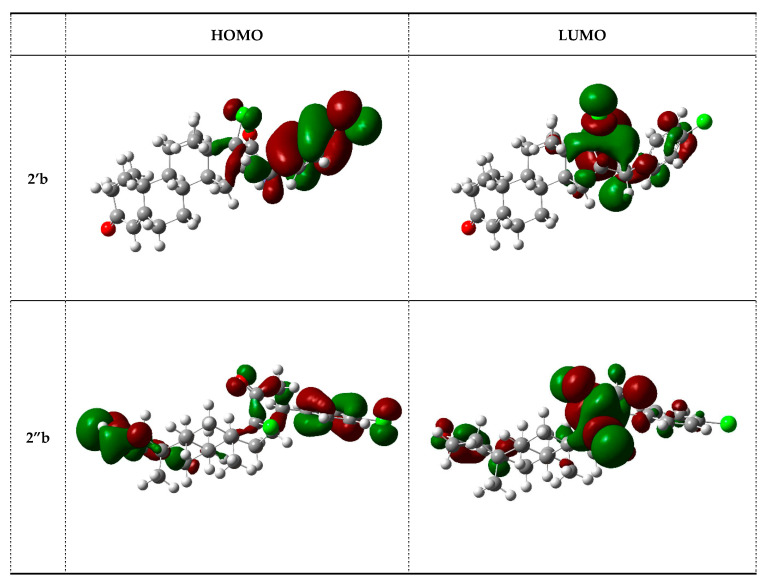
Localization of HOMO (**left**) and LUMO (**right**) for **2′b** (**top**) and **2″b** (**bottom**), calculated by the ωB97X-D functional for the gas phase.

Calculated frontier orbitals for conformers **2′b** and**2″b** are shown in Figure 3. It might be noted that both HOMO and LUMO orbitals are localized, to some extent, at carbon atoms of the newly formed 5-membered ring and also at chlorine (Cl1), oxygen (O1) atoms bound to atoms C2, C3 of the ring.

Therefore, different parts of this terminal 5-membered ring should be considered as reactive centers that should be susceptible to an attack by either nucleophilic or, to a lesser extent, electrophilic reagents. This conclusion will be tested in our further works.

### 2.4. In Vitro Antiproliferative Activity

Biological assays of the synthesized pentacyclic steroids were performed in the following two breast cancer cell lines: the most commonly used estrogen-dependent breast cancer cell line MCF-7 [90] and the triple-negative breast cancer cell line MDA-MB-231. Triple-negative breast cancer (TNBC) does not express estrogen and progesterone receptors and HER2/neu. Hence, the development of effective therapeutic agents for the treatment of TNBC is a challenge. Triple-negative breast cancer is generally characterized by rapid progression and does not respond to hormonal therapy [91]. We evaluated the antiproliferative activity of 13 synthesized compounds using standard incubation of the cells with the tested compounds for 72 h (Table 2).

All steroids caused 50% inhibition of proliferation of MCF-7 cells at concentrations lower than 25 µM. Compounds **2b**, **2d**, **2f**, and **2j** exhibited the highest activity with IC_50_ values below 8 µM. Antiproliferative effects of these compounds are comparable with that of cisplatin used as the reference compound. Four other pentacyclic steroids (**2a**, **c**, **g**, **i**) also showed high activity. For these compounds, the IC_50_ against MCF-7 cells was lower than 10 µM. The other steroids were less active and showed IC_50_ values ranging from 10 to 25 µM. Steroid **2b** exhibited the highest activity in the breast cancer cell line MCF-7. Hence, the compound containing the 4-chlorophenyl moiety (4-Cl-C_6_H_4_) as the aryl substituent is the most promising for further assays as an agent against hormone-dependent cancers.

The activity of the tested compounds against triple-negative breast cancer varied in a wide range. Four pentacyclic steroids (**2c,d,j,m**) were inactive and caused 50% inhibition of MDA-MB-231 cell growth at concentrations below 25 µM. On the contrary, compounds **2a,b,g** exhibited high activity against TNBC with IC_50_ lower than 10 µM. These steroids were more active than the reference compound cisplatin. Compounds **2f** and **2i** showed activity similar to that of cisplatin. Interestingly, 4-chlorophenyl-substituted steroid **2b**, which exhibited activity against the hormone-dependent cancer cell line MCF-7, showed relatively high activity against MDA-MB-231, but its effects were much weaker compared to **2g** (Table 2). Steroid **2g** was selected as the lead compound against MDA-MB-231. High antiproliferative activity of chlorine-containing steroids was also reported in the study [92]. The steroidal [17,16-*d*]pyrimidine derivative containing the 2-chlorophenyl moiety displayed a significant cytostatic effect in the cell lines HepG2 (human hepatocellular liver carcinoma cell line) and Huh7 (human hepatoma cell line) with IC_50_ values lower than 6 µM. This steroid was less active in the cell line SGC-7901 (human gastric cancer cell line), where it showed an effect with IC_50_ of ca. 10 µM. Probable mechanism of action of chlorine-containing steroids is considered in the study [93]. An androstanopyridine derivative bearing chlorine in the side chain was found to be an effective activator of the protein p53 in *in vitro* and *in vivo* experiments. The protein p53 is a key tumor suppressor and its directed activation can be considered as an approach to therapy using steroid derivatives, in particular pentacyclic steroids.

## 3. Materials and Methods

### 3.1. General Information 

Proton nuclear magnetic resonance spectra (^1^H-NMR) and carbon nuclear magnetic resonance spectra (^13^C-NMR) were recorded in deuterated solvents on a spectrometers (Fourier 300 HD, Bruker BioSpin AG, Zürich, Switzerland) working at 300 MHz for ^1^H, 75 MHz for ^13^C. Both ^1^H and ^13^C NMR reported in parts per million (ppm) at 293 K. Data are represented as follows: chemical shift, multiplicity (s, singlet; d, doublet; m, multiplet; br, broad; q, quartet), coupling constant in hertz (Hz). Melting points (mp) were recorded using an apparatus and not corrected. Mass spectra were obtained on a mass spectrometer (70 eV) with direct sample injection into the ion source. High resolution mass spectra were obtained from a TOF mass spectrometer with an SM source. All chemicals and anhydrous solvents were purchased from commercial sources and used without further purification. Silica column chromatography was performed using silica gel 60 (70−230 mesh); TLC analysis was conducted on silica gel 60 F254 plates. Benzylidines **1a**–**m** were prepared according to the previously reported method [53].

### 3.2. Synthesis and Characterization of Pentacyclic Steroids ***2a**–**m***

General method: To a stirring solution of benzylidine **1** (0.15 mmol) in abs methylene chloride (10 mL) at −5 °C the solution of freshly distilled titanium (IV) chloride (0.063 g, 0.33 mmol) in abs methylene chloride (5 mL) was added dropwise. The reaction mixture was stirred at the same temperature for 1.5 h and then it was kept at 20 °C until the reaction completion (monitoring by TLC). Then the reaction mixture was poured into the ice-water mixture (100 mL) and extracted with CH_2_Cl_2_ (3 × 20 mL). The combined organic phases were washed with water (2 × 50 mL), dried with sodium sulfate, and evaporated in a vacuum. The residue was purified by column chromatography by petroleum ester/ethyl acetate 3:1 and recrystallized from the same mixture.

#### 3.2.1. (6a*R*,6b*S*,8a*S*,8b*S*,11*R*,11a*S*,12a*S*,12b*R*)-8b-chloro-6a,8a-dimethyl-11-phenyl-1,6,6a,6b,7,8,8a,8b,10,11,11a,12,12a,12b-tetradecahydropentaleno[2,1-*a*]phenanthrene-4,9(2*H*,5*H*)-dione (**2a**)

Yield = 63% (0.041 g), given as a white powder; mp 203–205 °C; ^1^H-NMR (300 MHz, CDCl_3_): δ = 0.88–1.00 (m, 1H), 1.07 (s, 3H), 1.09–1.15 (m, 1H), 1.19 (s, 3H), 1.34–1.51 (m, 1H), 1.62–1.94 (m, 8H), 2.01–2.10 (m, 2H), 2.25–2.45 (m, 4H), 2.76 (dd, *J* = 18.2, 8.8 Hz,1H), 2.97–3.09 (m, 2H), 3.13–3.21 (m, 1H), 5.74 (s, 1H), 7.23–7.37 (m, 5H). ^13^C NMR (75 MHz, CDCl_3_): δ = 17.4, 17.5, 20.3, 30.4, 31.7, 32.5, 33.9, 35.4, 35.7, 36.6, 38.5, 45.0, 46.4, 48.6, 50.5, 53.1, 56.7, 79.0, 124.1, 126.8, 127.2 (2C), 128.6 (2C), 143.9, 170.1, 199.2, 211.6. HRMS (ESI-TOF) *m*/*z*: [M + H]^+^ Calcd for C_28_H_33_ClO_2_: 437.2237; Found: 437.2242.

#### 3.2.2. (6a*R*,6b*S*,8a*S*,8b*S*,11*R*,11a*S*,12a*S*,12b*R*)-11-(4-chlorophenyl)-8b-chloro-6a,8a-dimethyl-1,6,6a,6b,7,8,8a,8b,10,11,11a,12,12a,12b-tetradecahydropentaleno[2,1-*a*]phenanthrene-4,9(2*H*,5*H*)-dione (**2b**)

Yield = 78% (0.055 g), given as a light yellow powder; mp 125–127 °C; ^1^H-NMR (300 MHz, CDCl_3_): δ = 0.89–0.98 (m, 1H), 1.06 (s, 3H), 1.10–1.14 (m,1H), 1.20 (s, 3H), 1.38–1.49 (m, 1H), 1.65–1.91 (m, 8H), 2.00–2.19 (m, 2H), 2.27–2.42 (m, 4H), 2.78 (dd, *J* = 18.4, 9.0 Hz, 1H), 2.93–3.01 (m, 2H), 3.15–3.19 (m, 1H), 5.74 (s, 1H), 7.23 (d, *J* = 9 Hz, 2H), 7.27–7.31 (m, 2H). ^13^C-NMR (75 MHz, CDCl_3_): δ = 17.4, 17.4, 20.3, 30.3, 31.7, 32.5, 33.9, 35.4, 35.7, 36.6, 38.5, 44.8, 45.7, 48.8, 50.6, 53.1, 56.6, 78.9, 124.1, 128.6 (2C), 128.7 (2C), 132.6, 142.4, 169.9, 199.2, 211.3. HRMS (ESI-TOF) *m*/*z*: [M + H]^+^ Calcd for C_28_H_32_Cl_2_O_2_: 471.1844; Found: 471.1852.

#### 3.2.3. (6a*R*,6b*S*,8a*S*,8b*S*,11*R*,11a*S*,12a*S*,12b*R*)-11-(4-bromophenyl)-8b-chloro-6a,8a-dimethyl-1,6,6a,6b,7,8,8a,8b,10,11,11a,12,12a,12b-tetradecahydropentaleno[2,1-*a*]phenanthrene-4,9(2*H*,5*H*)-dione (**2c**)

Yield = 70% (0.046 g), given as a white powder; mp 123–125 °C; ^1^H-NMR (300 MHz, CDCl_3_): δ = 0.87–1.02 (m, 1H), 1.07 (s, 3H), 1.09–1.15 (m, 1H), 1.19 (s, 3H), 1.32–1.49 (m, 1H), 1.65–1.90 (m, 8H), 2.01–2.08 (m, 2H), 2.27–2.49 (m, 4H), 2.76 (dd, *J* = 18.5, 8.9 Hz, 1H), 2.99–3.07 (m, 2H), 3.10–3.20 (m, 1H), 5.74 (s, 1H), 7.18 (d, *J* = 8.3 Hz, 2H), 7.46 (d, *J* = 8.3 Hz, 2H). ^13^C-NMR (75 MHz, CDCl_3_): δ = 17.4, 17.4, 20.3, 30.3, 31.7, 32.5, 33.9, 35.4, 35.7, 36.6, 38.5, 44.7, 45.7, 48.8, 50.6, 53.1, 56.6, 78.9, 120.6, 124.1, 129.0 (2C), 131.7 (2C), 142.9, 170.0, 199.2, 211.4. HRMS (ESI-TOF) *m*/*z*: [M + H]^+^ Calcd for C_28_H_33_ClO_2_: 437.2237; Found: 437.2242.

#### 3.2.4. (6a*R*,6b*S*,8a*S*,8b*S*,11*R*,11a*S*,12a*S*,12b*R*)-11-(3-bromophenyl)-8b-chloro-6a,8a-dimethyl-1,6,6a,6b,7,8,8a,8b,10,11,11a,12,12a,12b-tetradecahydropentaleno[2,1-*a*]phenanthrene-4,9(2*H*,5*H*)-dione (**2d**)

Yield = 60% (0.046 g), given as a white powder; mp 230–232 °C; ^1^H-NMR (300 MHz, CDCl_3_): δ = 0.89–1.01 (m, 1H), 1.07 (s, 3H), 1.09–1.12 (m, 1H), 1.20 (s, 3H), 1.26–1.49 (m, 1H), 1.65–1.94 (m, 8H), 2.01–2.07 (m, 2H), 2.28–2.50 (m, 4H), 2.76 (dd, *J* = 18.3, 8.7 Hz, 1H), 2.96–3.03 (m, 2H), 3.14–3.20 (m, 1H), 5.74 (s, 1H), 7.18–7.23 (m, 2H), 7.38–7.40 (m, 1H), 7.45(s, 1H). ^13^C-NMR (75 MHz, CDCl_3_): δ = 17.4, 17.5, 20.3, 30.3, 31.7, 32.5, 33.9, 35.4, 35.7, 36.6, 38.5, 44.7, 45.9, 48.7, 50.5, 53.1, 56.5, 78.8, 122.7, 124.1, 125.7, 129.9, 130.2, 130.5, 146.2, 169.9, 199.2, 211.1. HRMS (ESI-TOF) *m*/*z*: [M + H]^+^ Calcd for C_28_H_32_BrClO_2_: 515.1359; Found: 515.1347.

#### 3.2.5. (6a*R*,6b*S*,8a*S*,8b*S*,11*R*,11a*S*,12a*S*,12b*R*)-11-(2-fluorophenyl)-8b-chloro-6a,8a-dimethyl-1,6,6a,6b,7,8,8a,8b,10,11,11a,12,12a,12b-tetradecahydropentaleno[2,1-*a*]phenanthrene-4,9(2*H*,5*H*)-dione (**2e**)

Yield = 40% (0.027 g), given as a white powder; mp 211–213 °C; ^1^H-NMR (300 MHz, CDCl_3_): δ = 0.89–1.00 (m, 1H), 1.06 (s, 3H), 1.09–1.17 (m, 1H), 1.20 (s, 3H), 1.26–1.49 (m, 1H), 1.65–1.92 (m, 8H), 2.01–2.16 (m, 2H), 2.28–2.48 (m, 4H), 2.79 (dd, *J* = 18.8, 9.0 Hz, 1H), 2.95–3.03 (m, 2H), 3.54–3.57 (m, 1H), 5.75 (s, 1H), 7.03–7.15 (m, 2H), 7.21–7.31 (m, 2H). ^13^C NMR (75 MHz, CDCl_3_): δ = 17.3, 17.4, 20.3, 30.3, 31.7, 32.5, 33.9, 35.3, 35.7, 36.6, 38.6 (d, ^3^*J*(C-F) = 3.1 Hz), 38.7, 43.5, 49.0, 50.7, 53.1, 55.3, 78.8, 115.3 (d, ^2^*J*(C-F) = 21.9 Hz), 124.1, 124.1, 127.9 (d, ^3^*J*(C-F) = 4.1 Hz), 128.4 (d, ^3^*J*(C-F) = 8.4 Hz), 130.7 (d, ^2^*J*(C-F) = 13.9 Hz), 160.6 (d, ^1^*J*(C-F) = 245.3 Hz), 170.1, 199.2, 212.0. HRMS (ESI-TOF) *m*/*z*: [M + H]^+^ Calcd for C_28_H_32_FClO_2_: 455.2149; Found: 455.2148.

#### 3.2.6. (6a*R*,6b*S*,8a*S*,8b*S*,11*R*,11a*S*,12a*S*,12b*R*)-8b-chloro-11-(4-fluorophenyl)-6a,8a-dimethyl-1,6,6a,6b,7,8,8a,8b,10,11,11a,12,12a,12b-tetradecahydropentaleno[2,1-*a*]phenanthrene-4,9(2*H*,5*H*)-dione (**2f**)

Yield = 71% (0.048 g), given as a white powder; mp 181–183 °C; ^1^H-NMR (300 MHz, CDCl_3_): δ = 0.89–1.00 (m, 1H), 1.06 (s, 3H), 1.09–1.14 (m, 1H), 1.19 (s, 3H), 1.34–1.49 (m, 1H), 1.64–1.90 (m, 8H), 1.99–2.11 (m, 2H), 2.27–2.49 (m, 4H), 2.76 (dd, *J* = 18.4, 8.9 Hz, 1H), 2.93–3.01 (m, 2H), 3.13–3.23 (m, 1H), 5.74 (s, 1H), 6.99–7.05 (m, 2H), 7.23–7.28 (m, 2H). ^13^C NMR (75 MHz, CDCl_3_): δ = 17.4, 17.4, 20.3, 30.3, 31.7, 32.5, 33.9, 35.4, 35.6, 36.6, 38.5, 45.0, 45.5, 48.8, 50.5, 53.1, 56.8, 78.9, 115.4 (d, ^2^*J*(C-F) = 21.4 Hz, 2C), 124.1, 128.7 (d, ^3^*J*(C-F) = 8.0 Hz, 2C), 139.6 (d, ^4^*J*(C-F) = 3.0 Hz), 161.6 (d, ^1^*J*(C-F) = 245.3 Hz), 170.1, 199.4, 211.7. HRMS (ESI-TOF) *m*/*z*: [M + H]^+^ Calcd for C_28_H_32_FClO_2_: 455.2155; Found: 455.2148.

#### 3.2.7. (6a*R*,6b*S*,8a*S*,8b*S*,11*R*,11a*S*,12a*S*,12b*R*)-8b-chloro-11-(2,4-dichlorophenyl)-6a,8a-dimethyl-1,6,6a,6b,7,8,8a,8b,10,11,11a,12,12a,12b-tetradecahydropentaleno[2,1-*a*]phenanthrene-4,9(2*H*,5*H*)-dione (**2g**)

Yield = 73% (0.055 g), given as a light yellow powder; mp 185–187 °C; ^1^H-NMR (300 MHz, CDCl_3_): δ = 0.89–0.98 (m, 1H), 1.05 (s, 3H), 1.10–1.14 (m, 1H), 1.20 (s, 3H), 1.39–1.49 (m, 1H), 1.65–1.89 (m, 8H), 2.03–2.21 (m, 2H), 2.29–2.43 (m, 4H), 2.89–2.96 (m, 3H), 3.68–3.74 (m, 1H), 5.75 (s, 1H, CH), 7.19–7.25 (m, 2H), 7.42 (s, 1H). ^13^C-NMR (75 MHz, CDCl_3_): δ = 17.1, 17.4, 20.2, 30.3, 31.7, 32.5, 33.9, 35.3, 35.7, 36.7, 38.5, 41.9, 43.8, 49.2, 50.8, 53.1, 55.0, 78.7, 124.2, 127.1, 128.7, 129.3, 133.1, 134.4, 139.8, 170.0, 199.2, 212.3. HRMS (ESI-TOF) *m*/*z*: [M + H]^+^ Calcd for C_28_H_31_Cl_3_O_2_: 505.1462; Found: 505.1462.

#### 3.2.8. (6a*R*,6b*S*,8a*S*,8b*S*,11*R*,11a*S*,12a*S*,12b*R*)-8b-chloro-11-(2-chloro-6-fluorophenyl)-6a,8a-dimethyl-1,6,6a,6b,7,8,8a,8b,10,11,11a,12,12a,12b-tetradecahydropentaleno[2,1-*a*]phenanthrene-4,9(2*H*,5*H*)-dione (**2h**)

Yield = 58% (0.042 g), given as a light yellow powder; mp 164–166 °C; ^1^H-NMR (300 MHz, CDCl_3_): δ = 0.93–0.99 (m, 1H), 1.09 (s, 3H), 1.12–1.17 (m, 1H), 1.20 (s, 3H), 1.39–1.51 (m, 1H), 1.63–1.98 (m, 9H), 2.02–2.09 (m, 2H), 2.28–2.45 (m, 4H), 2.57 (ddd, *J* = 17.8, 8.9, 3.4 Hz, 1H), 3.03–3.08 (m, 1H), 3.38 (dd, *J* = 17.8, 8.8 Hz, 1H), 3.60–3.67 (m,1H), 5.75 (s, 1H), 6.97–7.05 (m, 1H), 7.15–7.25 (m, 2H). ^13^C NMR (75 MHz, CDCl_3_): δ = 17.4, 17.6, 20.4, 30.9, 31.7, 32.5, 33.9, 34.5, 35.6, 36.4, 38.5, 40.4, 42.1 (d, ^3^*J*(C-F) = 7.3 Hz), 48.5, 50.7, 53.0, 55.3, 79.0, 115.3 (d, ^2^*J*(C-F) = 23.7 Hz), 124.0, 125.7 (d, ^4^*J*(C-F) = 2.9 Hz), 128.0 (d, ^2^*J*(C-F) = 14.3 Hz), 128.7 (d, ^3^*J*(C-F) = 10.0 Hz), 134.9 (d, ^3^*J*(C-F) = 7.0 Hz), 161.5 (d, ^1^*J*(C-F) = 249.5 Hz), 170.0, 199.3, 210.4. HRMS (ESI-TOF) *m*/*z*: [M + H]^+^ Calcd for C_28_H_31_Cl_2_FO_2_: 489.1751; Found: 489.1758.

#### 3.2.9. (6a*R*,6b*S*,8a*S*,8b*S*,11*R*,11a*S*,12a*S*,12b*R*)-8b-chloro-11-(4-methoxyphenyl)-6a,8a-dimethyl-1,6,6a,6b,7,8,8a,8b,10,11,11a,12,12a,12b-tetradecahydropentaleno[2,1-*a*]phenanthrene-4,9(2*H*,5*H*)-dione (**2i**)

Yield = 55% (0.038 g), given as an yellow powder; mp 180–182 °C; ^1^H-NMR (300 MHz, CDCl_3_): δ = 1.02 (s, 3H), 1.08–1.14 (m, 1H), 1.22 (s, 3H), 1.32–1.54 (m, 2H), 1.64–1.93 (m, 8H), 2.02–2.09 (m, 1H), 2.25–2.45 (m, 5H), 2.80–2.85 (m, 1H), 2.95–2.99 (m, 1H), 3.04–3.12 (m, 2H), 3.82 (s, 3H), 5.74 (s, 1H), 6.90 (d, *J* = 8.2 Hz, 2H), 7.19 (d, *J* = 8.3 Hz, 2H). ^13^C NMR (75 MHz, CDCl_3_): δ = 16.3, 17.5, 20.3, 31.8, 32.6, 33.4, 33.9, 34.0, 35.5, 35.7, 38.5, 46.5, 47.6, 48.6, 52.8, 52.9, 55.3, 61.4, 83.1, 114.3 (2C), 124.1, 127.6 (2C), 134.3, 158.7, 170.3, 199.2, 210.3. HRMS (ESI-TOF) *m*/*z*: [M + H]^+^ Calcd for C_29_H_35_Cl_2_O_3_ 467.2345; Found: 467.2347.

#### 3.2.10. (6a*R*,6b*S*,8a*S*,8b*S*,11*R*,11a*S*,12a*S*,12b*R*)-8b-chloro-11-(3-methoxyphenyl)-6a,8a-dimethyl-1,6,6a,6b,7,8,8a,8b,10,11,11a,12,12a,12b-tetradecahydropentaleno[2,1-*a*]phenanthrene-4,9(2*H*,5*H*)-dione (**2j**)

Yield = 44% (0.030 g), given as an yellow powder; mp 126–128 °C; ^1^H NMR (300 MHz, CDCl_3_): δ = 0.90–1.00 (m, 1H), 1.08 (s, 3H), 1.12–1.16 (m, 1H), 1.21 (s, 3H), 1.36–1.50 (m, 1H), 1.63–1.95 (m, 8H), 2.01–2.10 (m, 2H), 2.29–2.45 (m, 4H), 2.75 (dd, *J* = 18.2, 8.7 Hz, 1H), 2.98–3.08 (m, 2H), 3.11–3.18 (m, 1H), 3.83 (s, 3H), 5.75 (s, 1H), 6.78–6.82 (m, 1H), 6.86–6.91 (m, 2H), 7.24–7.29 (m, 1H). ^13^C-NMR (75 MHz, CDCl_3_): δ = 17.4, 17.5, 20.3, 30.4, 31.7, 32.5, 33.9, 35.5, 35.7, 36.6, 38.5, 45.0, 46.4, 48.6, 50.5, 53.1, 55.2, 56.6, 79.1, 111.9, 113.2, 119.5, 124.1, 129.7, 145.6, 159.8, 170.1, 199.3, 211.6. HRMS (ESI-TOF) *m*/*z*: [M + H]^+^ Calcd for C_29_H_35_ClO_3_: 467.2333; Found: 467.2347.

#### 3.2.11. (6a*R*,6b*S*,8a*S*,8b*S*,11*R*,11a*S*,12a*S*,12b*R*)-8b-chloro-11-(3,4-dimethoxyphenyl)-6a,8a-dimethyl-1,6,6a,6b,7,8,8a,8b,10,11,11a,12,12a,12b-tetradecahydropentaleno[2,1-*a*]phenanthrene-4,9(2*H*,5*H*)-dione (**2k**)

Yield = 43% (0.032 g), given as an yellow powder; mp 182–184 °C; ^1^H-NMR (300 MHz, CDCl_3_): δ = 0.89–0.98 (m, 1H), 1.07 (s, 3H), 1.12–1,16 (m,1H), 1.20 (s, 3H), 1.35–1.49 (m, 1H), 1.65–1.91 (m, 8H), 2.00–2.09 (m, 2H), 2.27–2.49 (m, 4H), 2.77 (dd, *J* = 18.6, 9.0 Hz, 1H), 2.93–3.00 (m, 2H), 3.11–3.18 (m, 1H), 3.87 (s, 3H), 3.89 (s, 3H), 5.74 (s, 1H), 6.80–6.85 (m, 3H). ^13^C NMR (75 MHz, CDCl_3_): δ = 17.4, 17.5, 20.3, 30.4, 31.7, 32.5, 33.9, 35.6, 35.7, 36.0, 36.6, 38.5, 45.4, 46.1, 48.8, 50.5, 53.1, 55.9 (2C), 56.7, 79.2, 110.5, 111.1, 119.2, 124.1, 136.7, 147.9, 149.1, 170.0, 199.2, 212.0. HRMS (ESI-TOF) *m*/*z*: [M + H]^+^ Calcd for C_30_H_37_ClO_4_: 497.2447; Found: 497.2453.

#### 3.2.12. (6a*R*,6b*S*,8a*S*,8b*S*,11*R*,11a*S*,12a*S*,12b*R*)-8b-chloro-11-(3,4,5-trimethoxyphenyl)-6a,8a-dimethyl-1,6,6a,6b,7,8,8a,8b,10,11,11a,12,12a,12b-tetradecahydropentaleno[2,1-*a*]phenanthrene-4,9(2*H*,5*H*)-dione (**2l**)

Yield = 47% (0.037 g), given as an yellow powder; mp 207–209 °C; ^1^H-NMR (300 MHz, CDCl_3_): δ = 0.89–1.00 (m, 1H), 1.09 (s, 3H), 1.12–1.16 (m, 1H), 1.20 (s, 3H), 1.40–1.48 (m, 1H), 1.65–1.91 (m, 8H), 2.02–2.10 (m, 2H), 2.27–2.44 (m, 4H), 2.79 (dd, *J* = 18.6, 9.1 Hz, 1H), 2.92–3.01 (m, 2H), 3.10–3.16 (m, 1H), 3.84 (s, 3H), 3.87 (s, 6H), 5.74 (s, 1H), 6.52 (s, 2H). ^13^C-NMR (75 MHz, CDCl_3_): δ = 17.5, 17.6, 20.4, 30.5, 31.8, 32.6, 33.9, 35.7, 36.0, 36.7, 38.6, 45.6, 47.0, 48.9, 50.6, 53.2, 56.2 (2C), 56.6, 60.9, 79.4, 104.5 (2C), 124.2, 136.9, 140.1, 153.3(2C) 170.1, 199.3, 212.1. HRMS (ESI-TOF) *m*/*z*: [M + H]^+^ Calcd for C_31_H_39_ClO_5_: 527.2551; Found: 527.2559.

#### 3.2.13. (6a*R*,6b*S*,8a*S*,8b*S*,11*R*,11a*S*,12a*S*,12b*R*)-8b-chloro-6a,8a-dimethyl-11-(thiophen-2-yl)-1,6,6a,6b,7,8,8a,8b,10,11,11a,12,12a,12b-tetradecahydropentaleno[2,1-*a*]phenanthrene-4,9(2*H*,5*H*)-dione (**2m**)

Yield = 64% (0.042 g), given as a light yellow powder; mp 193–195 °C; ^1^H-NMR (300 MHz, CDCl_3_): δ = 0.89–0.98 (m, 1H), 1.05–1.15 (m, 4H), 1.20 (s, 3H), 1.37–1.48 (m, 1H), 1.60–1.93 (m, 8H), 2.02–2.16 (m, 2H), 2.25–2.47 (m, 4H), 2.83 (dd, *J* = 17.6, 7.4 Hz, 1H), 2.99–3.17 (m, 2H), 3.42–3.54 (m, 1H), 5.74 (s, 1H), 6.86–6.98 (m, 2H), 7.17–7.24 (m, 1H). ^13^C NMR (75 MHz, CDCl_3_): δ = 17.4, 17.5, 20.4, 30.4, 31.8, 32.6, 33.9, 34.9, 35.7, 36.7, 38.6, 41.3, 45.6, 49.0, 50.8, 53.2, 56.9, 78.5, 123.9, 124.0, 124.2,126.9, 147.8, 170.1, 199.3, 211.1. HRMS (ESI-TOF) *m*/*z*: [M + H]^+^ Calcd for C_26_H_31_ClO_2_S 443.1801; Found: 443.1806.

## 4. Conclusions

In summary, we studied the cyclization of 16-DPA-based benzylidenes in the presence of Lewis or Bronsted acids, in particular, aluminum, iron (III), tin, titanium (IV) chlorides and dry hydrogen chloride. It was found that these compounds readily undergo highly regioselective interrupted Nazarov cyclization with trapping chloride ion. An efficient method of the synthesis of D-annulated pentacyclic steroids was developed. The structures of the synthesized pentacyclic steroids were determined by NMR and X-ray diffraction. It was found that the reaction affords a single diastereomer, but it can crystallize as two conformers depending on the structure. The formation of one diastereomer can be explained by the rigidity of the steroid backbone, in which case the nucleophile always attacks with the formation of an antidiastereomer. Similarly, the formation of a single diastereomer was observed in the case of bicyclic camphor and nopione structures. The synthetic potential of this protocol has been illustrated by the gram-scale experiment. Antiproliferative activity of the synthesized compounds was evaluated against two breast cancer cell lines: MCF-7 and MDA-MB-231. All the tested compounds exhibit relatively high antiproliferative activity. The activity of a number of steroids is comparable to that of cisplatin used as the reference compound. Besides, we expected that the steroid compounds with high antiproliferative activity would be less toxic to normal tissues compared to cisplatin, which displays serious side effects significantly complicating the treatment process. A comparison of the antiproliferative activity of the newly synthesized pentacyclic steroids with that of the starting benzylidenes (see [51]) shows that the Nazarov cyclization of the latter does not lead to a loss of activity. Further preclinical and clinical trials are required to identify intracellular targets for the lead compounds (**2b** and **2g**). In particular, it remains to determine whether the selected molecules affect steroid hormone receptors and steroid metabolism enzymes in luminal breast cancer cells and to analyze whether p53 is involved in the cytotoxic effect caused by new-class steroids.

## Figures and Tables

**Table 1:** Yields of benzylidene cyclization products **2a**–**n**.

Entry	Codes	Ar	Yields	
			NMR *	Isolated
1	a	Ph	80	63
2	b	4-Cl-C_6_H_4_	95	78
3	c	4-Br-C_6_H_4_	95	70
4	d	3-Br-C_6_H_4_	88	60
5	e	2-F-C_6_H_4_	71	40
6	f	4-F-C_6_H_4_	80	41
7	g	2,4-Cl_2_C_6_H_3_	92	73
8	h	2-Cl-6-F-C_6_H_3_	80	58
9	i	4-MeO-C_6_H_4_	78	55
10	j	3-MeO-C_6_H_4_	76	44
11	k	3,4-(MeO)_2_-C_6_H_3_	91	43
12	l	3,4,5-(MeO)_3_C_6_H_2_	70	47
13	m	2-Thienyl	92	64
14	n	2-Furyl	35	0

* TMS is used as internal standard.

**Table 2:** Antiproliferative activity of pentacyclic steroids **2a**–**m** and cisplatin (cell growth for 72 h).

IC_50_^a^, µM			
Entry	Compounds	MCF-7	MDA-MB-231
1	2a	10.0 ± 1.5	8.8 ± 0.9
2	2b	6.0 ± 0.8	9.4 ± 1.1
3	2c	8.1 ± 0.9	>25
4	2d	7.5± 0.9	>25
5	2e	12.6 ± 1.6	19.6 ± 2.1
6	2f	7.1 ± 0.9	12.2 ± 1.6
7	2g	8.2 ± 0.9	6.3 ± 0.7
8	2h	17.7 ± 2.1	15.2 ± 1.6
9	2i	9.5 ± 1.2	13.0 ± 1.5
10	2j	7.2 ± 0.8	>25
11	2k	25 ± 2.2	24.8 ± 2.5
12	2l	10.6 ± 1.3	21.5 ± 2.4
13	2m	24.5 ± 2.5	>25
14	Cisplatin(CDDP)	6.3 ± 0.8	13.2 ± 1.5

IC_50_^a^ is the concentration that causes 50% inhibition of cell growth.

## Supplementary Materials

The following are available online biological assays, X-ray diffraction studies, DFT calculations, transition state, thermodynamic calculations, ^1^H NMR monitoring, copies of ^1^H and ^13^C NMR spectra, copies of HRMS spectra.
