# Supplementary material for: Novel d-Annulated Pentacyclic Steroids: Regioselective Synthesis and Biological Evaluation in Breast Cancer Cells
Source: Molecules. 2020 Jul 31;25(15):3499. doi: 10.3390/molecules25153499 (PMC7435891; doi:10.3390/molecules25153499)
Supplement: Supplementary file 1 [file molecules-25-03499-s001.pdf]

Supporting Information

Novel D-Annulated Pentacyclic Steroids: Regioselective Synthesis and Biological Evaluation in Breast Cancer Cells

Svetlana K. Vorontsova,[1] Anton V. Yadykov,[1] Alexander M. Scherbakov,[2] Mikhail E. Minyaev,[1] Igor V. Zavarzin,[1] Ekaterina I. Mikhaevich,[2] Yulia A. Volkova,[1]* Valerii Z. Shirinian[1]

[1]N. D. Zelinsky Institute of Organic Chemistry, Russian Academy of Sciences, Leninsky prosp. 47, 119991 Moscow, Russia

[2]N. N. Blokhin National Medical Research Center of Oncology, Kashirskoye shosse 24, 115522 Moscow, Russia

Table of Contents

I. Table S1. Optimization of Nazarov reaction conditions. 2

II. Biological assays. 3

III. X-ray diffraction studies 4

IV.1. DFT calculations 13

IV.2. Transition state 17

IV.3. Thermodynamic calculations 19

V. ^{1}H NMR monitoring 20

VI. Copies of ^{1}H and ^{13}C NMR spectra 25

VII. Copies of HRMS specta. 38

VIII. References 51

I. Table S1. Optimization of Nazarov reaction conditions.

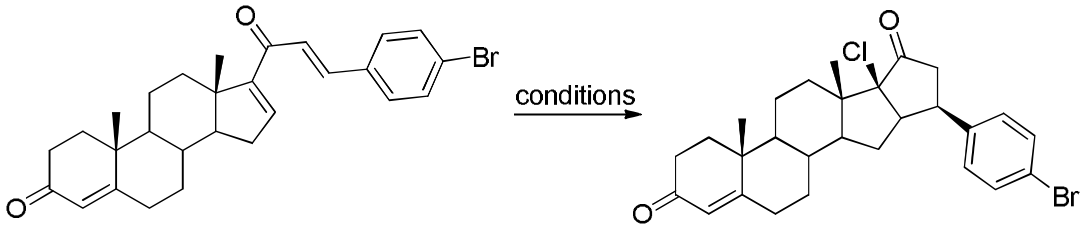

1c **2c**

Catalyst/Solvent	Isolated yields
$AlCl_3/CH_2Cl_2$	49
$TiCl_4/CH_2Cl_2$	70
$SnCl_4/CH_2Cl_2$	38
$FeCl_3/CH_2Cl_2$	23
$HCl_{(gas)}/CH_2Cl_2$	N/A*

*the starting benzylidene **1c** was recovered

II. Biological assays.

General procedure.

The breast cancer cell lines MCF-7 and MDA-MB-231 were obtained from the ATCC collection and were used to evaluate the antiproliferative activity of the synthesized compounds. The cultivation of the cells was performed in standard (4.5 g/L glucose) DMEM medium (Gibco) supplemented with 10% fetal bovine serum (HyClone), 0.1 mg/ml sodium pyruvate (Santa Cruz), 50 U/ml penicillin, and 50 µg/ml streptomycin. Cells were incubated at 37°C in the presence of 5% CO_2 at a relative humidity of 85–90% in a NuAire incubator.

The synthesized compounds were dissolved in dimethyl sulfoxide (DMSO) to a concentration of 5 mM using sonication, and the solutions were stored at –20°C before use. The MCF-7 and MDA-MB-231 cells were seeded onto a 24-well plate (Corning) at a density of $4*10^4$ cells per well. After growth overnight, the compounds were added to the cells in the concentration range from 1.5 to 25 µM. Cisplatin was used as the reference compound. An appropriate solvent (DMSO) volume was added to the control cells; the final concentration of the solvent in the medium was less than 0.5%. The antiproliferative activity was evaluated using the MTT assay, which is based on the reduction of the MTT reagent (3-(4,5-dimethylthiazol-2-yl)-2,5-diphenyltetrazolium bromide) in living cells giving violet formazan crystals insoluble in the culture medium; [S1] the MTT assay was performed in a modified version as described previously.[S2]

After 72 h growth in the presence of the tested compounds, the medium was removed and the MTT reagent (AppliChem) was added to the cells. After 2 h incubation with the MTT reagent, the cells were lysed in 100% DMSO (AppliChem). The plate was shaken to dissolve the formazan crystals that formed. The absorbance of the solutions was measured on a MultiScan FC spectrophotometer at 571 nm. Then the blank absorbance values (media only) were subtracted from the sample absorbance values; the absorbance of the solutions of the control samples was taken as 100%. The IC_{50} values were calculated as the concentration of the compound that decreases the absorbance of the solution by 50% compared to the control sample using the GraphPad software. The experiments were repeated in triplicate.

III. X-ray diffraction studies

X-ray diffraction data were collected at 100K on a Bruker Quest D8 diffractometer equipped with a Photon-III area-detector (graphite monochromator, shutterless φ- and ω-scan technique), using Mo K_{α}-radiation (0.71073 Å). The intensity data were integrated by the SAINT program [S3] and were corrected for absorption (semi-empirical from equivalents by multi-scan techniques) using TWINABS[S3] for **2b** and SADABS [S4] for **2g**.The structures were solved by direct methods using SHELXT/SHELXS-2013 [S5] and refined by full-matrix least-squares on F^2 using SHELXL-2018. [S6] All non-hydrogen atoms were refined with anisotropic displacement parameters. Hydrogen atoms were placed in ideal calculated positions and refined as riding atoms with relative isotropic displacement parameters.A rotating group model was applied for methyl groups.The studied crystal of **2b** was refined as a 2-component twin with the twin law of [-1 0.01 0, 0.14 1 0.01, 0 0.01 -1] (the second domain was rotated from the first domain by 178° about reciprocal axis 0 1 0.01). The domain ratio was not found based on collected data since the total number of collected reflections (127213 at resolution down to 0.69Å) contained less than 0.18% single reflections (225) corresponding to the first domain and no single reflection for the second domain; all other reflections were composites of both domains. A non-coordinating methanol molecule in **2g** was disordered over at least four overlapping positions with the overall occupancy of 0.5, forming infinite 1D chains in the crystal channels of **2g**. This molecule was removed by the SQUEEZE method [S7] implemented in the PLATON program. [S8] The absolute structures of chiral centers unchanged in course of reactions were confirmed by anomalous X-ray scattering. The absolute structure parameter (Flack x) was determined by classical fit [S9] for the twinned crystal **2b**:$I_H(calc)=(1-x)|F_H(calc)|^2+x|F_{-H}(calc)|^2$, and by using 3396 quotients [S10] [(I+)-(I-)]/[(I+)+(I-)] for **2g**. The SHELXTL program suite [S3] was used for molecular graphics. Crystal data, data collection and structure refinement details for **2'b** and **2''b** are summarized in Table S1.

Table S2. Crystal data and structure refinement for **2b** and **2g**.

Identification code	**2b**	**2g**
Empirical formula	$C_{28}H_{32}Cl_2O_2$	$C_{28}H_{31}Cl_3O_2 \cdot 1/2(CH_4O)$
Formula weight	471.43	521.90
Crystal system	Orthorhombic	Hexagonal
Space group	$P2_12_12_1$	$P6_5$
Unit cell dimensions		
a, Å	7.3635(4)	19.0193(2)
b, Å	24.3804(13)	19.0193(2)
c, Å	25.9800(13)	12.2874(2)
α, β, γ,°	90, 90, 90	90, 90, 120
Volume, $Å^3$	4664.1(4)	3849.28(10)
Z	8	6
Calcd. density, g/cm^3	1.343	1.351
Absorption coefficient, mm^{-1}	0.302	0.384
F(000)	2000	1650
Crystal size, mm	0.23 × 0.15 × 0.15	0.19 × 0.18 × 0.03
Θ range for data collection	2.291 to 29.000°.	2.473 to 30.524°.
Index ranges	0<=h<=10,	-27<=h<=27,
	0<=k<=33,	-27<=k<=27,
	0<=l<=35	-17<=l<=17
Reflections collected	6898	107410
Independent reflections [R(int)]	6898 [-]	7847 [0.0433]
Observed reflections [I>2σ(I)]	4498	7437
Completeness to Θfull / max	99.9 / 99.9 %	99.8 / 99.9 %
Max. and min. transmission	0.64617 and 0.30068	0.5722 and 0.5502
Data / restraints / parameters	6898 / 0 / 582	7847 / 1 / 300
Goodness-of-fit on F^2	1.068	1.055
Final R1 / wR2 indices [I>2σ(I)]	0.0813 / 0.1529	0.0277 / 0.0671
Final R1 / wR2 indices (all data)	0.1395 / 0.1811	0.0308 / 0.0697
Absolute structure parameter	0.22(11)	-0.019(11)
Largest diff. peak / hole, $e \cdot Å^{-3}$	0.714 / -0.481	0.290/ -0.272
CCDC number	1990621	1990622

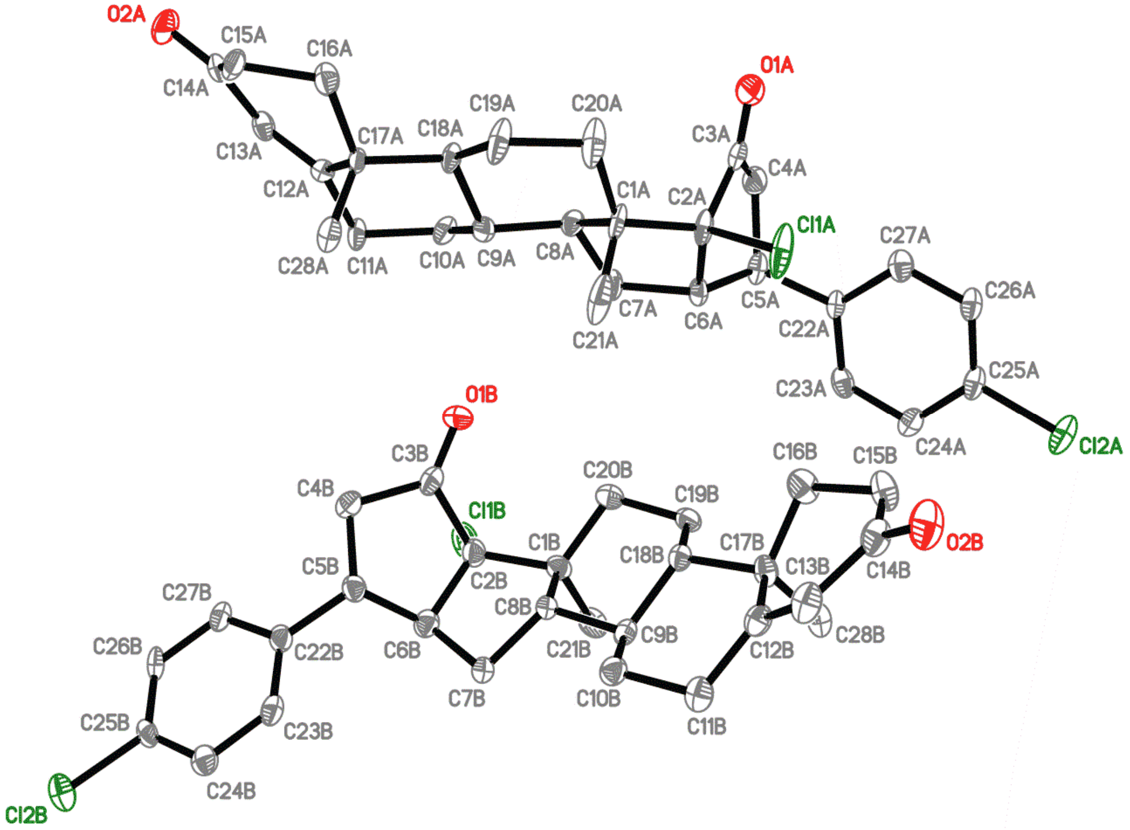

Fig. S1. Two crystallographically nonequivalent molecules of **2b** and their mutual positions. Hydrogen atoms are not shown.

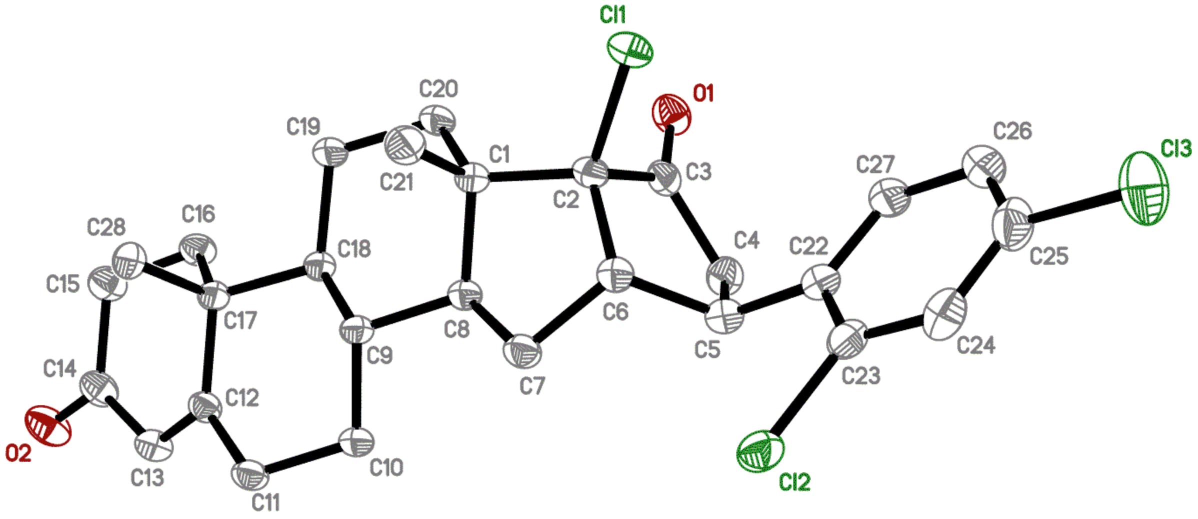

Fig. S2. Crystal structure of **2g**. Hydrogen atoms are not shown.

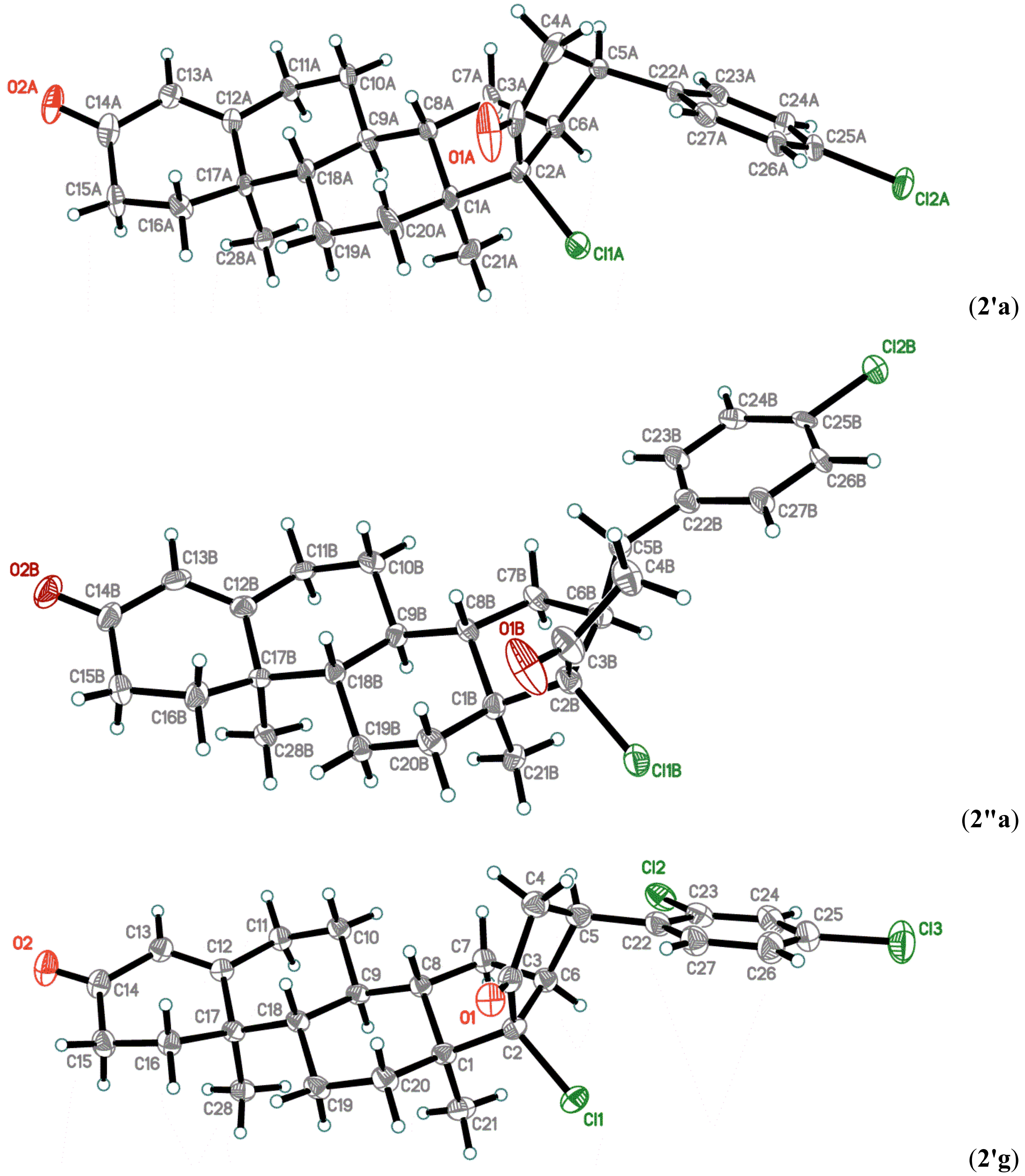

Fig. S3. Conformations of the terminal 5-membered ring in **2b** (**2'b**, top and **2"b**, middle) and in **2g** (conformation **2'g**, bottom). All molecules are similarly oriented for comparison.

Table S3. Bond lengths in **2b**, Å.

Cl(1A)-C(2A)	1.811(6)	Cl(1B)-C(2B)	1.815(7)
Cl(2A)-C(25A)	1.746(6)	Cl(2B)-C(25B)	1.755(6)
O(1A)-C(3A)	1.191(8)	O(1B)-C(3B)	1.199(8)
O(2A)-C(14A)	1.230(7)	O(2B)-C(14B)	1.226(7)
C(1A)-C(20A)	1.510(9)	C(1B)-C(20B)	1.525(8)
C(1A)-C(21A)	1.524(10)	C(1B)-C(21B)	1.530(9)
C(1A)-C(8A)	1.532(8)	C(1B)-C(8B)	1.539(8)
C(1A)-C(2A)	1.547(8)	C(1B)-C(2B)	1.557(9)
C(2A)-C(3A)	1.537(10)	C(2B)-C(3B)	1.537(9)
C(2A)-C(6A)	1.551(9)	C(2B)-C(6B)	1.571(9)
C(3A)-C(4A)	1.519(10)	C(3B)-C(4B)	1.505(9)
C(4A)-C(5A)	1.525(9)	C(4B)-C(5B)	1.528(9)
C(5A)-C(22A)	1.516(7)	C(5B)-C(22B)	1.514(8)
C(5A)-C(6A)	1.538(8)	C(5B)-C(6B)	1.564(8)
C(6A)-C(7A)	1.562(8)	C(6B)-C(7B)	1.555(8)
C(7A)-C(8A)	1.524(8)	C(7B)-C(8B)	1.544(8)
C(8A)-C(9A)	1.524(8)	C(8B)-C(9B)	1.533(8)
C(9A)-C(10A)	1.525(8)	C(9B)-C(10B)	1.526(9)
C(9A)-C(18A)	1.539(8)	C(9B)-C(18B)	1.543(8)
C(10A)-C(11A)	1.532(8)	C(10B)-C(11B)	1.538(9)
C(11A)-C(12A)	1.503(9)	C(11B)-C(12B)	1.497(9)
C(12A)-C(13A)	1.346(9)	C(12B)-C(13B)	1.343(9)
C(12A)-C(17A)	1.521(8)	C(12B)-C(17B)	1.521(9)
C(13A)-C(14A)	1.463(9)	C(13B)-C(14B)	1.468(9)
C(14A)-C(15A)	1.488(10)	C(14B)-C(15B)	1.486(10)
C(15A)-C(16A)	1.531(8)	C(15B)-C(16B)	1.522(9)
C(16A)-C(17A)	1.526(8)	C(16B)-C(17B)	1.529(9)
C(17A)-C(28A)	1.546(8)	C(17B)-C(28B)	1.529(9)
C(17A)-C(18A)	1.566(7)	C(17B)-C(18B)	1.567(8)
C(18A)-C(19A)	1.541(8)	C(18B)-C(19B)	1.528(8)
C(19A)-C(20A)	1.543(9)	C(19B)-C(20B)	1.528(9)
C(22A)-C(27A)	1.393(8)	C(22B)-C(23B)	1.381(9)
C(22A)-C(23A)	1.401(8)	C(22B)-C(27B)	1.402(8)
C(23A)-C(24A)	1.391(8)	C(23B)-C(24B)	1.402(9)
C(24A)-C(25A)	1.389(9)	C(24B)-C(25B)	1.392(9)
C(25A)-C(26A)	1.368(9)	C(25B)-C(26B)	1.360(9)
C(26A)-C(27A)	1.386(8)	C(26B)-C(27B)	1.387(8)

Table S4. Bond angles in **2b**, °.

C(20A)-C(1A)-C(21A)	112.0(6)	C(20B)-C(1B)-C(21B)	110.5(5)
C(20A)-C(1A)-C(8A)	107.0(5)	C(20B)-C(1B)-C(8B)	109.6(5)
C(21A)-C(1A)-C(8A)	112.2(6)	C(21B)-C(1B)-C(8B)	111.5(5)
C(20A)-C(1A)-C(2A)	117.7(6)	C(20B)-C(1B)-C(2B)	116.4(5)
C(21A)-C(1A)-C(2A)	108.6(5)	C(21B)-C(1B)-C(2B)	109.0(5)
C(8A)-C(1A)-C(2A)	98.7(5)	C(8B)-C(1B)-C(2B)	99.3(5)
C(3A)-C(2A)-C(1A)	118.4(5)	C(3B)-C(2B)-C(1B)	120.1(5)
C(3A)-C(2A)-C(6A)	103.7(5)	C(3B)-C(2B)-C(6B)	105.4(5)
C(1A)-C(2A)-C(6A)	105.4(5)	C(1B)-C(2B)-C(6B)	105.6(5)
C(3A)-C(2A)-Cl(1A)	104.2(4)	C(3B)-C(2B)-Cl(1B)	101.1(4)
C(1A)-C(2A)-Cl(1A)	112.3(4)	C(1B)-C(2B)-Cl(1B)	111.7(4)
C(6A)-C(2A)-Cl(1A)	112.8(4)	C(6B)-C(2B)-Cl(1B)	113.1(4)
O(1A)-C(3A)-C(4A)	125.2(7)	O(1B)-C(3B)-C(4B)	126.1(6)
O(1A)-C(3A)-C(2A)	126.0(7)	O(1B)-C(3B)-C(2B)	126.9(6)
C(4A)-C(3A)-C(2A)	108.8(6)	C(4B)-C(3B)-C(2B)	107.1(5)
C(3A)-C(4A)-C(5A)	105.8(5)	C(3B)-C(4B)-C(5B)	104.4(5)
C(22A)-C(5A)-C(4A)	116.4(5)	C(22B)-C(5B)-C(4B)	116.6(5)
C(22A)-C(5A)-C(6A)	111.7(5)	C(22B)-C(5B)-C(6B)	113.1(5)
C(4A)-C(5A)-C(6A)	103.0(5)	C(4B)-C(5B)-C(6B)	105.8(5)
C(5A)-C(6A)-C(2A)	106.3(5)	C(7B)-C(6B)-C(5B)	112.9(5)
C(5A)-C(6A)-C(7A)	114.0(5)	C(7B)-C(6B)-C(2B)	105.6(5)
C(2A)-C(6A)-C(7A)	102.9(5)	C(5B)-C(6B)-C(2B)	105.5(5)
C(8A)-C(7A)-C(6A)	106.2(5)	C(8B)-C(7B)-C(6B)	102.3(5)
C(7A)-C(8A)-C(9A)	117.8(5)	C(9B)-C(8B)-C(1B)	113.6(5)
C(7A)-C(8A)-C(1A)	105.6(5)	C(9B)-C(8B)-C(7B)	119.8(5)
C(9A)-C(8A)-C(1A)	112.2(5)	C(1B)-C(8B)-C(7B)	103.7(5)
C(8A)-C(9A)-C(10A)	111.5(5)	C(10B)-C(9B)-C(8B)	112.4(5)
C(8A)-C(9A)-C(18A)	109.4(5)	C(10B)-C(9B)-C(18B)	109.8(5)
C(10A)-C(9A)-C(18A)	110.1(5)	C(8B)-C(9B)-C(18B)	108.6(5)
C(9A)-C(10A)-C(11A)	110.6(5)	C(9B)-C(10B)-C(11B)	110.8(5)
C(12A)-C(11A)-C(10A)	112.4(5)	C(12B)-C(11B)-C(10B)	112.8(5)
C(13A)-C(12A)-C(11A)	120.3(6)	C(13B)-C(12B)-C(11B)	120.2(6)
C(13A)-C(12A)-C(17A)	122.8(5)	C(13B)-C(12B)-C(17B)	122.1(6)
C(11A)-C(12A)-C(17A)	116.9(5)	C(11B)-C(12B)-C(17B)	117.6(5)
C(12A)-C(13A)-C(14A)	123.0(6)	C(12B)-C(13B)-C(14B)	124.1(6)
O(2A)-C(14A)-C(13A)	120.9(7)	O(2B)-C(14B)-C(13B)	122.0(6)
O(2A)-C(14A)-C(15A)	122.8(7)	O(2B)-C(14B)-C(15B)	122.4(6)
C(13A)-C(14A)-C(15A)	116.2(5)	C(13B)-C(14B)-C(15B)	115.5(6)
C(14A)-C(15A)-C(16A)	110.1(6)	C(14B)-C(15B)-C(16B)	113.1(6)
C(17A)-C(16A)-C(15A)	113.1(5)	C(15B)-C(16B)-C(17B)	112.3(6)
C(12A)-C(17A)-C(16A)	110.0(5)	C(12B)-C(17B)-C(28B)	107.9(5)
C(12A)-C(17A)-C(28A)	108.5(5)	C(12B)-C(17B)-C(16B)	108.9(5)
C(16A)-C(17A)-C(28A)	110.0(5)	C(28B)-C(17B)-C(16B)	111.5(5)
C(12A)-C(17A)-C(18A)	107.5(5)	C(12B)-C(17B)-C(18B)	109.8(5)
C(16A)-C(17A)-C(18A)	109.2(5)	C(28B)-C(17B)-C(18B)	110.7(5)
C(28A)-C(17A)-C(18A)	111.7(5)	C(16B)-C(17B)-C(18B)	108.1(5)

C(9A)-C(18A)-C(19A)	113.6(5)	C(19B)-C(18B)-C(9B)	111.3(5)
C(9A)-C(18A)-C(17A)	113.0(5)	C(19B)-C(18B)-C(17B)	111.3(5)
C(19A)-C(18A)-C(17A)	112.9(5)	C(9B)-C(18B)-C(17B)	115.3(5)
C(18A)-C(19A)-C(20A)	113.0(5)	C(18B)-C(19B)-C(20B)	111.6(5)
C(1A)-C(20A)-C(19A)	110.6(6)	C(1B)-C(20B)-C(19B)	109.4(5)
C(27A)-C(22A)-C(23A)	118.2(5)	C(23B)-C(22B)-C(27B)	117.6(6)
C(27A)-C(22A)-C(5A)	124.1(6)	C(23B)-C(22B)-C(5B)	120.0(5)
C(23A)-C(22A)-C(5A)	117.6(5)	C(27B)-C(22B)-C(5B)	122.4(6)
C(24A)-C(23A)-C(22A)	120.7(6)	C(22B)-C(23B)-C(24B)	122.4(6)
C(25A)-C(24A)-C(23A)	118.4(6)	C(25B)-C(24B)-C(23B)	117.3(6)
C(26A)-C(25A)-C(24A)	122.4(6)	C(26B)-C(25B)-C(24B)	122.0(6)
C(26A)-C(25A)-Cl(2A)	119.7(5)	C(26B)-C(25B)-Cl(2B)	119.4(5)
C(24A)-C(25A)-Cl(2A)	117.9(5)	C(24B)-C(25B)-Cl(2B)	118.7(5)
C(25A)-C(26A)-C(27A)	118.4(6)	C(25B)-C(26B)-C(27B)	119.7(6)
C(26A)-C(27A)-C(22A)	121.7(6)	C(26B)-C(27B)-C(22B)	121.0(6)

Table S5. Bond lengths in **2g**, Å.

Cl(1)-C(2)	1.8053(17)	C(9)-C(18)	1.541(2)
Cl(2)-C(23)	1.739(2)	C(10)-C(11)	1.522(2)
Cl(3)-C(25)	1.738(2)	C(11)-C(12)	1.502(2)
O(1)-C(3)	1.201(2)	C(12)-C(13)	1.348(2)
O(2)-C(14)	1.220(2)	C(12)-C(17)	1.528(2)
C(1)-C(20)	1.525(2)	C(13)-C(14)	1.465(3)
C(1)-C(8)	1.544(2)	C(14)-C(15)	1.499(3)
C(1)-C(21)	1.544(2)	C(15)-C(16)	1.523(3)
C(1)-C(2)	1.552(2)	C(16)-C(17)	1.536(2)
C(2)-C(3)	1.549(2)	C(17)-C(28)	1.543(2)
C(2)-C(6)	1.551(2)	C(17)-C(18)	1.560(2)
C(3)-C(4)	1.514(3)	C(18)-C(19)	1.546(2)
C(4)-C(5)	1.531(3)	C(19)-C(20)	1.541(3)
C(5)-C(22)	1.510(3)	C(22)-C(27)	1.396(3)
C(5)-C(6)	1.560(2)	C(22)-C(23)	1.402(3)
C(6)-C(7)	1.549(2)	C(23)-C(24)	1.381(3)
C(7)-C(8)	1.528(2)	C(24)-C(25)	1.386(3)
C(8)-C(9)	1.518(2)	C(25)-C(26)	1.373(3)
C(9)-C(10)	1.525(2)	C(26)-C(27)	1.393(3)

Table S6. Bond angles in **2g**, °.

C(20)-C(1)-C(8)	108.34(14)	C(13)-C(12)-C(17)	121.85(15)
C(20)-C(1)-C(21)	110.94(15)	C(11)-C(12)-C(17)	118.19(15)
C(8)-C(1)-C(21)	112.12(14)	C(12)-C(13)-C(14)	123.75(16)
C(20)-C(1)-C(2)	118.10(14)	O(2)-C(14)-C(13)	121.33(17)
C(8)-C(1)-C(2)	98.77(12)	O(2)-C(14)-C(15)	121.74(18)

C(21)-C(1)-C(2)	108.05(15)	C(13)-C(14)-C(15)	116.87(16)
C(3)-C(2)-C(6)	105.11(14)	C(14)-C(15)-C(16)	112.98(16)
C(3)-C(2)-C(1)	116.38(14)	C(15)-C(16)-C(17)	113.20(14)
C(6)-C(2)-C(1)	105.65(13)	C(12)-C(17)-C(16)	108.55(14)
C(3)-C(2)-Cl(1)	103.63(11)	C(12)-C(17)-C(28)	107.61(13)
C(6)-C(2)-Cl(1)	112.42(12)	C(16)-C(17)-C(28)	109.77(14)
C(1)-C(2)-Cl(1)	113.53(12)	C(12)-C(17)-C(18)	110.58(13)
O(1)-C(3)-C(4)	125.84(17)	C(16)-C(17)-C(18)	109.01(13)
O(1)-C(3)-C(2)	125.42(16)	C(28)-C(17)-C(18)	111.28(14)
C(4)-C(3)-C(2)	108.71(15)	C(9)-C(18)-C(19)	111.99(14)
C(3)-C(4)-C(5)	106.11(15)	C(9)-C(18)-C(17)	112.65(13)
C(22)-C(5)-C(4)	115.72(16)	C(19)-C(18)-C(17)	112.68(13)
C(22)-C(5)-C(6)	111.85(15)	C(20)-C(19)-C(18)	113.31(14)
C(4)-C(5)-C(6)	104.33(14)	C(1)-C(20)-C(19)	110.23(14)
C(7)-C(6)-C(2)	104.91(13)	C(27)-C(22)-C(23)	116.19(17)
C(7)-C(6)-C(5)	112.56(14)	C(27)-C(22)-C(5)	123.98(17)
C(2)-C(6)-C(5)	106.51(14)	C(23)-C(22)-C(5)	119.83(16)
C(8)-C(7)-C(6)	104.83(13)	C(24)-C(23)-C(22)	123.17(18)
C(9)-C(8)-C(7)	117.80(13)	C(24)-C(23)-Cl(2)	117.60(15)
C(9)-C(8)-C(1)	113.61(13)	C(22)-C(23)-Cl(2)	119.22(15)
C(7)-C(8)-C(1)	104.28(13)	C(23)-C(24)-C(25)	118.08(19)
C(8)-C(9)-C(10)	111.00(13)	C(26)-C(25)-C(24)	121.46(19)
C(8)-C(9)-C(18)	109.43(13)	C(26)-C(25)-Cl(3)	120.24(17)
C(10)-C(9)-C(18)	110.00(14)	C(24)-C(25)-Cl(3)	118.29(17)
C(11)-C(10)-C(9)	110.62(14)	C(25)-C(26)-C(27)	119.1(2)
C(12)-C(11)-C(10)	113.34(14)	C(26)-C(27)-C(22)	121.92(19)
C(13)-C(12)-C(11)	119.73(15)		

Table S7. Intramolecular non-valence interactions (Å) within two neighboring 5-membered ring and the phenyl group in **2b** and **2g.**

Conformation **2'b**		Conformation **2"b**		Molecule **2'g**	
Cl1A···O1A	3.115(6)	Cl1B···O1B	3.252(7)	Cl1···O1	3.0523(15)
Cl1A···H6A	2.807	Cl1B···H6B	2.754	Cl1···H6	2.750
Cl1A···H21B	2.689	Cl1B···H21E	2.745	Cl1···H21B	2.800
O1A···H20A	2.549	O1B···H20C	2.339	O1···H20A	2.560
H4B···H5A	2.234	H4D···H5B	2.258	H4B···H5	2.229
H4B···H8A	2.786	-	-	H4B···H8	2.505
-	-	H5B···H8B	2.596	-	-
H5A···H7A	2.309	H5B···H7C	2.276	H5···H7A	2.193
H6A···H7B	2.269	H6B···H7D	2.294	H6···H7B	2.261
H6A···H21C	2.266	H6B···H21F	2.661	H6···H21C	2.422
H5A···H23A	2.462	H5B···H23B	2.487	H5···Cl2	2.654
H6A···H23A	2.751	-	-	H6···Cl2	2.984
-	-	H7C···H23B	2.416	-	-
H4A···H27A	2.195	H4C···H27B	2.027	H4A···H27	2.176

One of the reasons for the absence of conformer **2"g** (similar in structure to **2"b**) in the crystal lattice of **2g** may be its significantly lower stability compared to **2'g**. Either a very short distance C4-H4A···Cl2 or short contacts C5-H5···Cl2 and C6-H6···Cl2 should be present in **2"g**, which significantly increases the intramolecular Van der Waals repulsion, but the rotation of the phenyl group about the C5-C22 bond (with a deviation of the C4-C5-C22-C27 torsion angle from its optimal value of 15.7-16.7°) cannot provide a decrease in the total intramolecular repulsion due to the appearance of additional Van der Waals interactions between the hydrogens of the terminal five-membered ring and the *ortho*-hydrohen atoms of the phenyl group.

IV.1. DFT calculations

The geometries of two conformers of **2b**, established by X-ray diffraction, were optimized with GAUSSIAN09 [S11] using the ωB97X-D functional [S12] and the 6-31+G(d,p) basis set both in the gas phase and in chloroform (SMD-PCM continuum solvation model). Calculation of vibrational frequencies was performed to prove that the optimized structure corresponds to a true minimum on the potential energy surface. The X-ray atomic coordinates were taken as the starting coordinates. The energies of the conformers, which were optimized in vacuum and in solution, and those determined by X-ray diffraction were calculated at the ωB97X-D/6-311++G(d,p) level of theory..

Cartesian coordinate columns of the optimized structure of compound **2'b** (first conformer) (*ω*B97XD/6-31+G(d,p)):

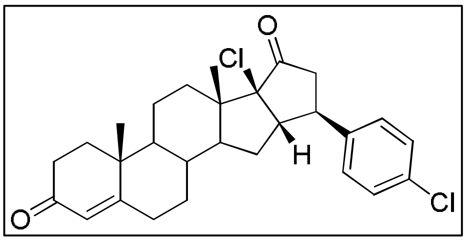

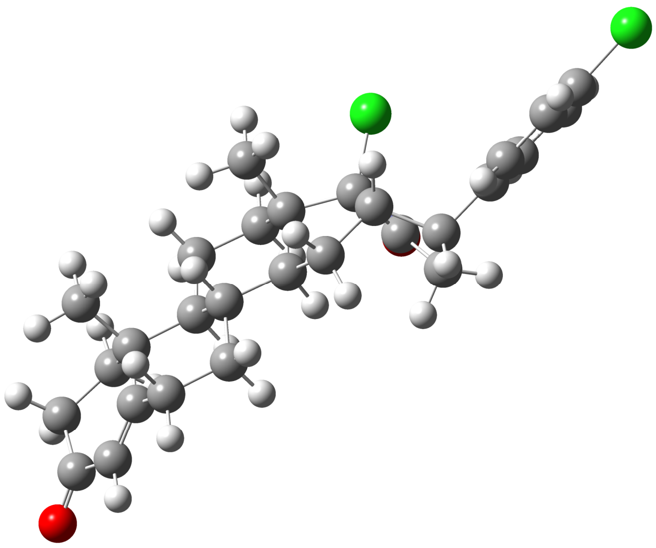

1	Cl	-2.4104070	2.0781270	-0.6288300
2	Cl	-8.4797960	-0.1448600	-0.2199410
3	O	-1.1938760	1.6259560	2.2359430
4	O	8.1346720	-0.8557190	1.5395140
5	C	0.1661310	0.9562650	-0.5789010
6	C	-1.2894760	0.7754440	-0.0678200
7	C	-1.4358210	0.7193210	1.4784180
8	C	-1.9401150	-0.6556640	1.8810830
9	H	-2.5891080	-0.5996790	2.7570070
10	H	-1.0594310	-1.2440000	2.1696160
11	C	-2.5771140	-1.2473130	0.6201790
12	H	-2.4632840	-2.3358770	0.6084590
13	C	-1.7169050	-0.6467660	-0.5310420
14	H	-2.2981270	-0.6046670	-1.4537740
15	C	-0.3741730	-1.4027440	-0.7549830
16	H	-0.3598040	-2.3631130	-0.2302910

17	H	-0.2299640	-1.6180410	-1.8191630
18	C	0.7149260	-0.4473520	-0.2512610
19	H	0.7433480	-0.5236410	0.8484740
20	C	2.1555840	-0.6656170	-0.7057510
21	H	2.1998200	-0.5728580	-1.7998240
22	C	2.6474840	-2.0633010	-0.3333380
23	H	2.0340010	-2.8275720	-0.8230030
24	H	2.5360120	-2.2087120	0.7504810
25	C	4.1116520	-2.2567460	-0.7252830
26	H	4.4756480	-3.2371250	-0.4047350
27	H	4.1886640	-2.2368260	-1.8216770
28	C	5.0005030	-1.1755010	-0.1694940
29	C	6.1338560	-1.5012860	0.4778910
30	H	6.3906910	-2.5428880	0.6555360
31	C	7.1353960	-0.5182590	0.9259640
32	C	6.8791920	0.9151990	0.5202470
33	H	7.3389480	1.0570650	-0.4670770
34	H	7.4072770	1.5752130	1.2126030
35	C	5.3856310	1.2265160	0.4729910
36	H	4.9857650	1.1728980	1.4939900
37	H	5.2451680	2.2570840	0.1338010
38	C	4.5691260	0.2683430	-0.4227320
39	C	3.0541920	0.4131940	-0.0564340
40	H	2.9957580	0.2275040	1.0293260
41	C	2.5102220	1.8370690	-0.2890290
42	H	2.6333140	2.1153200	-1.3411910
43	H	3.1012750	2.5541200	0.2872950
44	C	1.0394800	1.9981970	0.1241260
45	H	0.9579010	1.8806310	1.2086190
46	H	0.6924510	3.0112190	-0.1056760
47	C	0.1341880	1.2304130	-2.0955450
48	H	1.1214960	1.1109560	-2.5448460
49	H	-0.1929470	2.2557010	-2.2822060
50	H	-0.5513650	0.5665690	-2.6283720
51	C	-4.0579460	-0.9496490	0.4611770
52	C	-4.8170980	-1.7613070	-0.3876720
53	H	-4.3427830	-2.6013040	-0.8896560
54	C	-6.1697070	-1.5255710	-0.6046000
55	H	-6.7471700	-2.1665010	-1.2614030
56	C	-6.7792870	-0.4546990	0.0412840
57	C	-6.0536480	0.3674900	0.8933840
58	H	-6.5368990	1.2017000	1.3893980
59	C	-4.7001100	0.1133350	1.0986880
60	H	-4.1525400	0.7769000	1.7602210
61	C	4.8338490	0.5936270	-1.9109180

62	H	4.6382050	1.6491050	-2.1188260
63	H	4.2080170	-0.0017580	-2.5812560
64	H	5.8766690	0.3893210	-2.1701640

Cartesian coordinate columns of the optimized structure of compound **2''b** (second conformer) (ωB97XD/6-31+G(d,p)):

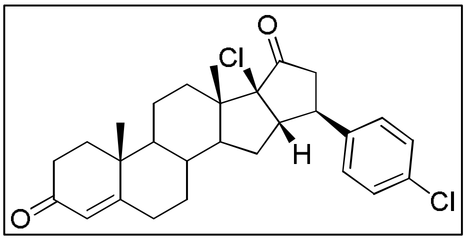

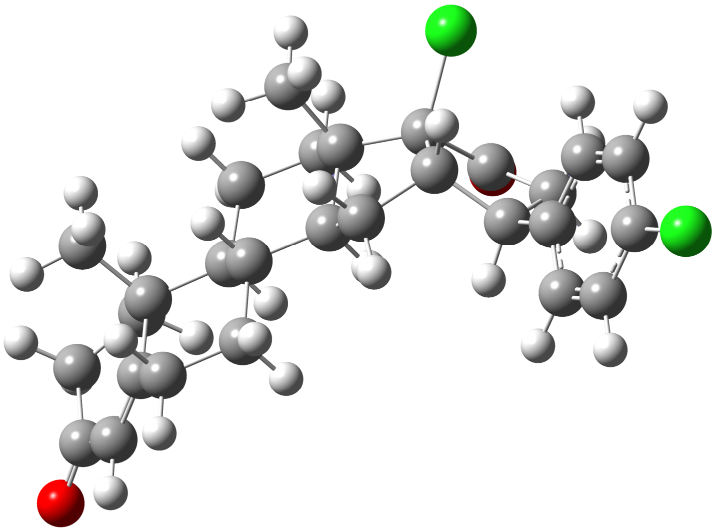

1	Cl	-1.8584070	3.4432550	-0.5417910
2	Cl	-7.9615920	-2.3806010	-0.4152970
3	O	-0.6300950	2.5477270	2.3864210
4	O	7.4390430	-2.5317870	1.4289130
5	C	0.3908520	1.7174250	-0.4676450
6	C	-1.0615040	1.9079460	0.0444570
7	C	-1.3229280	1.9907060	1.5715620
8	C	-2.6070520	1.2419530	1.8547570
9	H	-3.4407780	1.9264380	1.6542830
10	H	-2.6674650	0.9301510	2.8983520
11	C	-2.5817100	0.0874510	0.8460020
12	H	-1.9470130	-0.6982480	1.2751350
13	C	-1.8468290	0.6405230	-0.4155380
14	H	-2.5759570	0.8989890	-1.1851530
15	C	-0.7823400	-0.3456870	-0.9421210
16	H	-1.0183750	-1.3750620	-0.6563330
17	H	-0.7132650	-0.3231430	-2.0341950
18	C	0.5090900	0.1817750	-0.3115860
19	H	0.4543220	-0.0263680	0.7719390
20	C	1.8444970	-0.3899800	-0.7835860
21	H	1.9442820	-0.2060590	-1.8626760
22	C	1.9082080	-1.9007600	-0.5626040
23	H	1.1127820	-2.4037890	-1.1229670
24	H	1.7393850	-2.1181380	0.5014920
25	C	3.2644870	-2.4608280	-0.9881520

26	H	3.3257200	-3.5343390	-0.7881550
27	H	3.3715830	-2.3366560	-2.0750650
28	C	4.4070950	-1.7487270	-0.3145450
29	C	5.3755010	-2.4551200	0.2960370
30	H	5.3159400	-3.5391450	0.3581630
31	C	6.6010620	-1.8571310	0.8529500
32	C	6.7815130	-0.3765930	0.6074990
33	H	7.2917150	-0.2693100	-0.3590570
34	H	7.4550000	0.0252870	1.3683280
35	C	5.4414280	0.3548080	0.5956500
36	H	5.6124490	1.4135680	0.3803500
37	H	5.0079910	0.3035540	1.6029130
38	C	4.4173630	-0.2237200	-0.4065500
39	C	2.9965110	0.3173870	-0.0330700
40	H	2.8548570	0.0637090	1.0308930
41	C	2.8880030	1.8501550	-0.1394990
42	H	3.0844270	2.1690040	-1.1689880
43	H	3.6586170	2.3193050	0.4786340
44	C	1.5236860	2.3805270	0.3237770
45	H	1.4037700	2.1744280	1.3896570
46	H	1.4828370	3.4693800	0.2112960
47	C	0.4670070	2.1491360	-1.9474940
48	H	1.3511660	1.7371390	-2.4366430
49	H	0.5274210	3.2379050	-2.0165740
50	H	-0.4061410	1.8360940	-2.5247810
51	C	-3.9268450	-0.5259410	0.5391930
52	C	-4.1707390	-1.8762460	0.7893710
53	H	-3.3863970	-2.4934760	1.2193360
54	C	-5.4045470	-2.4562170	0.5003260
55	H	-5.5820130	-3.5073560	0.6979990
56	C	-6.4085550	-1.6684690	-0.0474490
57	C	-6.1947300	-0.3174310	-0.3079680
58	H	-6.9874280	0.2861060	-0.7358340
59	C	-4.9570090	0.2418130	-0.0146570
60	H	-4.7945850	1.2961870	-0.2258960
61	C	4.8176510	0.1666320	-1.8477350
62	H	4.9628780	1.2468990	-1.9333350
63	H	4.0612160	-0.1276460	-2.5802420
64	H	5.7544810	-0.3204040	-2.1333480

IV.2. Transition state

Transition state of thermal switching between **2'b** and **2''b** TS (*ω*B97XD/6-311++G(d,p), QST2 formalism)

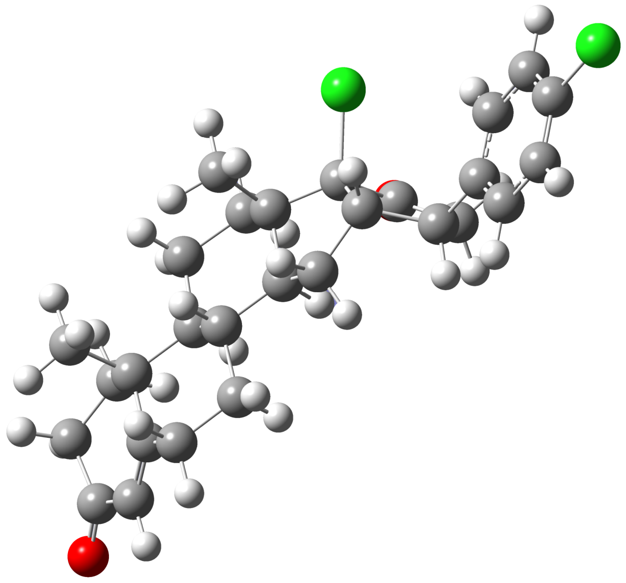

1	Cl	-2.1913100	2.5809670	-0.4972310
2	Cl	-8.5098170	-0.9825060	-0.5480180
3	O	-0.7333350	2.0393180	2.3287670
4	O	8.0581490	-1.3573720	1.3978540
5	C	0.2680480	1.2036070	-0.5112720
6	C	-1.1651240	1.1907080	0.0704540
7	C	-1.2984650	1.2609260	1.6124220
8	C	-2.2734190	0.2021540	2.0867170
9	H	-3.1378240	0.7071520	2.5258380
10	H	-1.8002730	-0.3545880	2.8989600
11	C	-2.6371230	-0.6869170	0.8864760
12	H	-2.3334580	-1.7119990	1.1072240
13	C	-1.7626830	-0.1879140	-0.3123430
14	H	-2.3843280	-0.1026780	-1.2025190
15	C	-0.5231940	-1.0803240	-0.6006830
16	H	-0.5834680	-2.0387150	-0.0811700
17	H	-0.4487010	-1.3002120	-1.6694460
18	C	0.6735780	-0.2401220	-0.1485490
19	H	0.7158860	-0.2835940	0.9523150
20	C	2.0635180	-0.6312650	-0.6379280
21	H	2.0695400	-0.5966270	-1.7350340
22	C	2.4191800	-2.0532110	-0.2117470
23	H	1.7054990	-2.7683070	-0.6316450
24	H	2.3452380	-2.1299610	0.8809060
25	C	3.8329400	-2.4239470	-0.6525240
26	H	4.1038480	-3.4214760	-0.2996590

27	H	3.8613350	-2.4601260	-1.7494950
28	C	4.8547540	-1.4229870	-0.1874510
29	C	5.9672380	-1.8362120	0.4372430
30	H	6.1176500	-2.8882560	0.6621380
31	C	7.0869840	-0.9490600	0.7956920
32	C	6.9722210	0.4764850	0.3090850
33	H	7.3938770	0.5043750	-0.7031870
34	H	7.6027610	1.1118460	0.9332800
35	C	5.5225760	0.9491770	0.3048020
36	H	5.1673280	0.9913740	1.3412280
37	H	5.4779560	1.9689320	-0.0845670
38	C	4.5699430	0.0425150	-0.5021050
39	C	3.0993440	0.3712570	-0.0841860
40	H	3.0704260	0.2493490	1.0102200
41	C	2.7032600	1.8291970	-0.3769510
42	H	2.7968710	2.0339250	-1.4474500
43	H	3.3977540	2.5065090	0.1246600
44	C	1.2851110	2.1694910	0.0995370
45	H	1.2503180	2.1077030	1.1880730
46	H	1.0376660	3.2025570	-0.1622640
47	C	0.1899040	1.4206150	-2.0347780
48	H	1.1291840	1.1601600	-2.5225620
49	H	-0.0144560	2.4706820	-2.2503200
50	H	-0.5998130	0.8317840	-2.5048750
51	C	-4.1147950	-0.7380140	0.5582640
52	C	-4.7201730	-1.9643230	0.2914790
53	H	-4.1343210	-2.8761790	0.3525500
54	C	-6.0647970	-2.0520090	-0.0486540
55	H	-6.5238700	-3.0119790	-0.2494410
56	C	-6.8162830	-0.8901430	-0.1229670
57	C	-6.2426130	0.3463570	0.1382380
58	H	-6.8407870	1.2468830	0.0761620
59	C	-4.8985550	0.4133700	0.4764740
60	H	-4.4589830	1.3862090	0.6655890
61	C	4.7992800	0.2609520	-2.0133450
62	H	4.7080240	1.3180270	-2.2720220
63	H	4.0854100	-0.2960020	-2.6238680
64	H	5.8004420	-0.0683460	-2.3017640

IV.3. Thermodynamic calculations

Thermodynamic calculations were performed at the ωB97xD 6-311++G(d,p) level of theory for previously optimized structures (section V.1.)

Compound **2'b**:

Sum of electronic and thermal Enthalpies= -2156.520192

Sum of electronic and thermal Free Energies= -2156.604506

Compound **2''b**:

Sum of electronic and thermal Enthalpies= -2156.519442

Sum of electronic and thermal Free Energies= -2156.604155

V. ^{1}H NMR monitoring

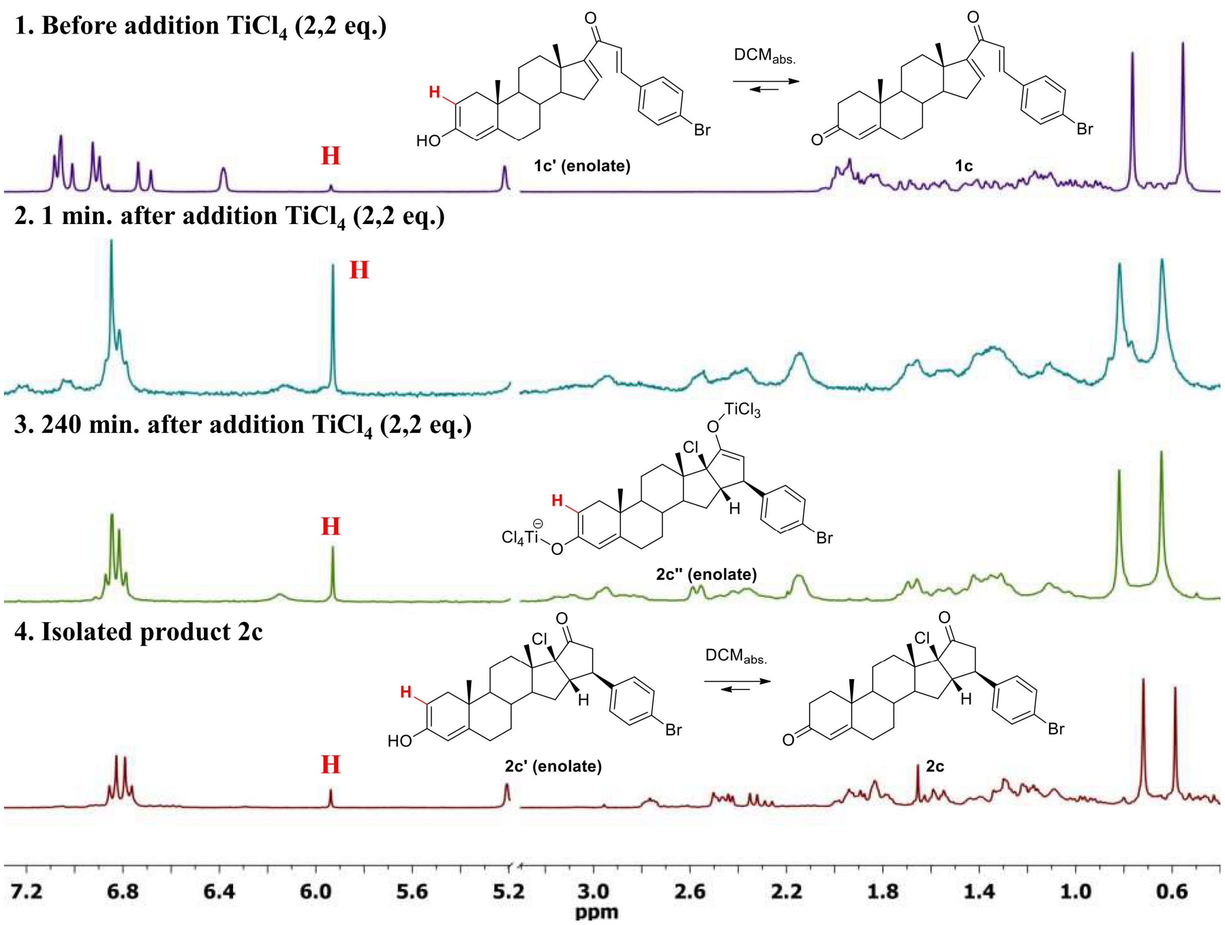

Figure S4. ^{1}H-NMR-monitoring of Nazarov cyclization of compound **1c** in CH_2Cl_2.

Signal (5.9 ppm) is estimated as CH of enolate and could be seen in CH_2Cl_2 (in $CDCl_3$ not obtained this signal). As evidence we could see that equilibrium without acid turned into ketone (integral of the signal above 0.1-0.2) and with addition of 2,2 eq. of $TiCl_4$ the equilibrium shifts to enolate. Moreover we suggest, that this signal estimates to cyclohexenone ring, not to cyclopentanone due presence it in starting spectra of benzylidene **1c**.

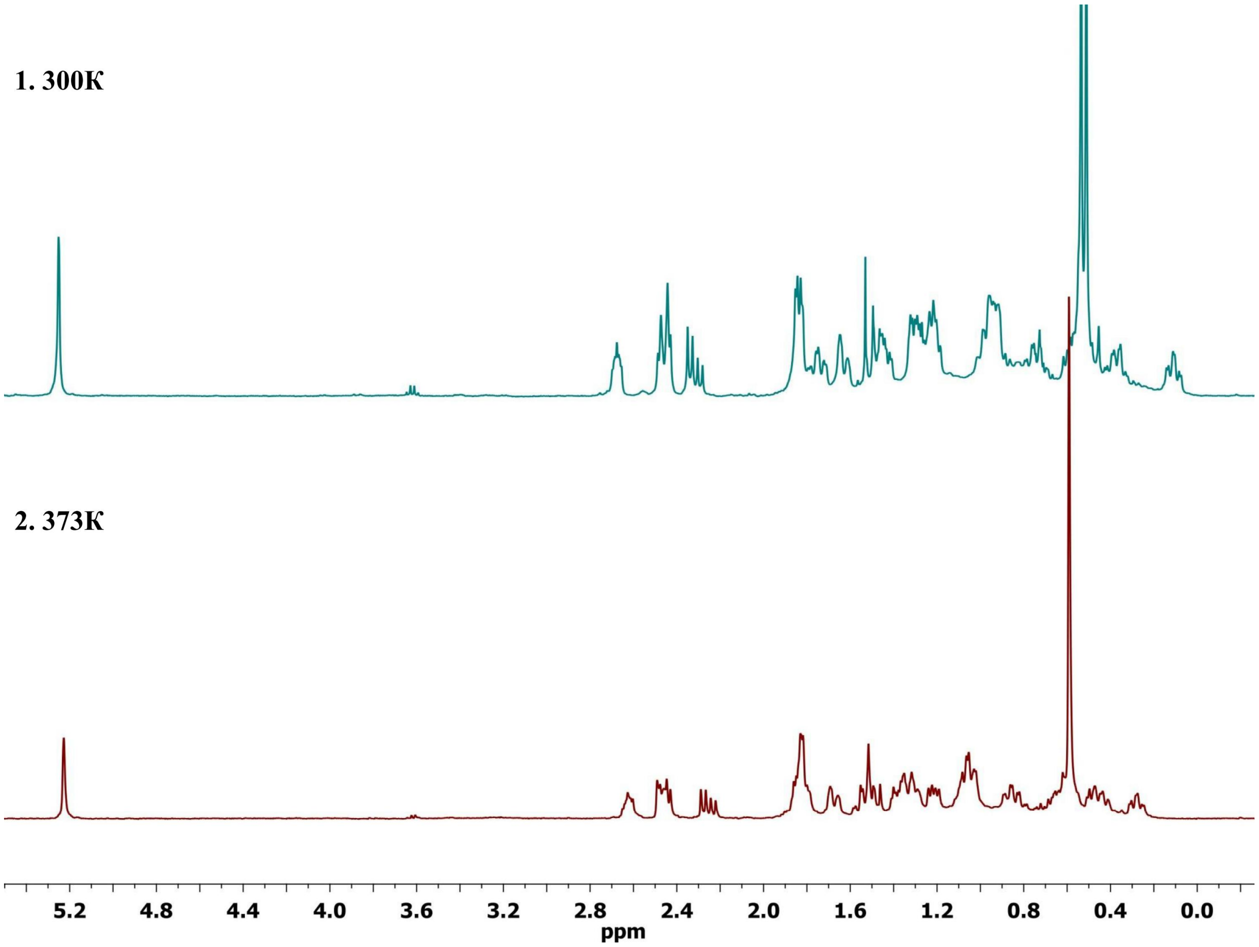

Figure S5. ^{1}H-NMR-monitoring of compound **2b** in 1,2-dichlorobenzene.

As you can see, the signal at ~ 2.4 ppm (at 300 K) does not disappear at 373 K, which is proof that this is not a set of two conformer doublets.

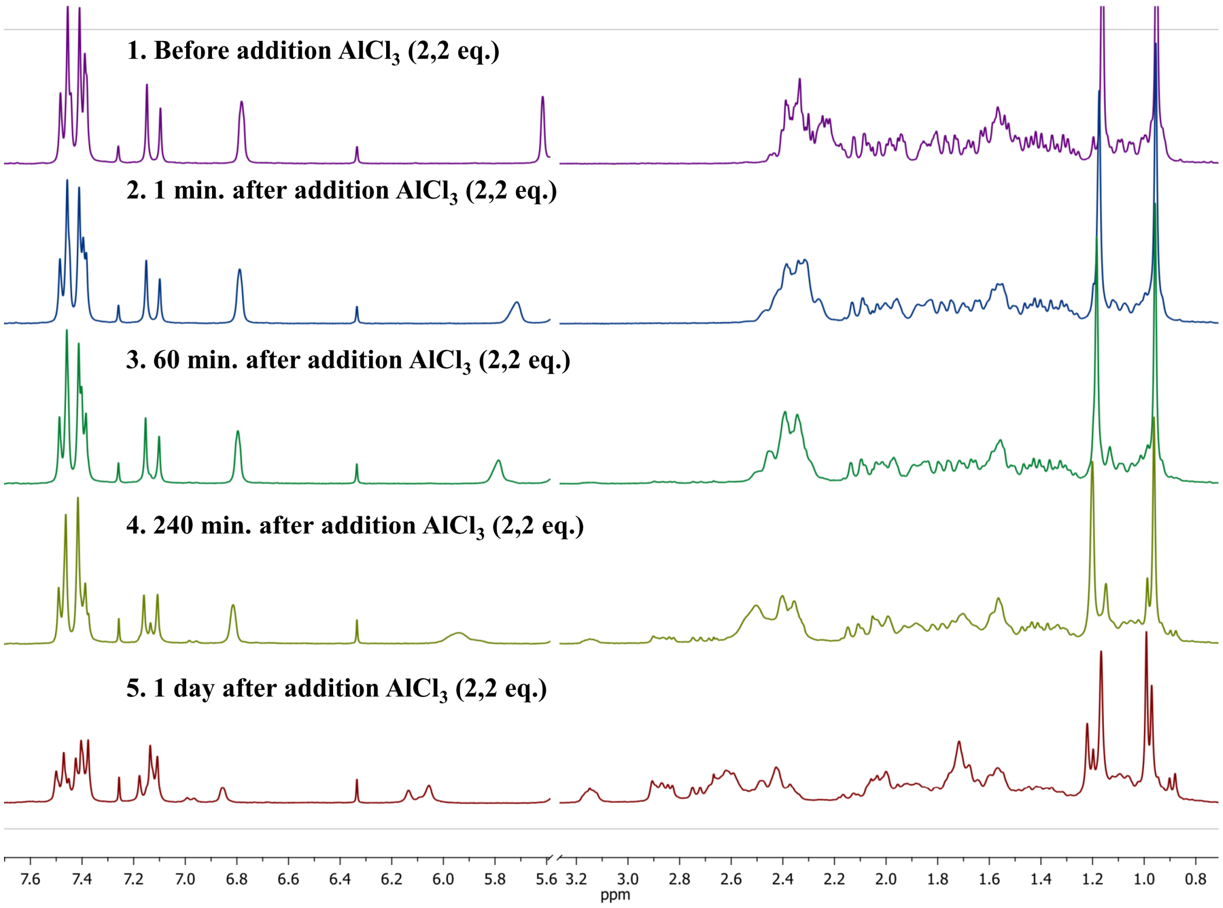

Figure S6. ^{1}H-NMR-monitoring of **1c** reaction with $AlCl_3$ in CH_2Cl_2

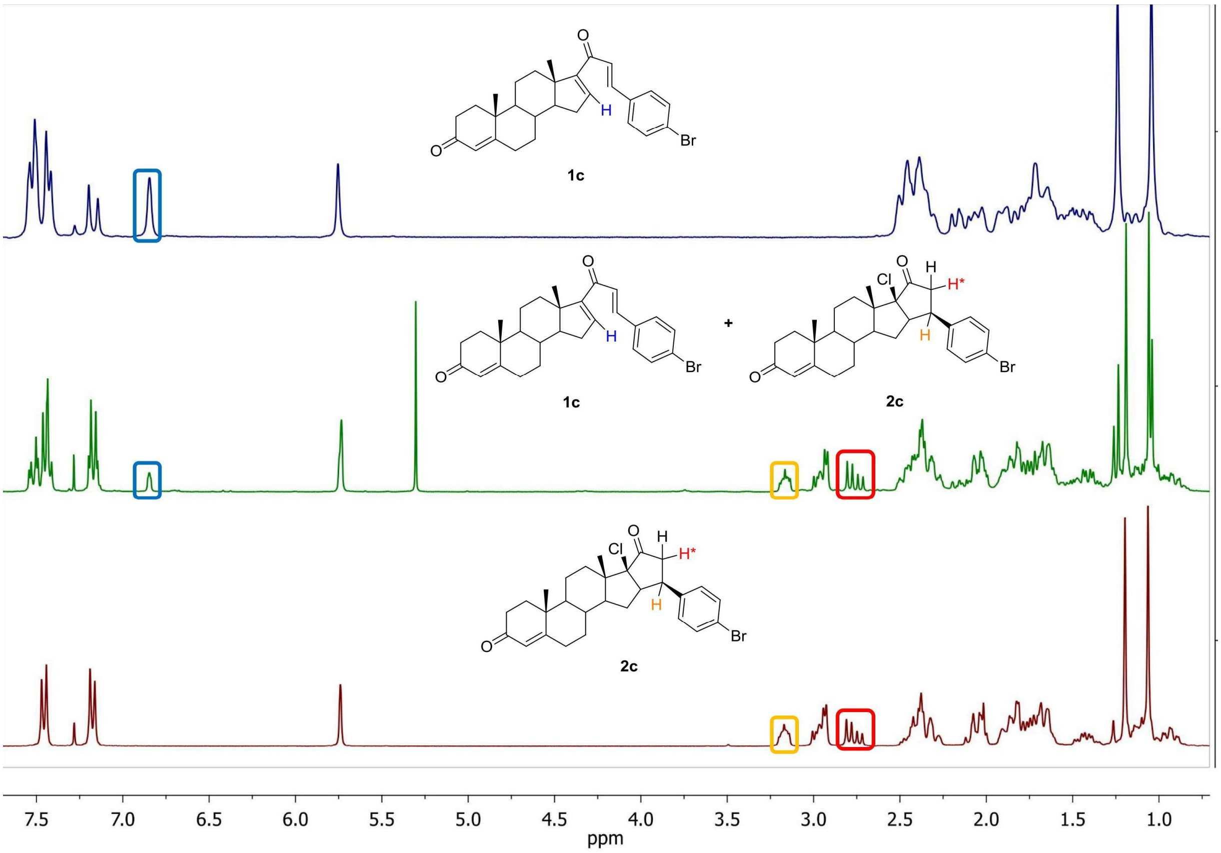

Figure S7. Comparison of ^{1}H NMR spectra of the compounds **1c**, **2c** and crude reaction mixture of compound **1c**.

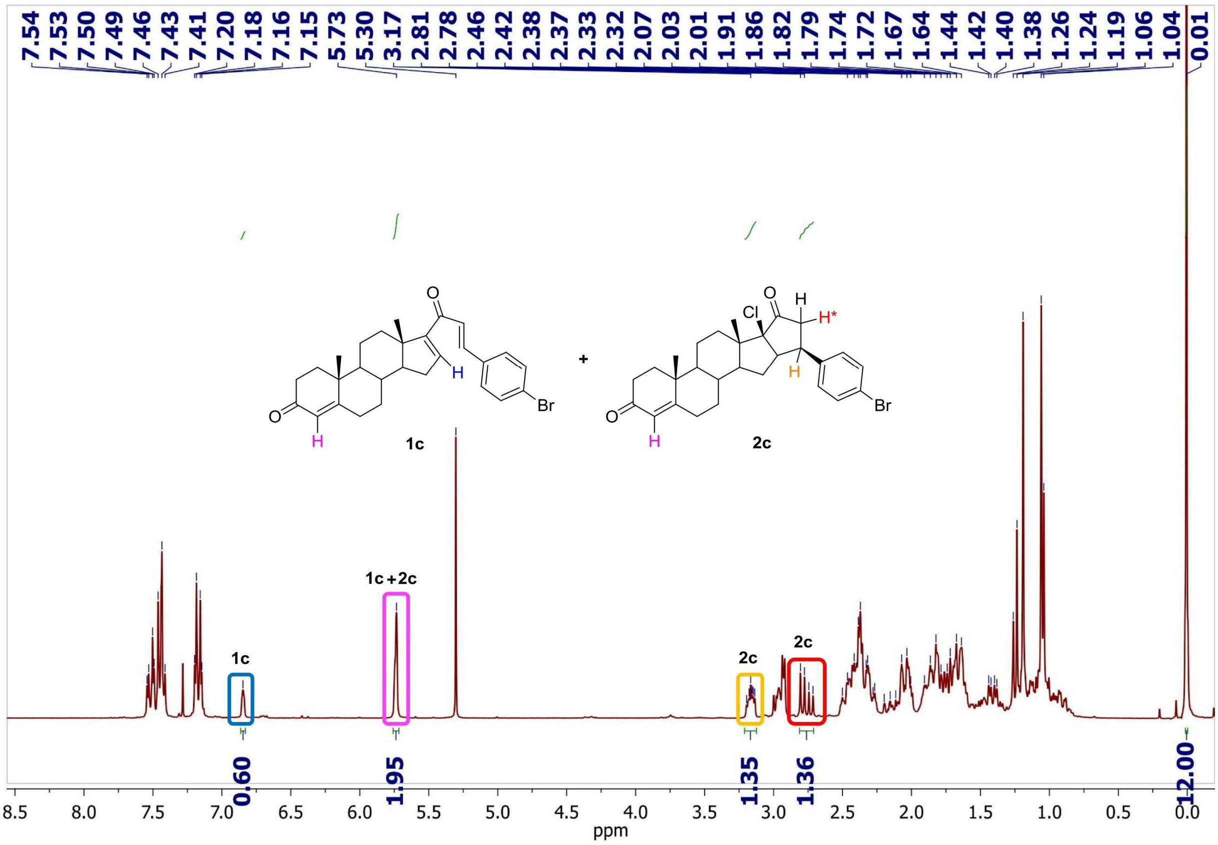

Figure S8. ^{1}H NMR spectra of the crude reaction mixture of compound **1c** with TMS.

We used TMS as internal standard (Note: TMS should be added just before the spectrum registration) to calculate the yield of products. As seen the spectra of crude reaction mixture had incomplete conversion, but any extra signals of byproducts or another diastereomer (the area from 2.5 to 3.2 ppm are clear from another compounds) are not observed. Moreover the sum of the integrals of single proton from cyclopentanone ring **2c** (red or orange marked) and divinylketone **1c** (blue marked) converges with the integral of single proton from ring **A** (violet marked) which refers for both compounds.

VI. Copies of ^{1}H and ^{13}C NMR spectra.

Compound 2a

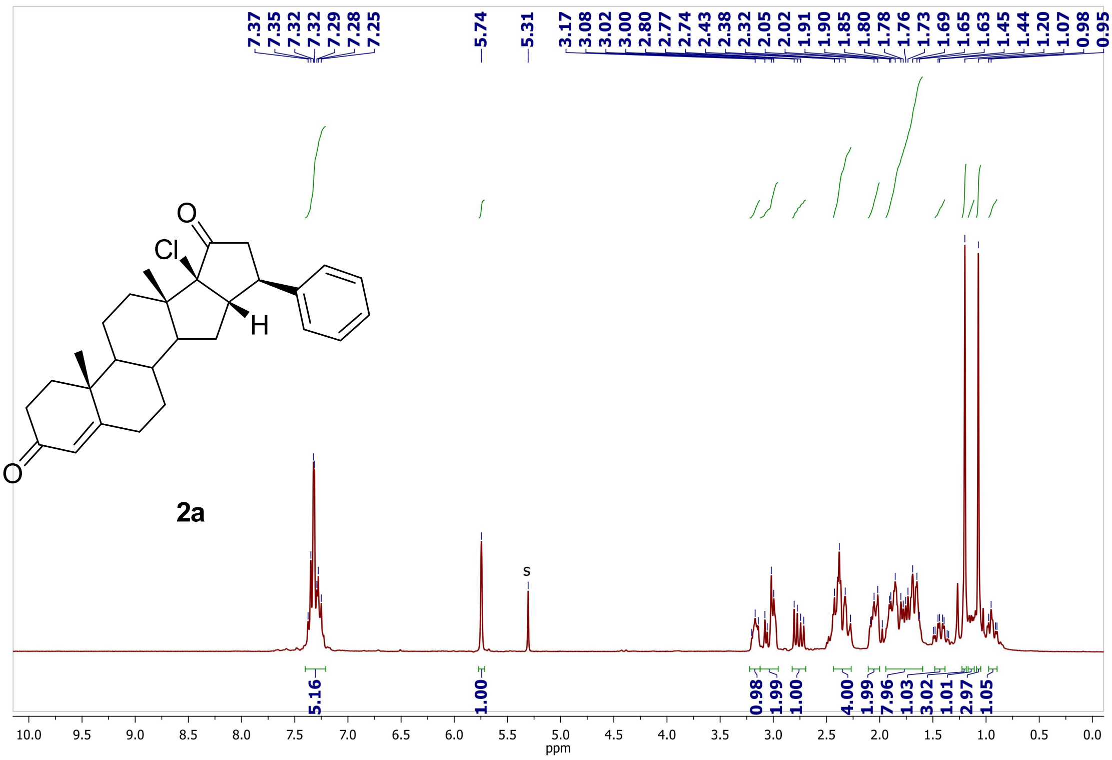

S - peak of CH_2Cl_2 (5.31 ppm).

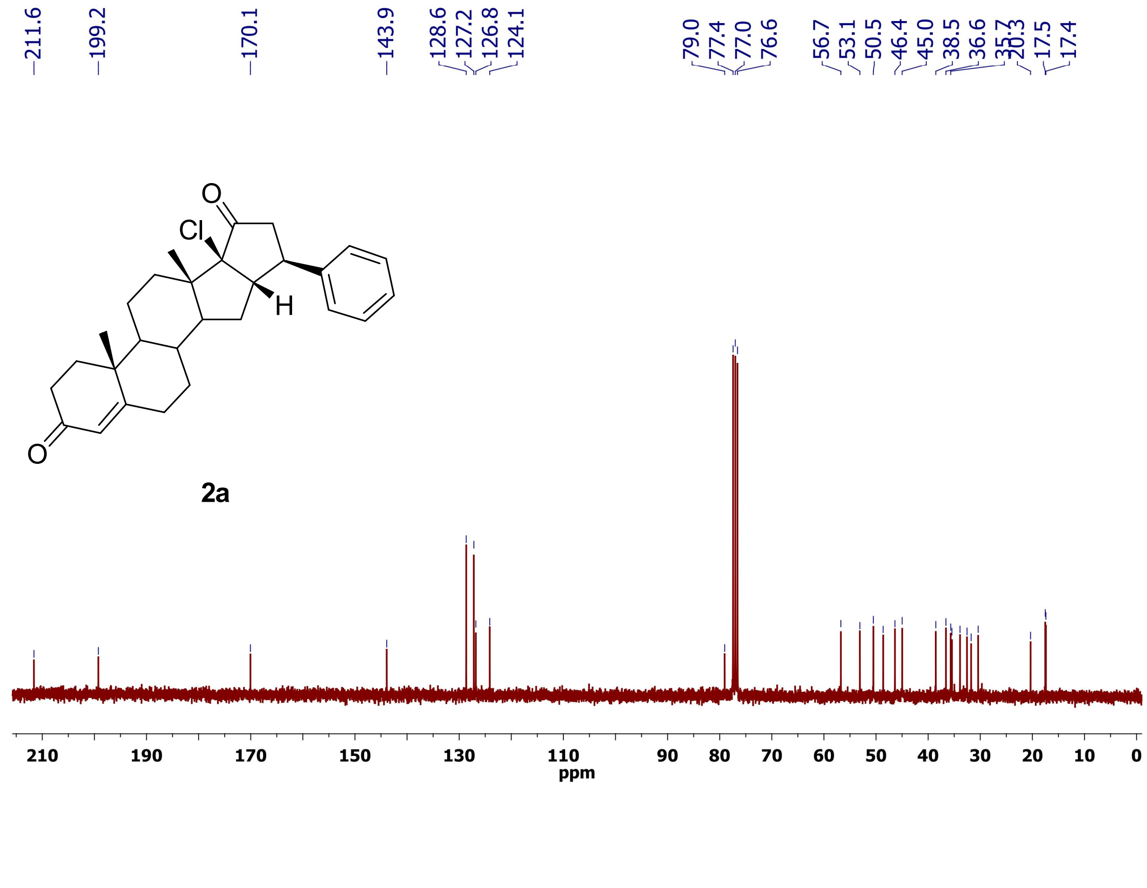

Compound 2b

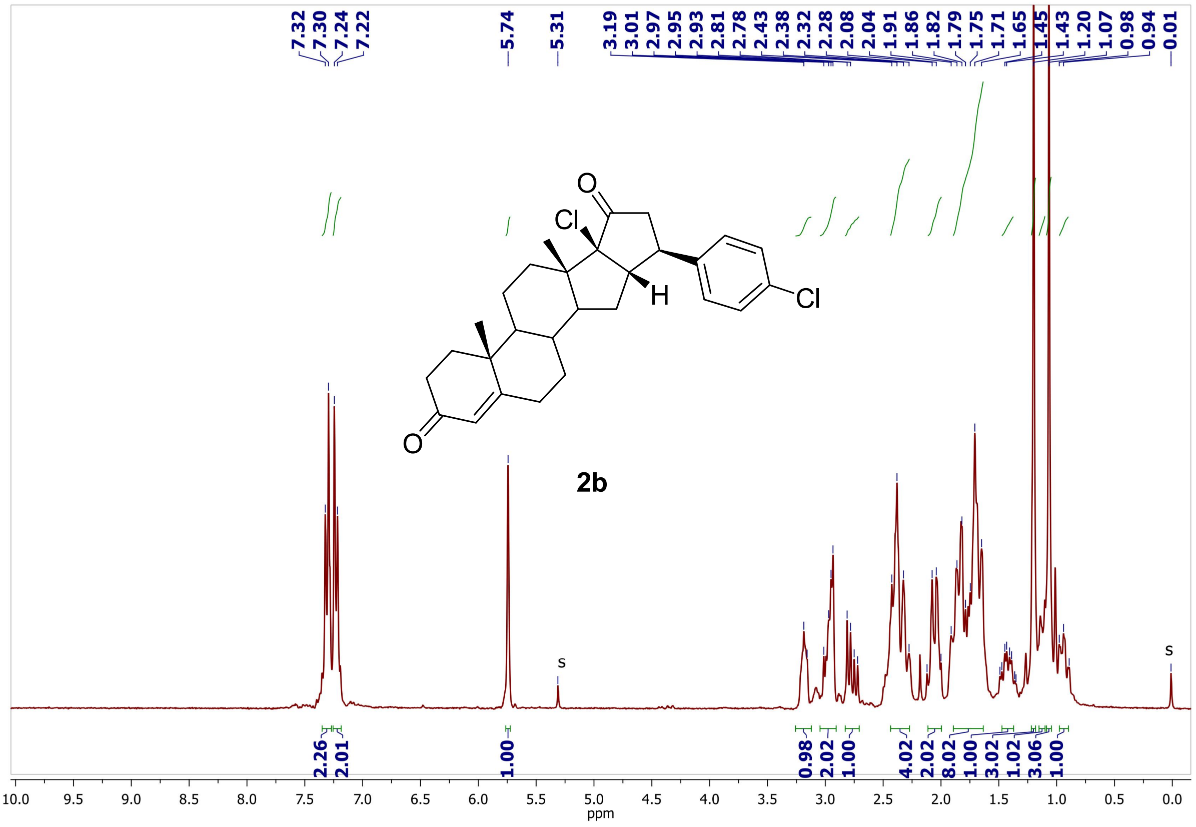

S - peaks of CH_2Cl_2 (5.31 ppm); silicone grease (0.01 ppm).

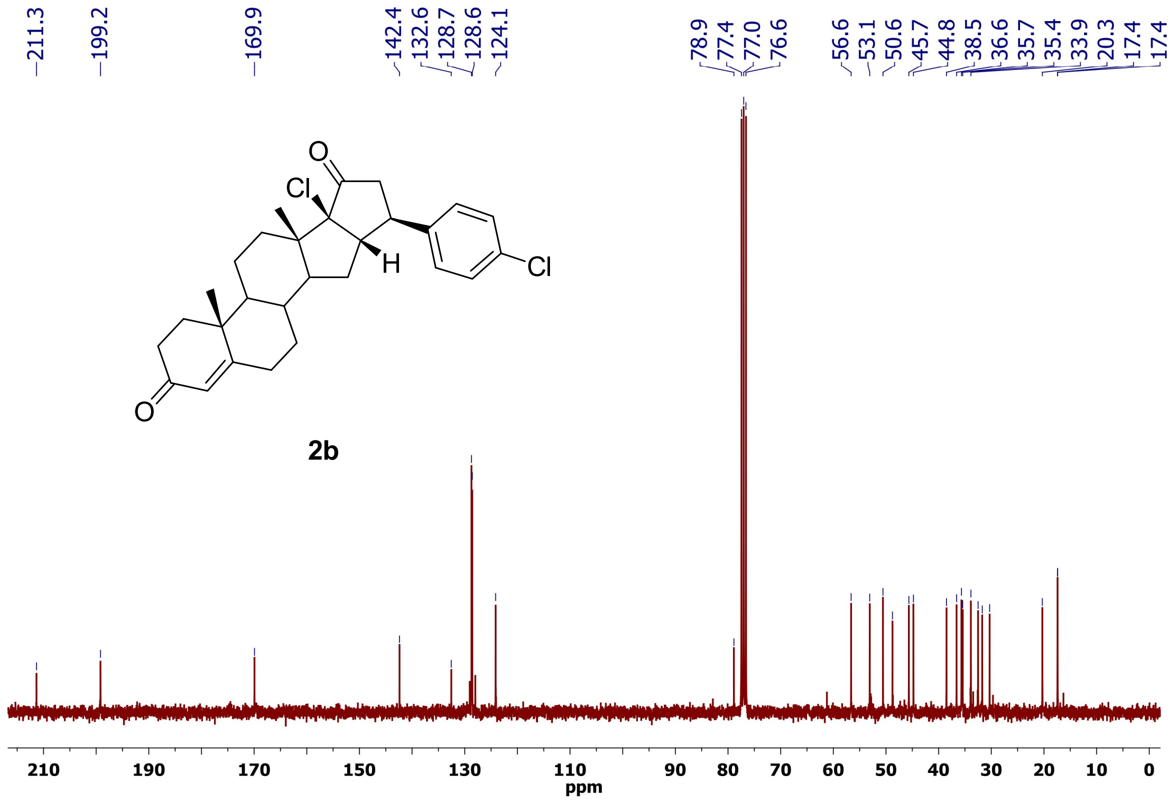

Compound 2c

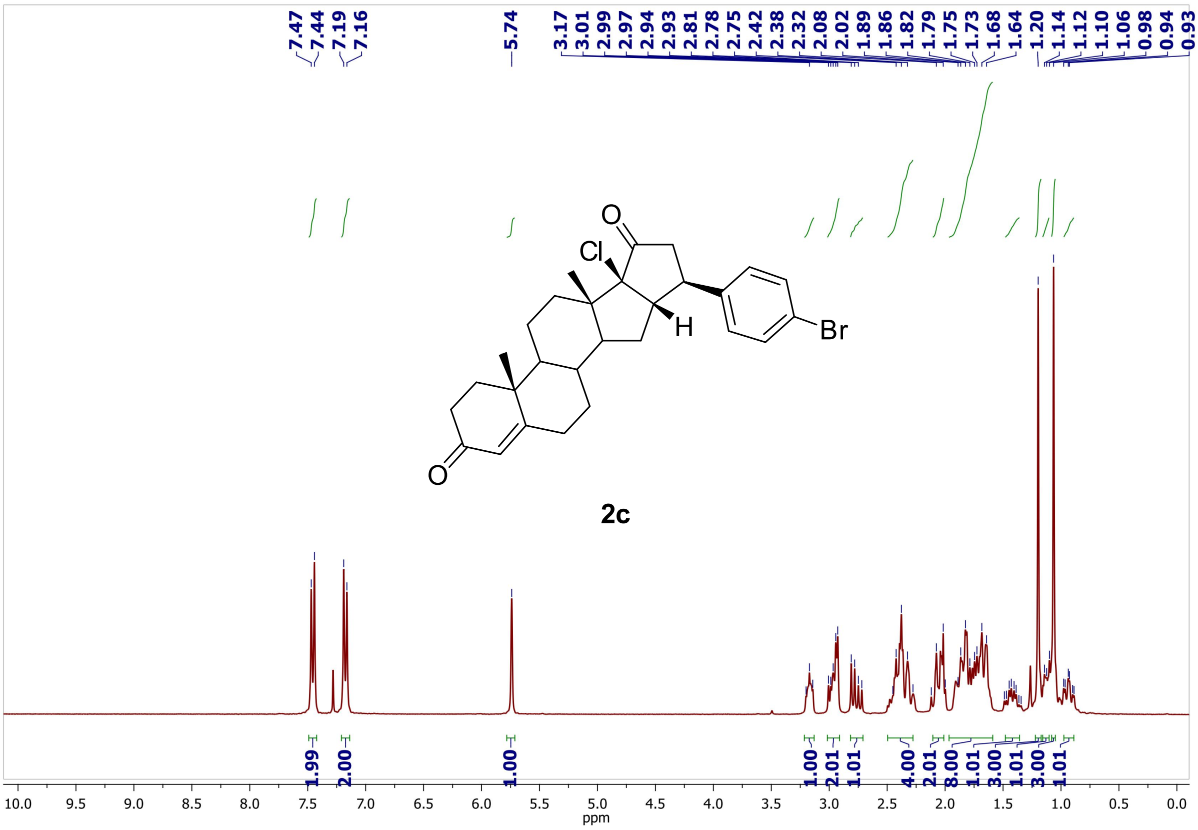
7.47
7.44
7.19
7.16
5.74
3.17 3.01 2.99 2.97 2.94 2.93 2.81 2.78 2.75 2.42 2.38 2.32 2.08 2.02 1.89 1.86 1.82 1.79 1.75 1.73 1.68 1.64 1.20 1.14 1.12 1.10 1.06 0.98 0.94 0.93
O
Cl
H
Br
O
2c
1.99
2.00
1.00
1.00 2.01 1.01 4.00 2.01 8.00 1.01 3.00 1.01 3.00 1.01
10.0 9.5 9.0 8.5 8.0 7.5 7.0 6.5 6.0 5.5 5.0 4.5 4.0 3.5 3.0 2.5 2.0 1.5 1.0 0.5 0.0
ppm

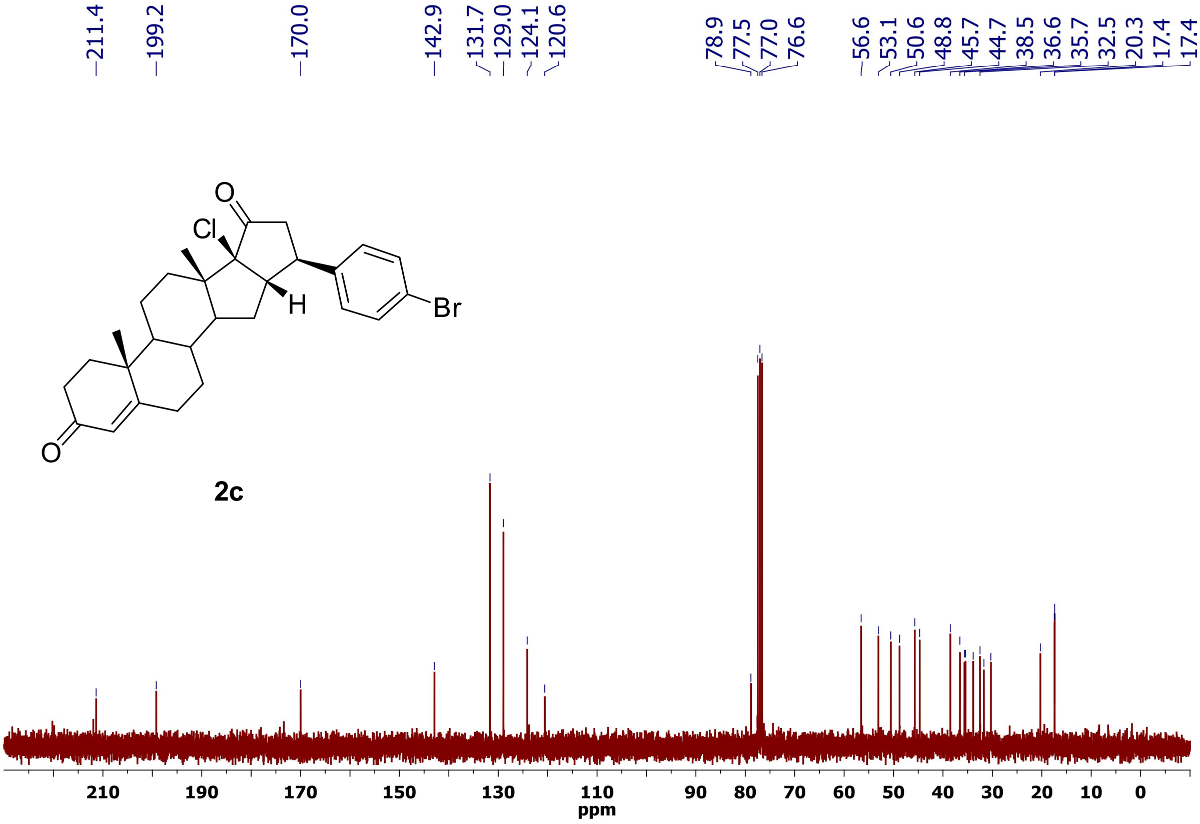
211.4
199.2
170.0
142.9
131.7
129.0
124.1
120.6
78.9
77.5
77.0
76.6
56.6 53.1 50.6 48.8 45.7 44.7 38.5 36.6 35.7 32.5 20.3 17.4 17.4
O
Cl
H
Br
O
2c
210 190 170 150 130 110 90 80 70 60 50 40 30 20 10 0
ppm

Compound 2d

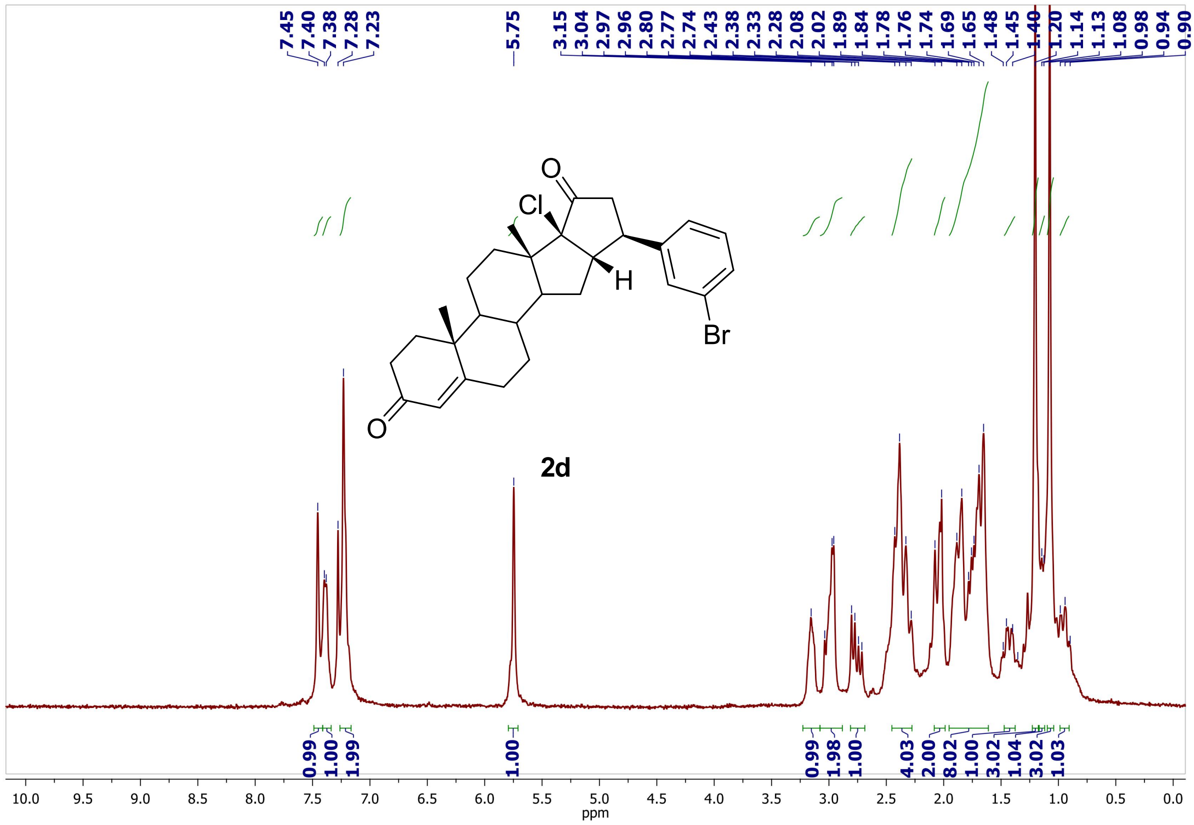

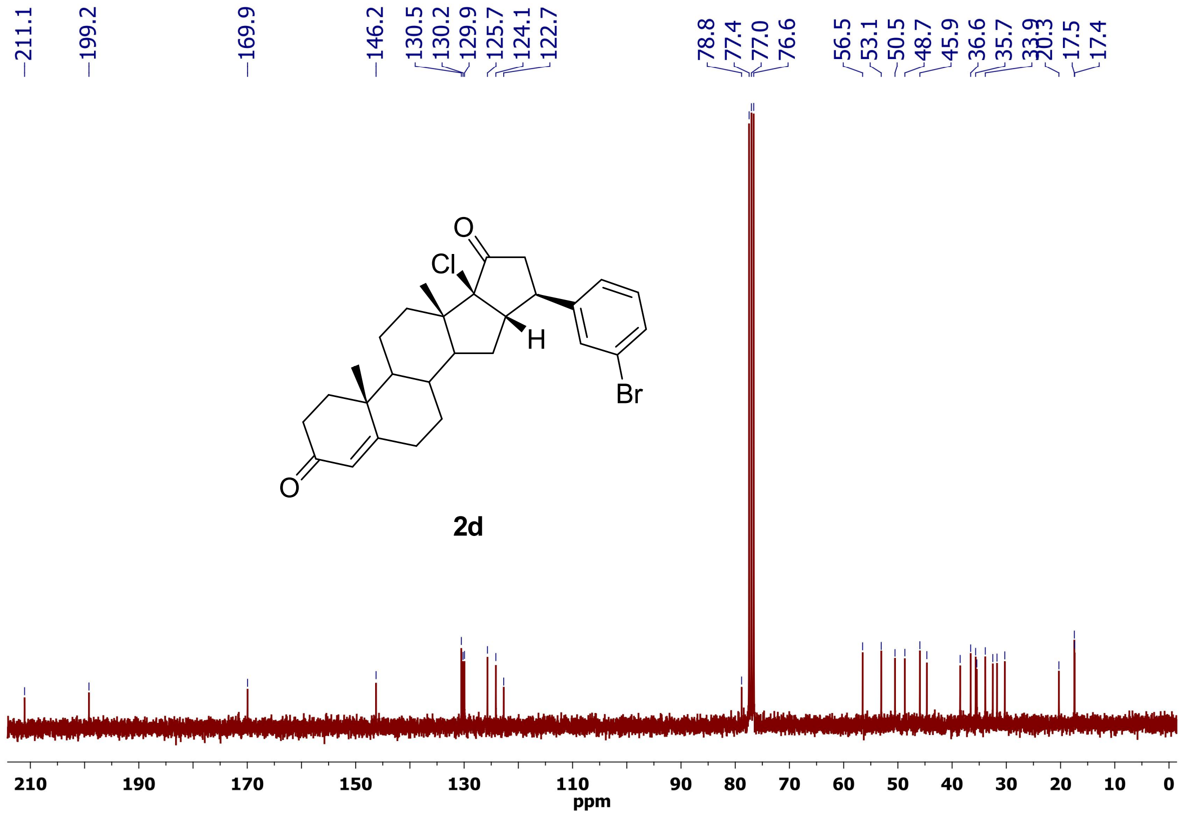

Compound 2e

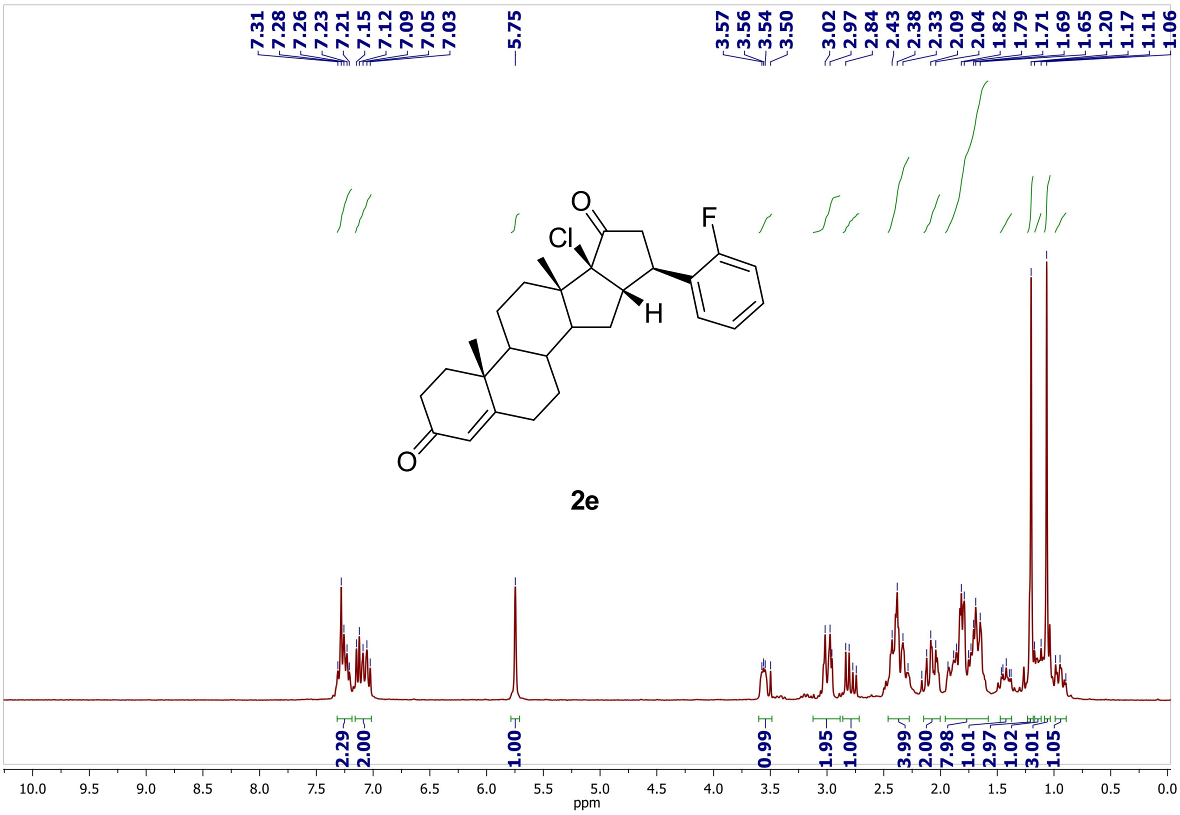
7.31
7.28
7.26
7.23
7.21
7.15
7.12
7.09
7.05
7.03
5.75
3.57
3.56
3.54
3.50
3.02
2.97
2.84
2.43
2.38
2.33
2.09
2.04
1.82
1.79
1.71
1.69
1.65
1.20
1.17
1.11
1.06
O
Cl
F
H
O
2e
2.29
2.00
1.00
0.99
1.95
1.00
3.99
2.00
7.98
1.01
2.97
1.02
3.01
1.05
ppm

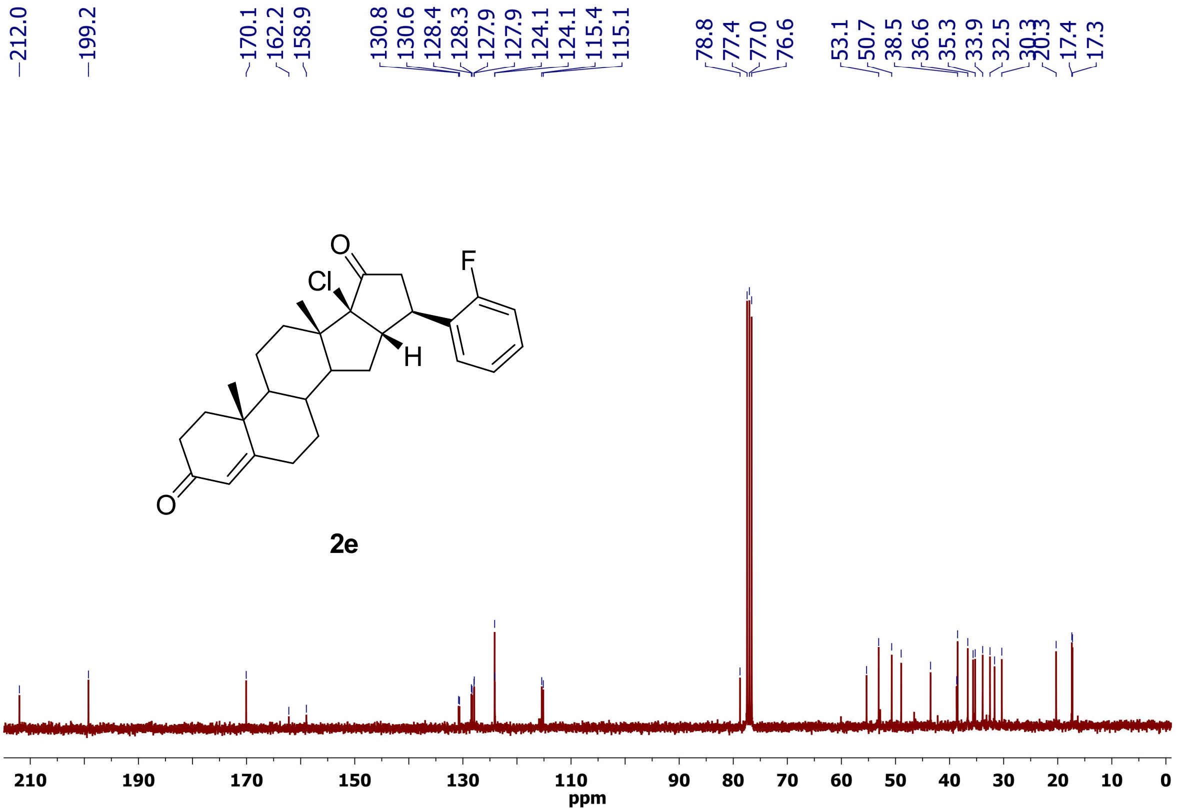
212.0
199.2
170.1
162.2
158.9
130.8
130.6
128.4
128.3
127.9
127.9
124.1
124.1
115.4
115.1
78.8
77.4
77.0
76.6
53.1
50.7
38.5
36.6
35.3
33.9
32.5
30.3
20.3
17.4
17.3
O
Cl
F
H
O
2e
ppm

Compound 2f

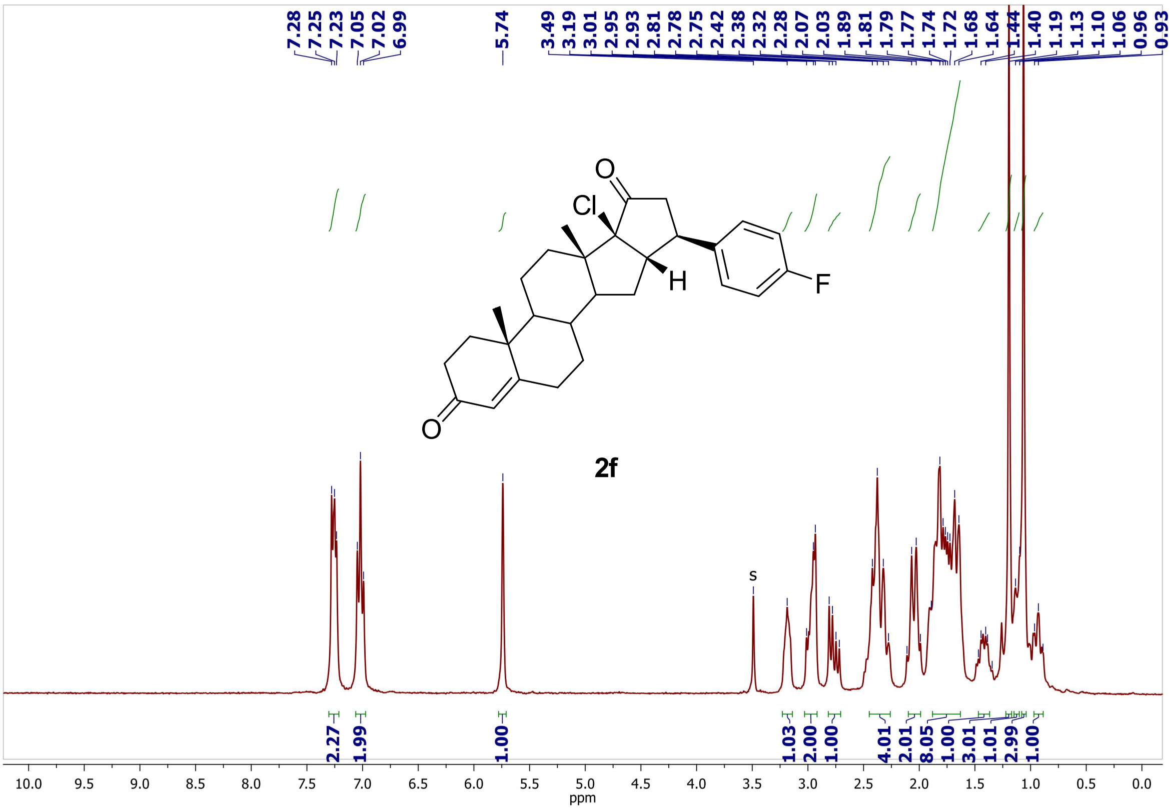

S - peak of methanol (3.49 ppm).

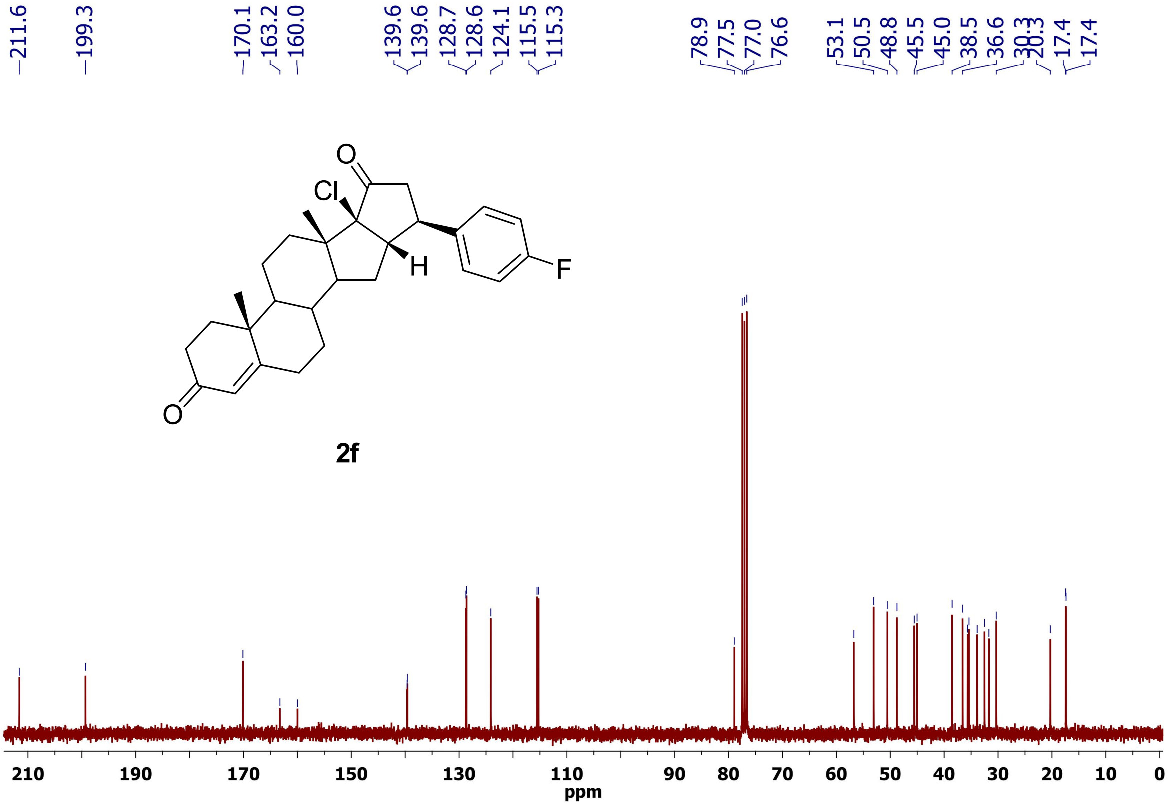

Compound 2g

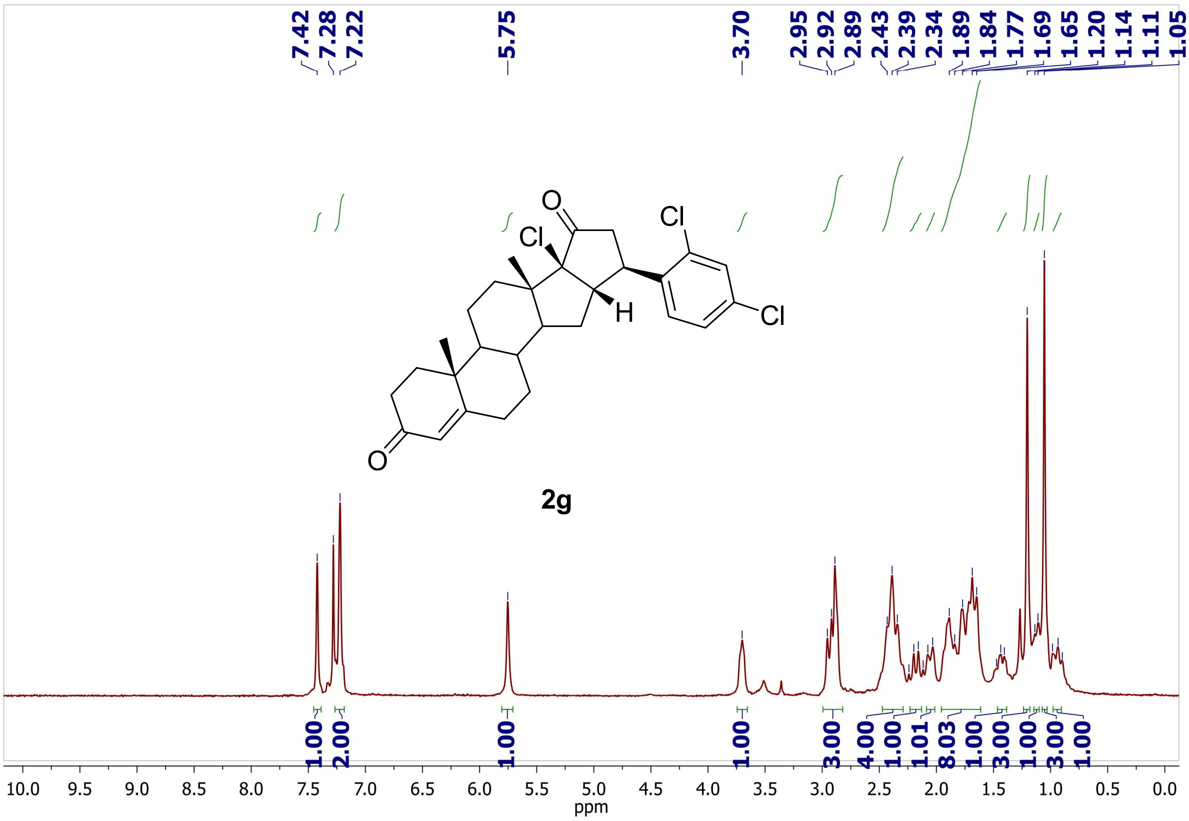
7.42
7.28
7.22
5.75
3.70
2.95
2.92
2.89
2.43
2.39
2.34
1.89
1.84
1.77
1.69
1.65
1.20
1.14
1.11
1.05
O
Cl
Cl
H
Cl
O
2g
1.00
2.00
1.00
1.00
3.00
4.00
1.00
1.01
8.03
1.00
3.00
1.00
3.00
1.00
10.0
9.5
9.0
8.5
8.0
7.5
7.0
6.5
6.0
5.5
5.0
4.5
4.0
3.5
3.0
2.5
2.0
1.5
1.0
0.5
0.0
ppm

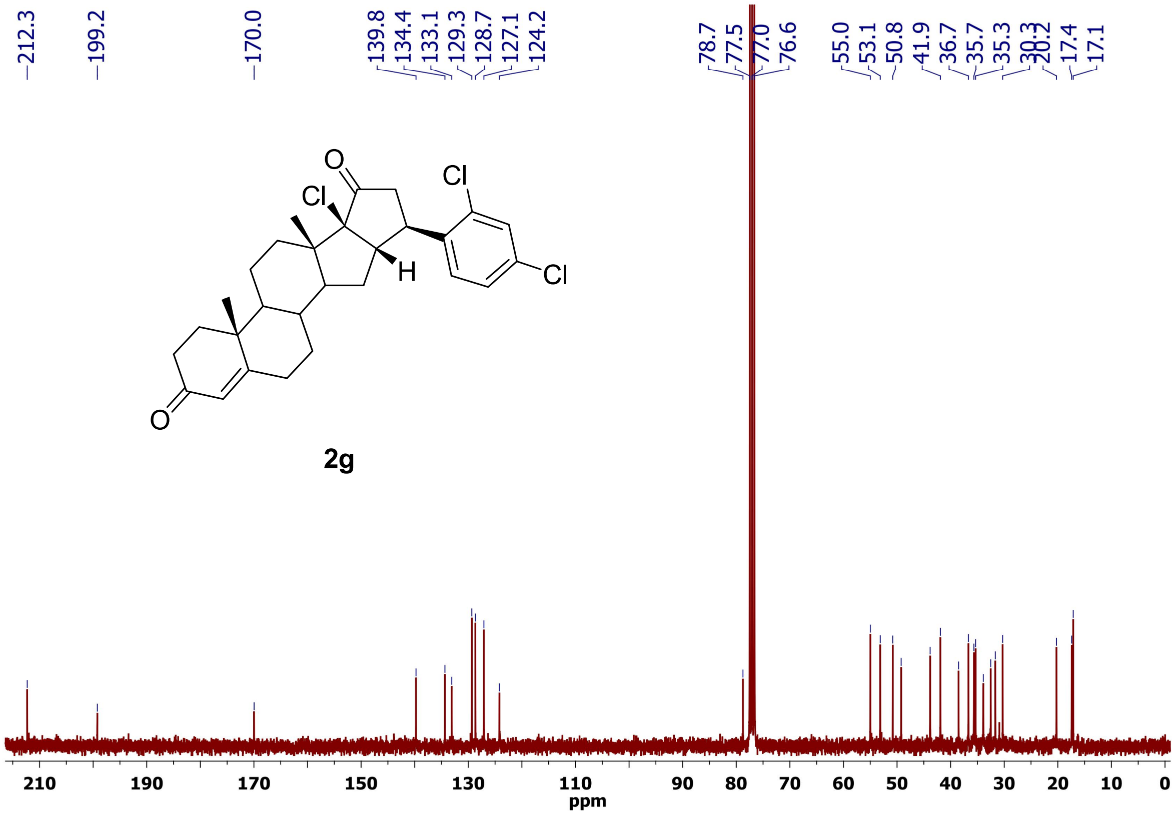
212.3
199.2
170.0
139.8
134.4
133.1
129.3
128.7
127.1
124.2
78.7
77.5
77.0
76.6
55.0
53.1
50.8
41.9
36.7
35.7
35.3
30.3
20.2
17.4
17.1
O
Cl
Cl
H
Cl
O
2g
210
190
170
150
130
110
90
80
70
60
50
40
30
20
10
0
ppm

Compound 2h

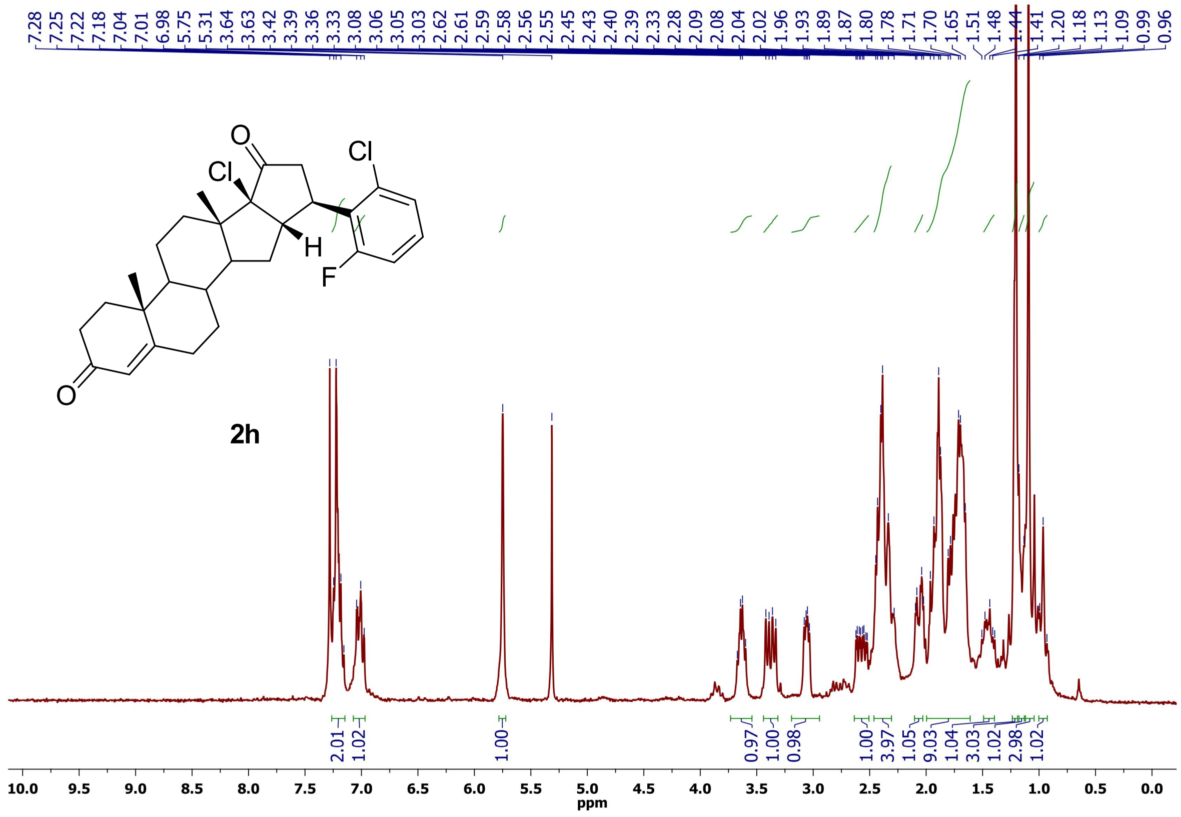

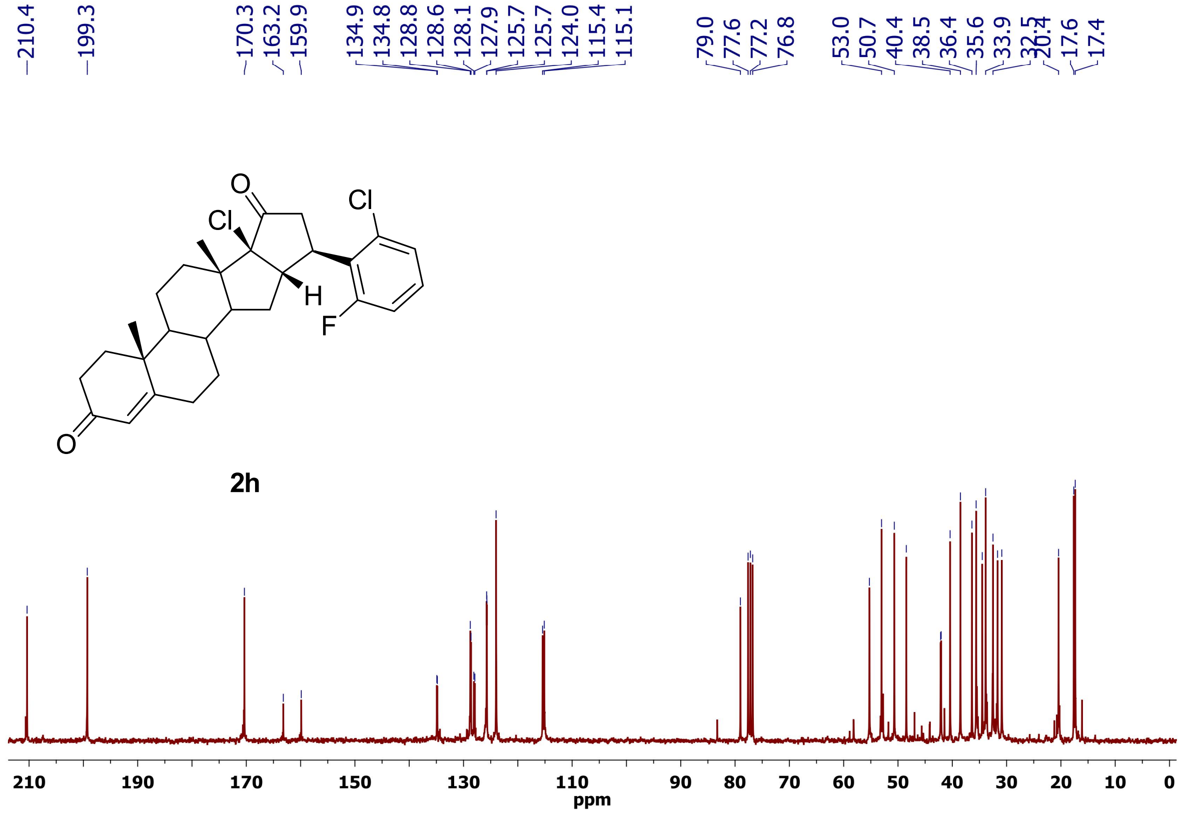

Compound 2i

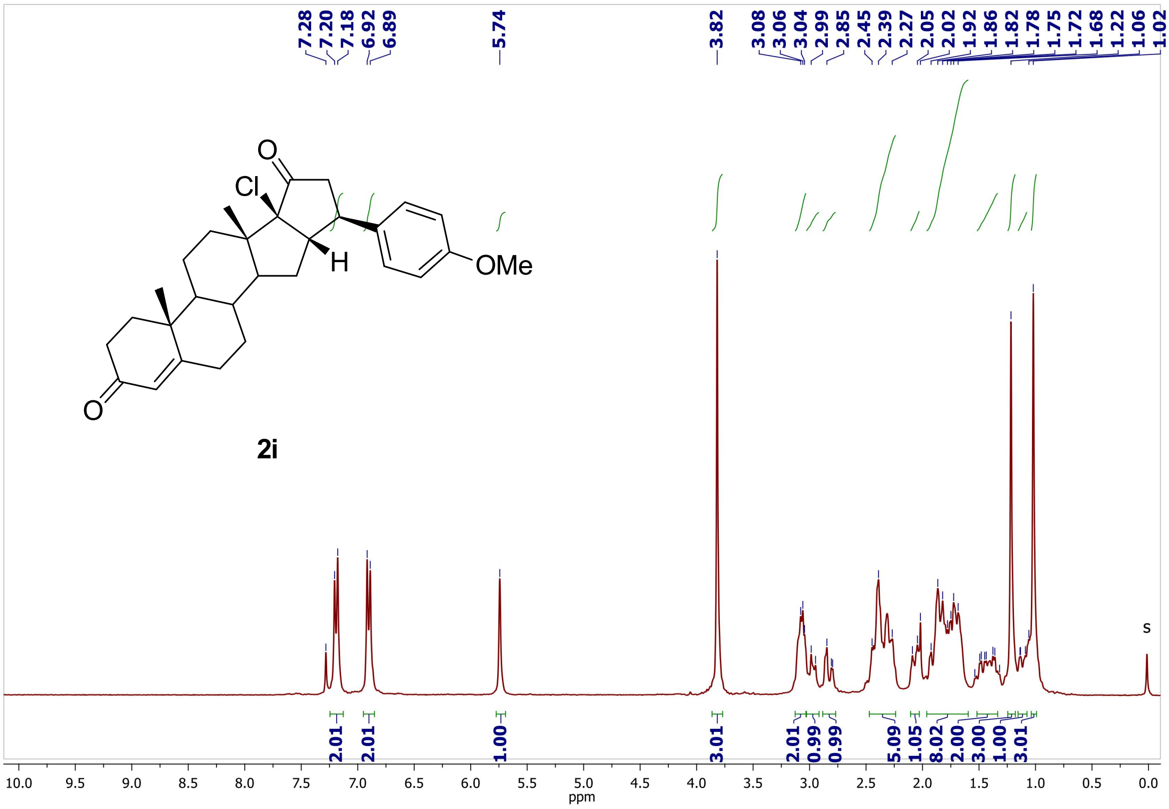

S - peak of silicone grease (0.01 ppm).

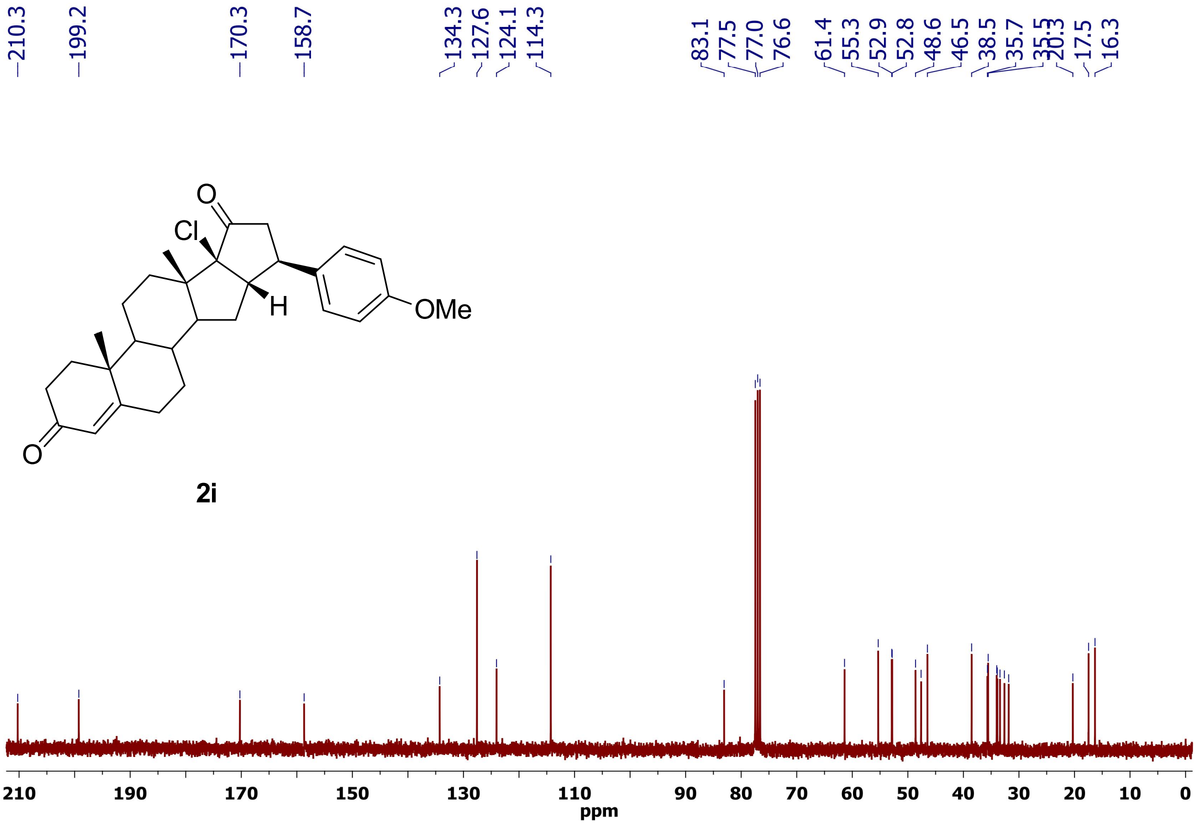

Compound 2j

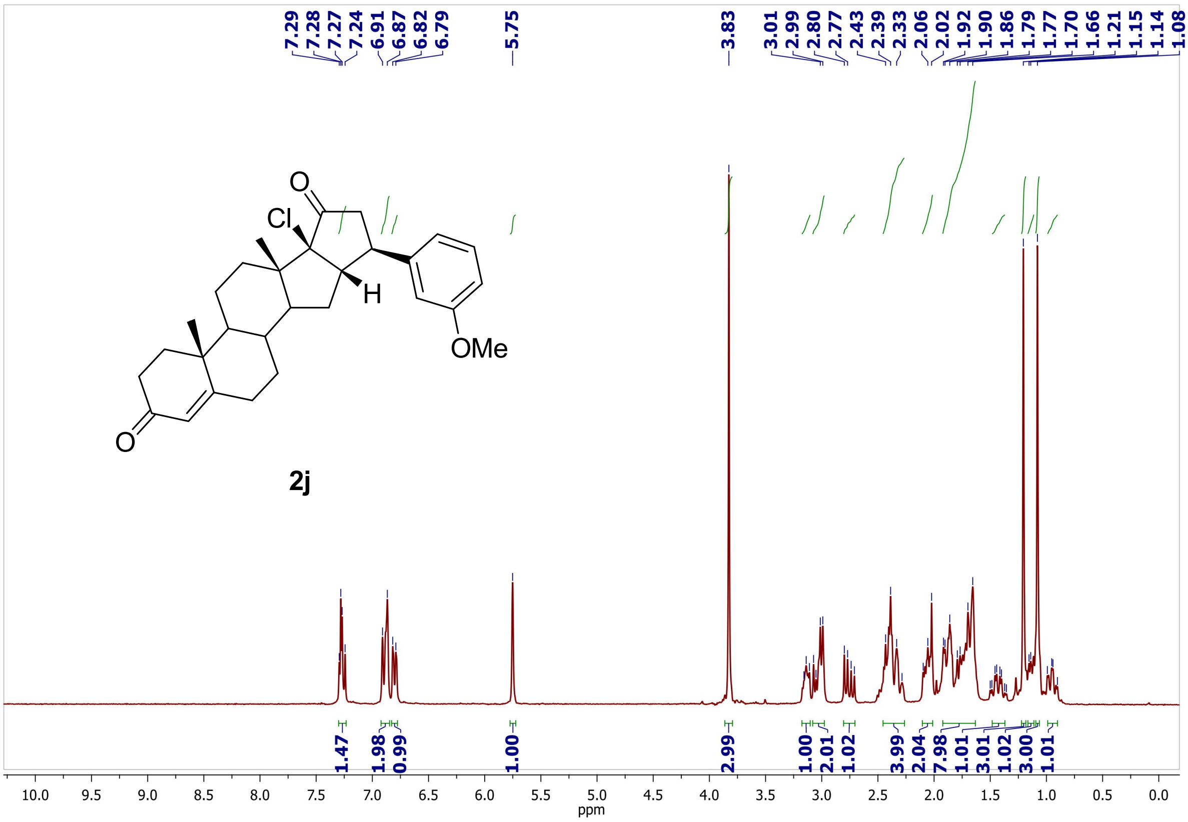

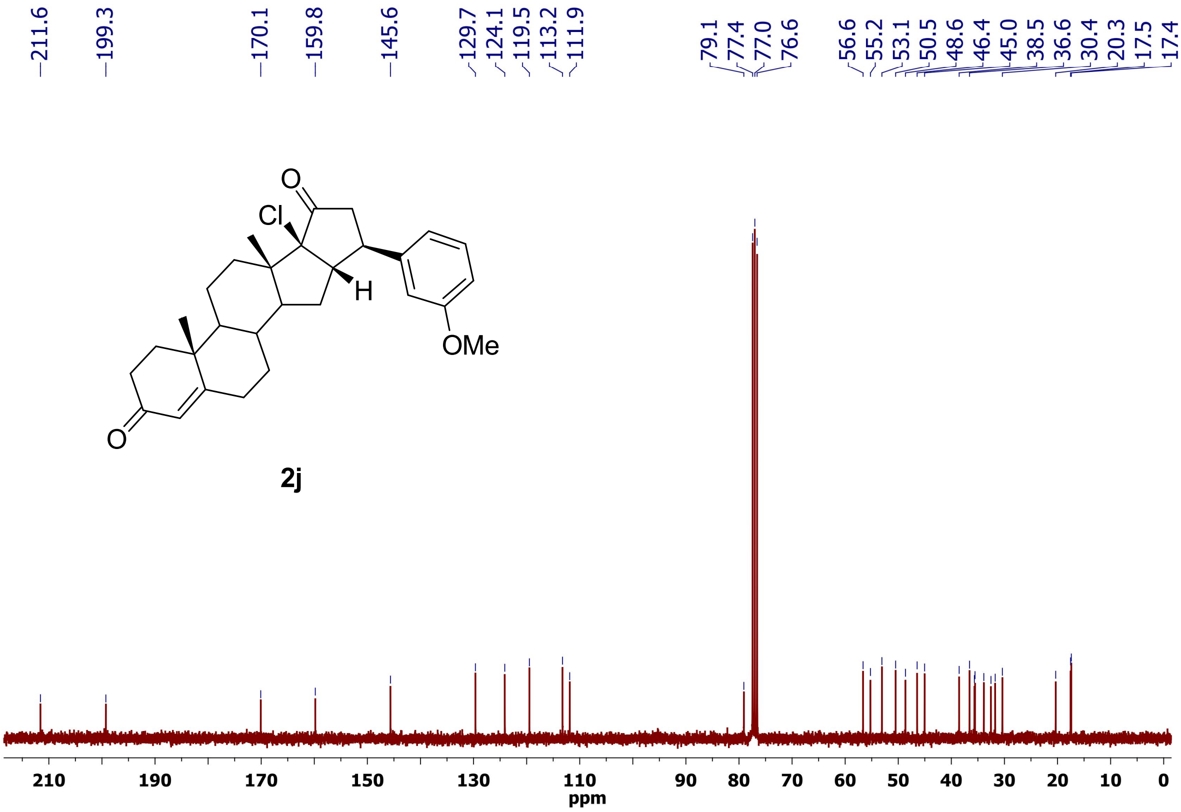

Compound 2k

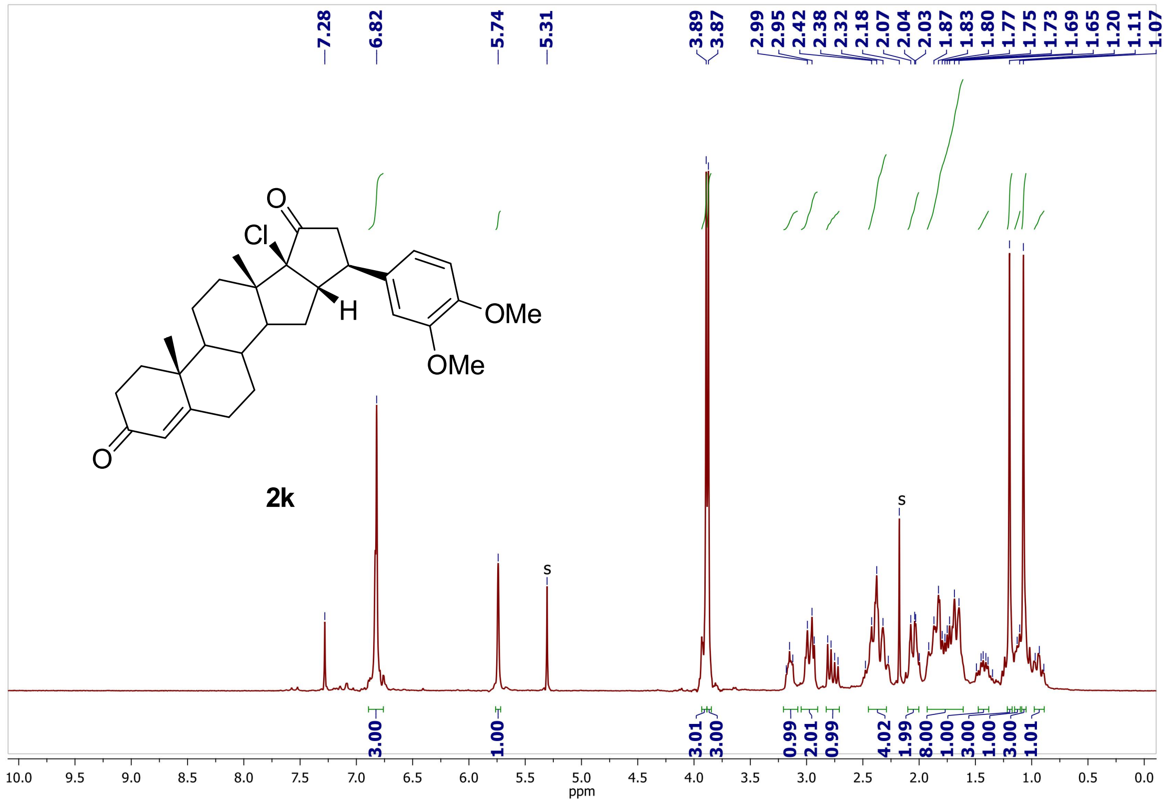

S - peaks of CH_2Cl_2 (5.31 ppm); acetone (2.18 ppm).

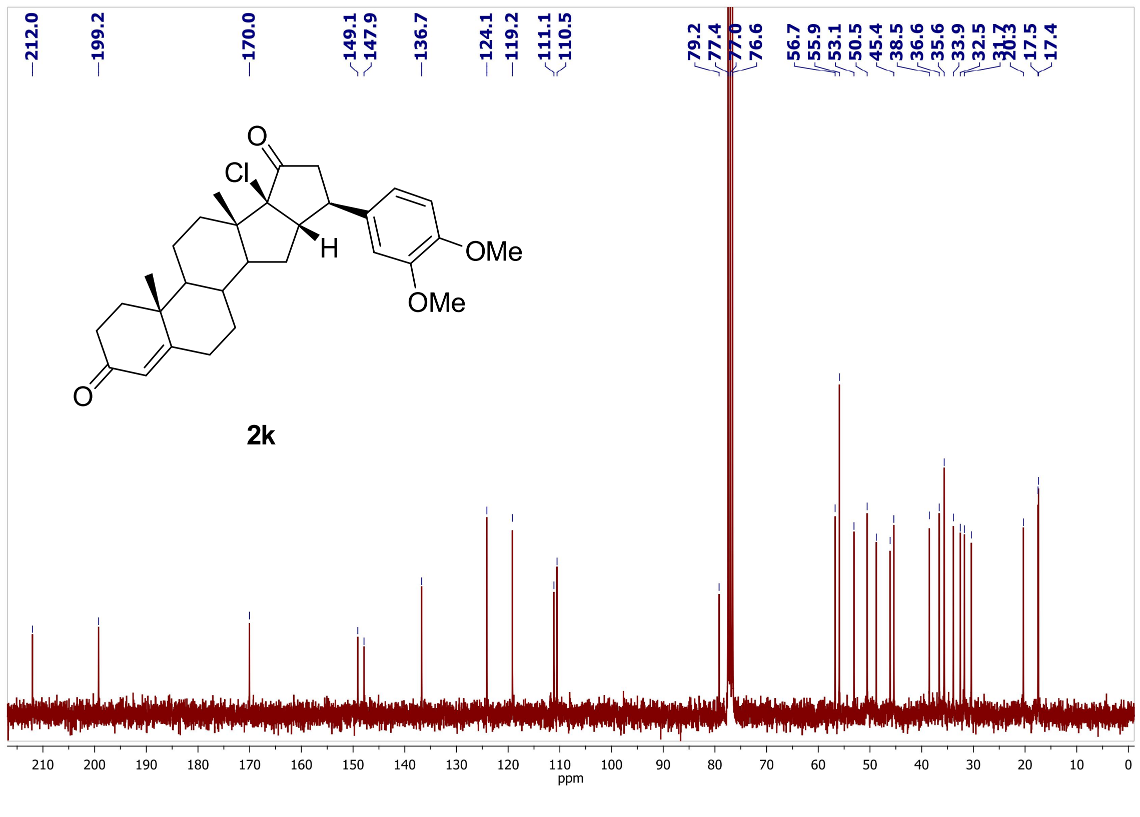

Compound 2l

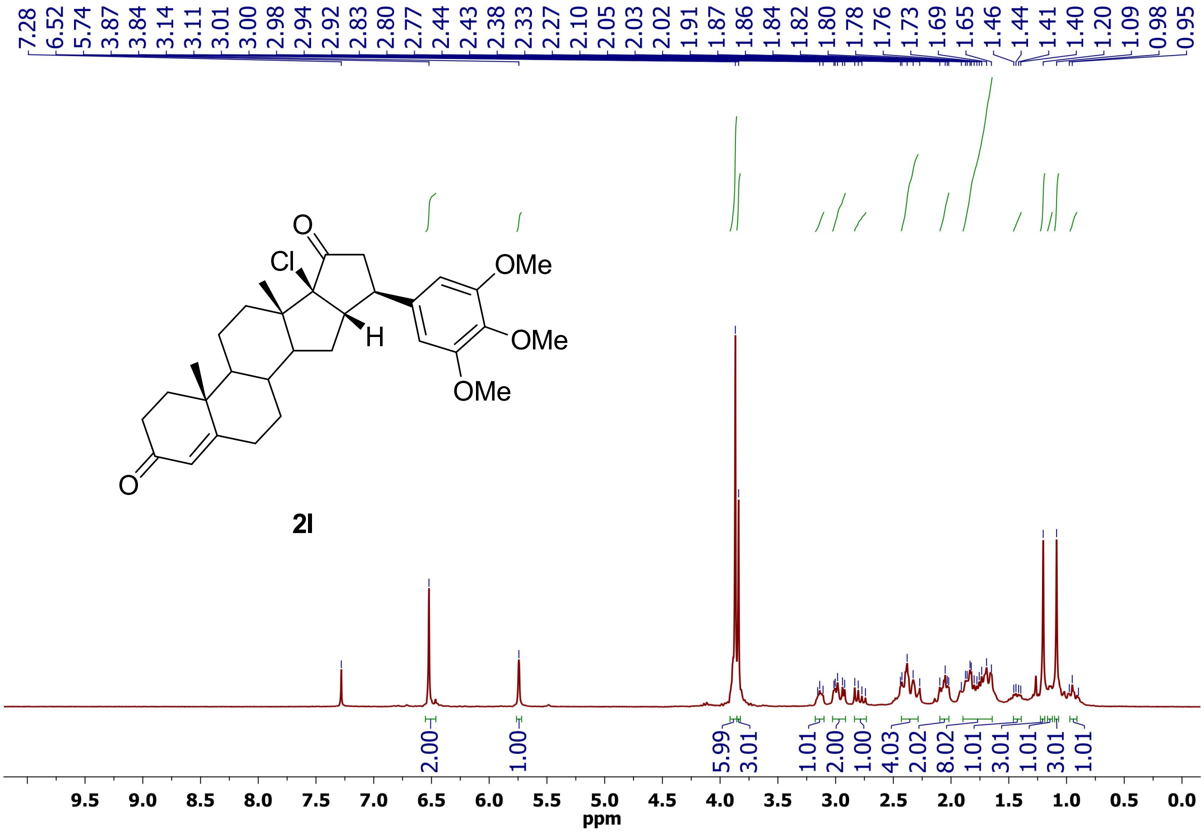

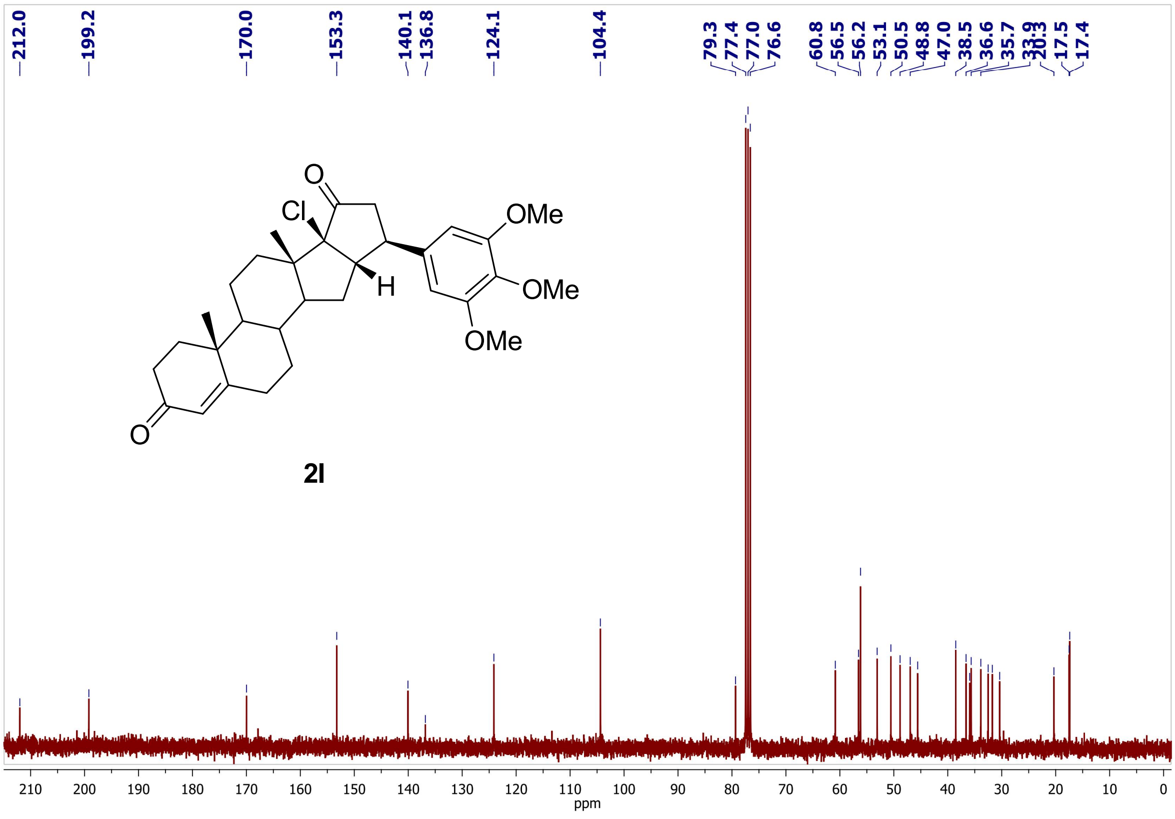

Compound 2m

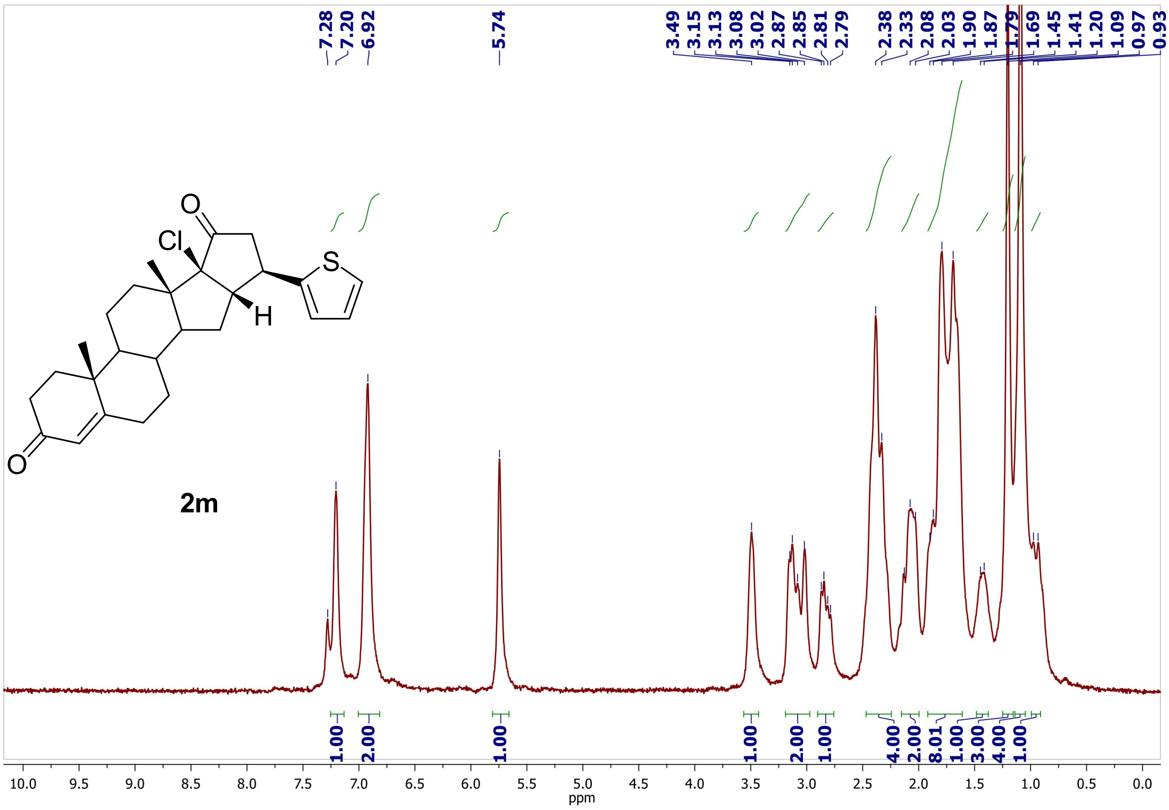

7.28
7.20
6.92
5.74
3.49
3.15
3.13
3.08
3.02
2.87
2.85
2.81
2.79
2.38
2.33
2.08
2.03
1.90
1.87
1.79
1.69
1.45
1.41
1.20
1.09
0.97
0.93
1.00
2.00
1.00
1.00
2.00
1.00
4.00
2.00
8.01
1.00
3.00
4.00
1.00
Cl
O
S
H
2m
ppm

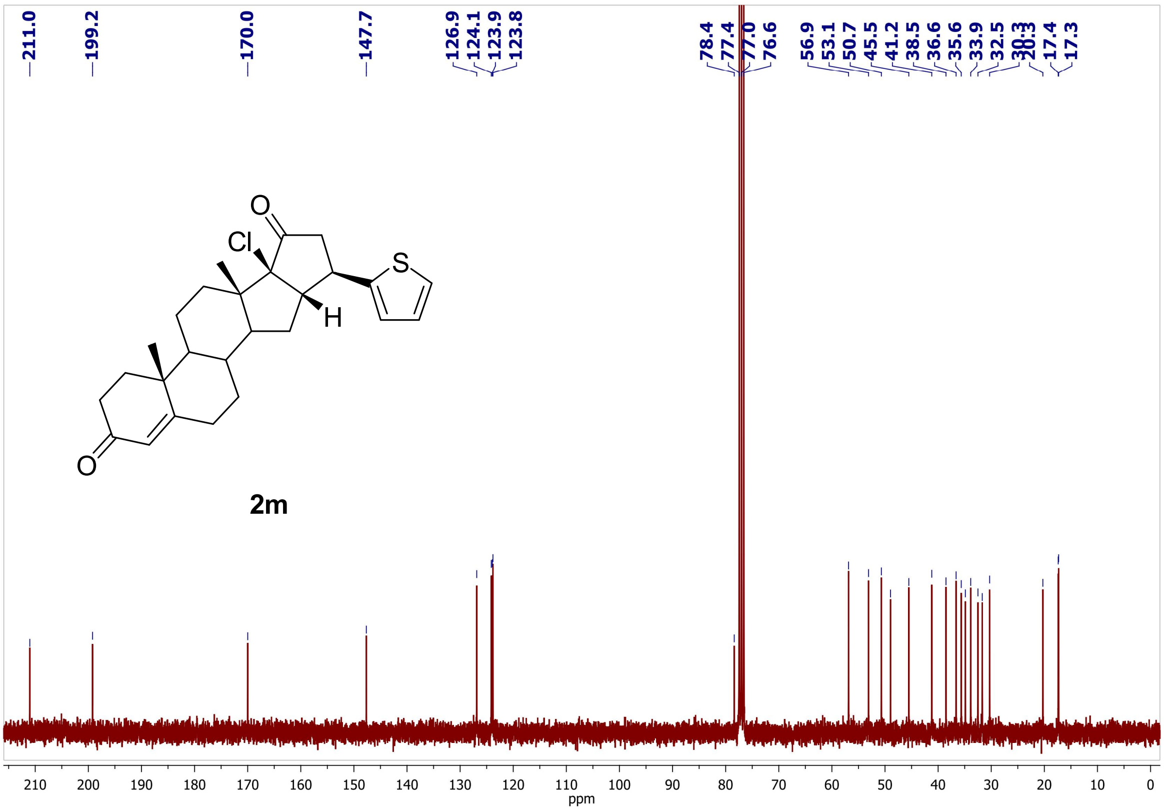

211.0
199.2
170.0
147.7
126.9
124.1
123.9
123.8
78.4
77.4
77.0
76.6
56.9
53.1
50.7
45.5
41.2
38.5
36.6
35.6
33.9
32.5
30.3
20.3
17.4
17.3
Cl
O
S
H
2m
ppm

VII. Copies of HRMS specta.

Compound 2a

Display Report

Analysis Info

Analysis Name	D:\Data\Kolotyrkina\2018\Zavarzin\1108014.d	Acquisition Date	08.11.2018 18:33:15
Method	tune_100-1200.m	Operator	BDAL@DE
Sample Name	/TBMK VRC166	Instrument	maXis 43
Comment	C28H33ClO2 mH 437.2241		

Acquisition Parameter

Source Type	ESI	Ion Polarity	Positive	Set Nebulizer	1.0 Bar
Focus	Active	Set Capillary	4500 V	Set Dry Heater	200 °C
Scan Begin	50 m/z	Set End Plate Offset	-500 V	Set Dry Gas	4.0 l/min
Scan End	1500 m/z	Set Collision Cell RF	1200.0 Vpp	Set Divert Valve	Waste

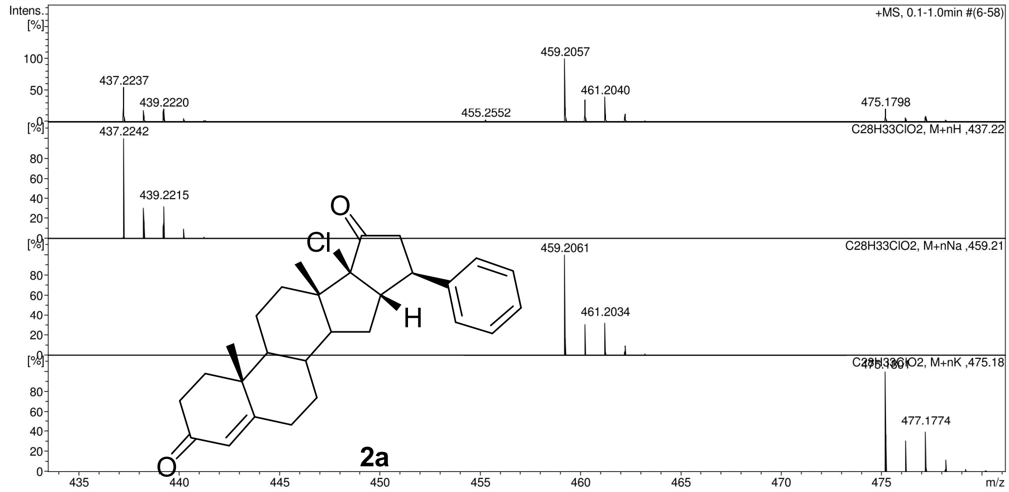

Bruker Compass DataAnalysis 4.0 printed: 08.11.2018 18:37:37 Page 1 of 1

Compound 2b

Display Report

Analysis Info

Analysis Name	D:\Data\Kolotyrkina\2018\Zavarzin\1113040.d	Acquisition Date	13.11.2018 15:29:27
Method	tune_50-1600.m	Operator	BDAL@DE
Sample Name	/CHER VRC-194	Instrument / Ser#	micrOTOF 10248
Comment	C28H32Cl2O2 mH 471.1852 calibrant added		

Acquisition Parameter

Source Type	ESI	Ion Polarity	Positive	Set Nebulizer	1.0 Bar
Focus	Not active			Set Dry Heater	200 °C
Scan Begin	50 m/z	Set Capillary	4500 V	Set Dry Gas	4.0 l/min
Scan End	1600 m/z	Set End Plate Offset	-500 V	Set Divert Valve	Waste

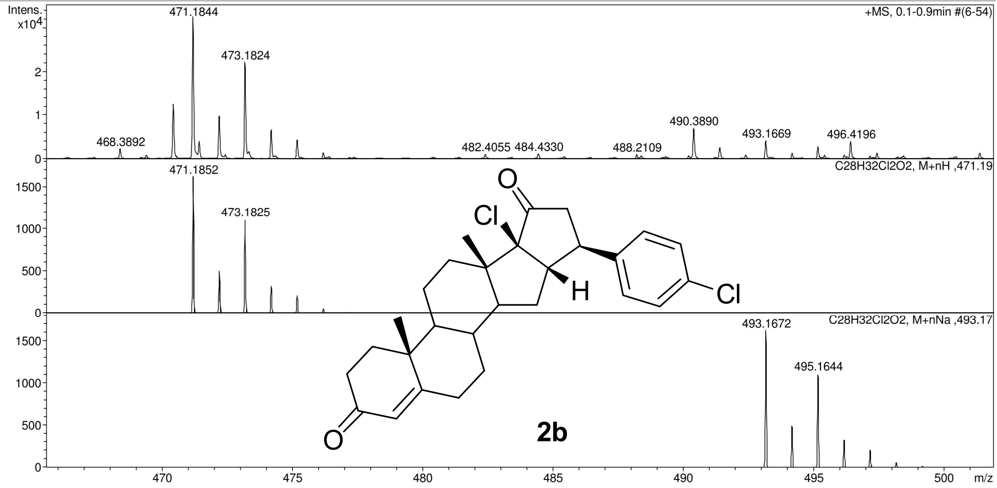

Compound 2c

Display Report

**Analysis Info**		Acquisition Date	15.11.2018 16:24:01
Analysis Name	D:\Data\Kolotyrkina\2018\Zavarzin\1115024.d		
Method	tune_50-1600.m	Operator	BDAL@DE
Sample Name	/TBMK VRC-245	Instrument / Ser#	micrOTOF 10248
Comment	C28H32BrClO2 mH 515.1346/ calibrant added		

Acquisition Parameter

Source Type	ESI	Ion Polarity	Positive	Set Nebulizer	1.0 Bar
Focus	Not active			Set Dry Heater	200 °C
Scan Begin	50 m/z	Set Capillary	4500 V	Set Dry Gas	4.0 l/min
Scan End	1600 m/z	Set End Plate Offset	-500 V	Set Divert Valve	Waste

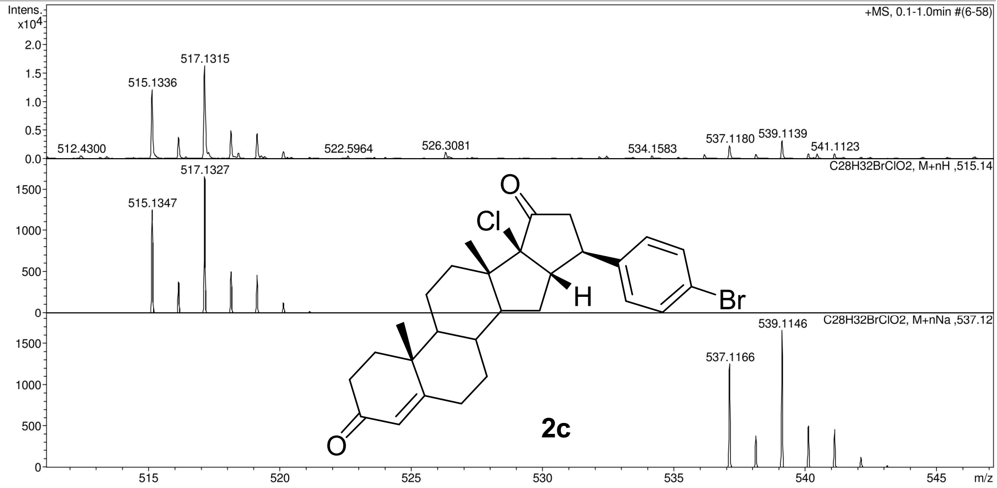

Compound 2d

Display Report

Analysis Info

Analysis Name	D:\Data\Kolotyrkina\2018\Zavarzin\1115023.d	Acquisition Date	15.11.2018 16:12:26
Method	tune_50-1600.m	Operator	BDAL@DE
Sample Name	/TBMK VRC-207	Instrument / Ser#	micrOTOF 10248
Comment	C28H32BrClO2 mH 515.1346/ calibrant added		

Acquisition Parameter

Source Type	ESI	Ion Polarity	Positive	Set Nebulizer	1.0 Bar
Focus	Not active			Set Dry Heater	200 °C
Scan Begin	50 m/z	Set Capillary	4500 V	Set Dry Gas	4.0 l/min
Scan End	1600 m/z	Set End Plate Offset	-500 V	Set Divert Valve	Waste

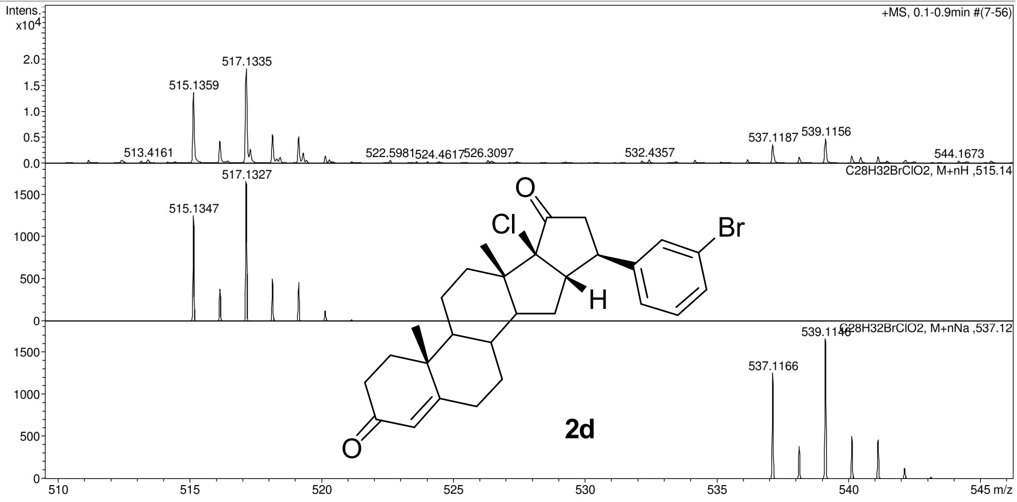

Compound 2e

Display Report

Analysis Info

Analysis Name	D:\Data\Kolotyrkina\2018\Zavarzin\1101006.d	Acquisition Date	01.11.2018 13:10:22
Method	tune_50-1600.m	Operator	BDAL@DE
Sample Name	/TBMK VRC243	Instrument / Ser#	micrOTOF 10248
Comment	C28H32ClFO2 mH 455.2147 calibrant added		

Acquisition Parameter

Source Type	ESI	Ion Polarity	Positive	Set Nebulizer	1.0 Bar
Focus	Not active			Set Dry Heater	200 °C
Scan Begin	50 m/z	Set Capillary	4500 V	Set Dry Gas	4.0 l/min
Scan End	1600 m/z	Set End Plate Offset	-500 V	Set Divert Valve	Waste

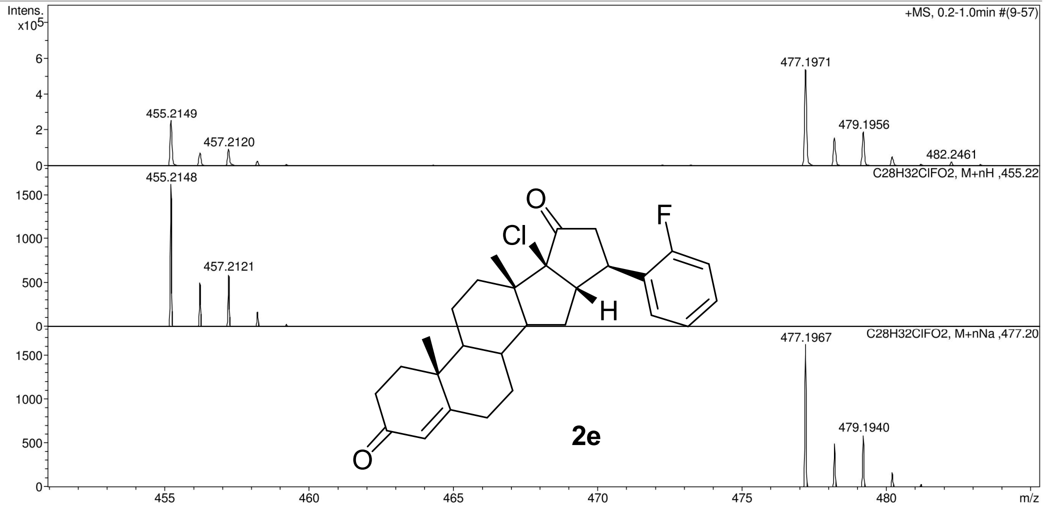

Compound 2f

Display Report

Analysis Info

Analysis Name	D:\Data\Kolotyrkina\2018\Zavarzin\1101004.d	Acquisition Date	01.11.2018 12:58:01
Method	tune_50-1600.m	Operator	BDAL@DE
Sample Name	/TBMK VRC241	Instrument / Ser#	micrOTOF 10248
Comment	C28H32ClFO2 mH 455.2147 calibrant added		

Acquisition Parameter

Source Type	ESI	Ion Polarity	Positive	Set Nebulizer	1.0 Bar
Focus	Not active			Set Dry Heater	200 °C
Scan Begin	50 m/z	Set Capillary	4500 V	Set Dry Gas	4.0 l/min
Scan End	1600 m/z	Set End Plate Offset	-500 V	Set Divert Valve	Waste

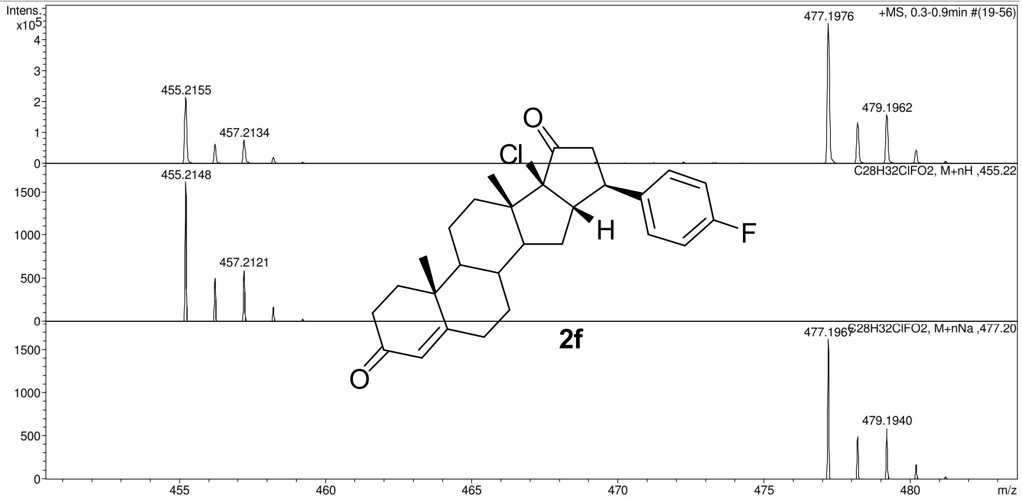

Compound 2g

Display Report

Analysis Info

Analysis Name	D:\Data\Kolotyrkina\2018\Zavarzin\1108012.d	Acquisition Date	08.11.2018 18:16:45
Method	tune_100-1200.m	Operator	BDAL@DE
Sample Name	/TBMK VRC179	Instrument	maXis 43
Comment	C28H31Cl3O2 mH 505.1462		

Acquisition Parameter

Source Type	ESI	Ion Polarity	Positive	Set Nebulizer	1.0 Bar
Focus	Active	Set Capillary	4500 V	Set Dry Heater	200 °C
Scan Begin	50 m/z	Set End Plate Offset	-500 V	Set Dry Gas	4.0 l/min
Scan End	1500 m/z	Set Collision Cell RF	1200.0 Vpp	Set Divert Valve	Waste

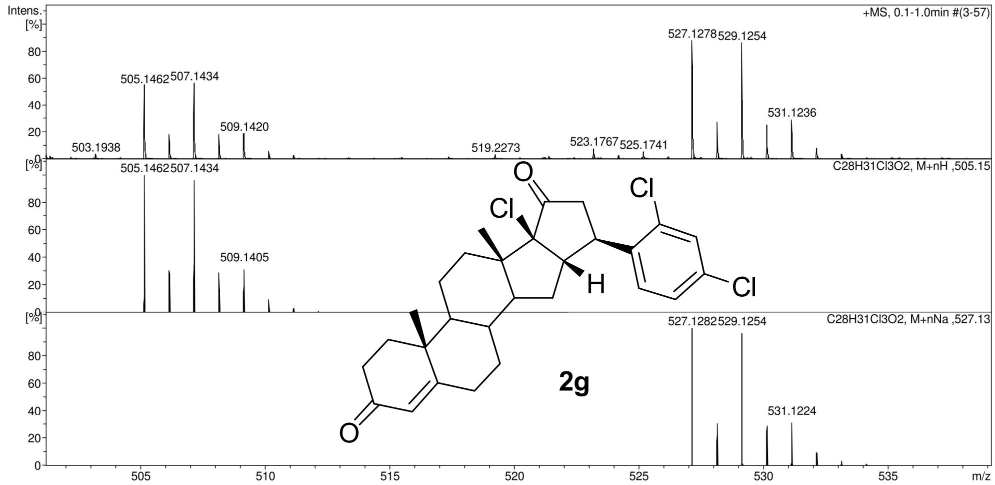

Compound 2h

Display Report

Analysis Info

Analysis Name	D:\Data\Kolotyrkina\2018\Zavarzin\1115022.d	Acquisition Date	15.11.2018 16:04:18
Method	tune_50-1600.m	Operator	BDAL@DE
Sample Name	/TBMK VRC-185	Instrument / Ser#	micrOTOF 10248
Comment	C28H31Cl2FO2 mH 489.1757 calibrant added		

Acquisition Parameter

Source Type	ESI	Ion Polarity	Positive	Set Nebulizer	1.0 Bar
Focus	Not active			Set Dry Heater	200 °C
Scan Begin	50 m/z	Set Capillary	4500 V	Set Dry Gas	4.0 l/min
Scan End	1600 m/z	Set End Plate Offset	-500 V	Set Divert Valve	Waste

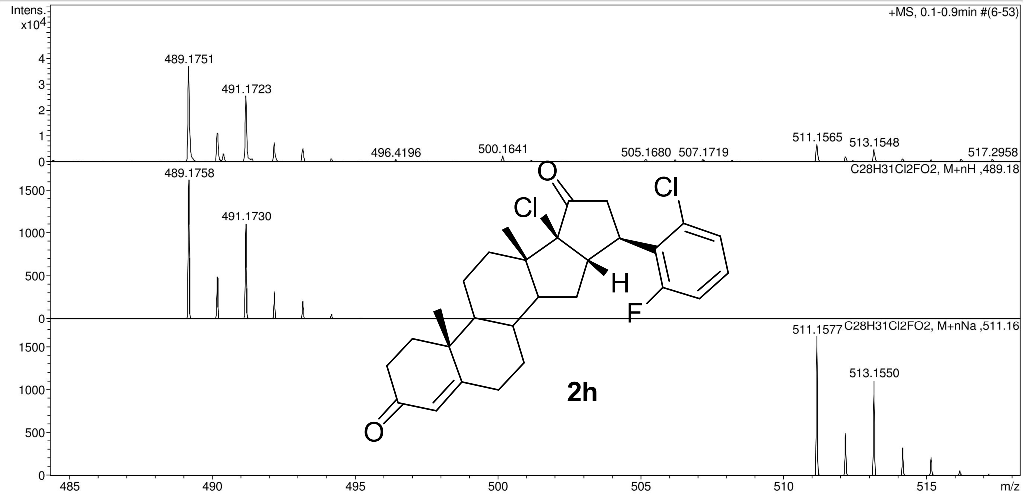

Compound 2i

Display Report

Analysis Info

Analysis Name	D:\Data\Kolotyrkina\2018\Zavarzin\1108015.d	Acquisition Date	08.11.2018 18:41:01
Method	tune_100-1200.m	Operator	BDAL@DE
Sample Name	/TBMK VRC173	Instrument	maXis 43
Comment	C29H35ClO3 mH 467.2347		

Acquisition Parameter

Source Type	ESI	Ion Polarity	Positive	Set Nebulizer	1.0 Bar
Focus	Active	Set Capillary	4500 V	Set Dry Heater	200 °C
Scan Begin	50 m/z	Set End Plate Offset	-500 V	Set Dry Gas	4.0 l/min
Scan End	1500 m/z	Set Collision Cell RF	1200.0 Vpp	Set Divert Valve	Waste

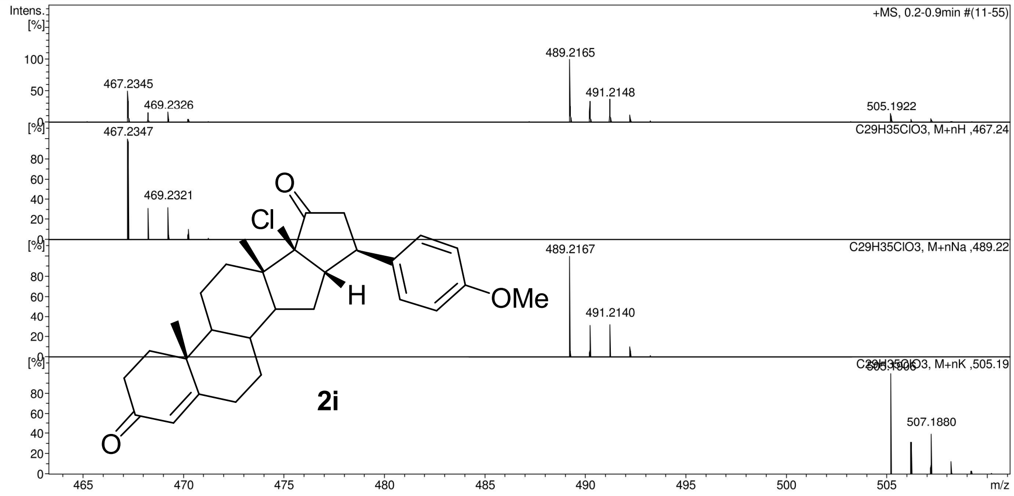

Compound 2j

Display Report

Analysis Info

Analysis Name	D:\Data\Kolotyrkina\2018\Zavarzin\1101005.d	Acquisition Date	01.11.2018 13:04:55
Method	tune_50-1600.m	Operator	BDAL@DE
Sample Name	/TBMK VRC242	Instrument / Ser#	micrOTOF 10248
Comment	C29H35ClO3 mH 467.2347 calibrant added		

Acquisition Parameter

Source Type	ESI	Ion Polarity	Positive	Set Nebulizer	1.0 Bar
Focus	Not active			Set Dry Heater	200 °C
Scan Begin	50 m/z	Set Capillary	4500 V	Set Dry Gas	4.0 l/min
Scan End	1600 m/z	Set End Plate Offset	-500 V	Set Divert Valve	Waste

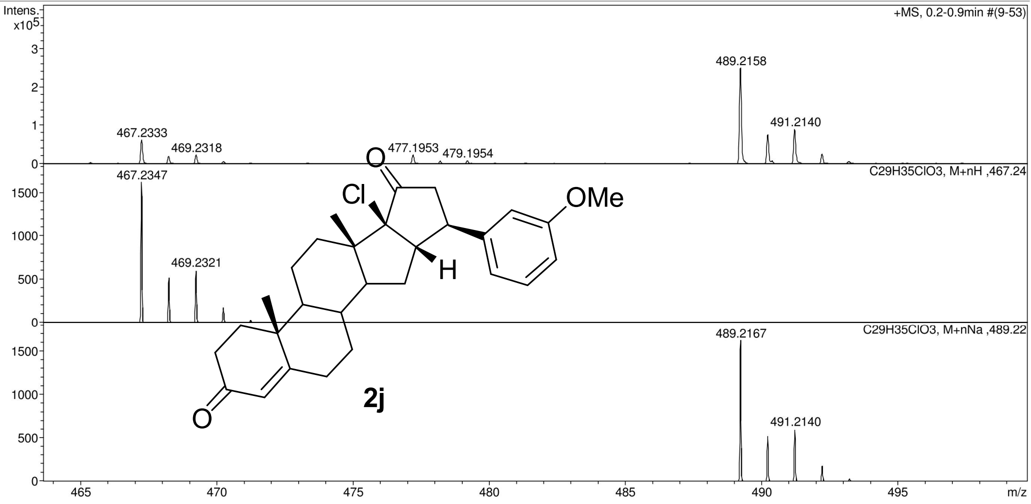

Compound 2k

Display Report

Analysis Info

Analysis Name	D:\Data\Kolotyrkina\2018\Zavarzin\1108013.d	Acquisition Date	08.11.2018 18:24:33
Method	tune_100-1200.m	Operator	BDAL@DE
Sample Name	/TBMK VRC174	Instrument	maXis 43
Comment	C30H37ClO4 mH 497.2453		

Acquisition Parameter

Source Type	ESI	Ion Polarity	Positive	Set Nebulizer	1.0 Bar
Focus	Active	Set Capillary	4500 V	Set Dry Heater	200 °C
Scan Begin	50 m/z	Set End Plate Offset	-500 V	Set Dry Gas	4.0 l/min
Scan End	1500 m/z	Set Collision Cell RF	1200.0 Vpp	Set Divert Valve	Waste

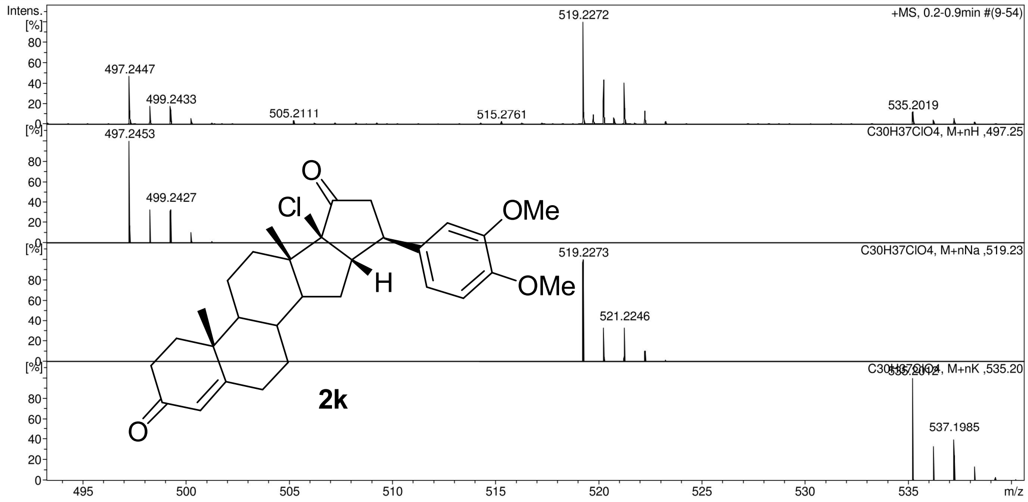

Compound 2l

Display Report

Analysis Info

Analysis Name	D:\Data\Kolotyrkina\2018\Zavarzin\1113038.d	Acquisition Date	13.11.2018 15:14:54
Method	tune_50-1600.m	Operator	BDAL@DE
Sample Name	/CHER VRC-176	Instrument / Ser#	micrOTOF 10248
Comment	C31H39ClO5 mH 527.2558 calibrant added		

Acquisition Parameter

Source Type	ESI	Ion Polarity	Positive	Set Nebulizer	1.0 Bar
Focus	Not active			Set Dry Heater	200 °C
Scan Begin	50 m/z	Set Capillary	4500 V	Set Dry Gas	4.0 l/min
Scan End	1600 m/z	Set End Plate Offset	-500 V	Set Divert Valve	Waste

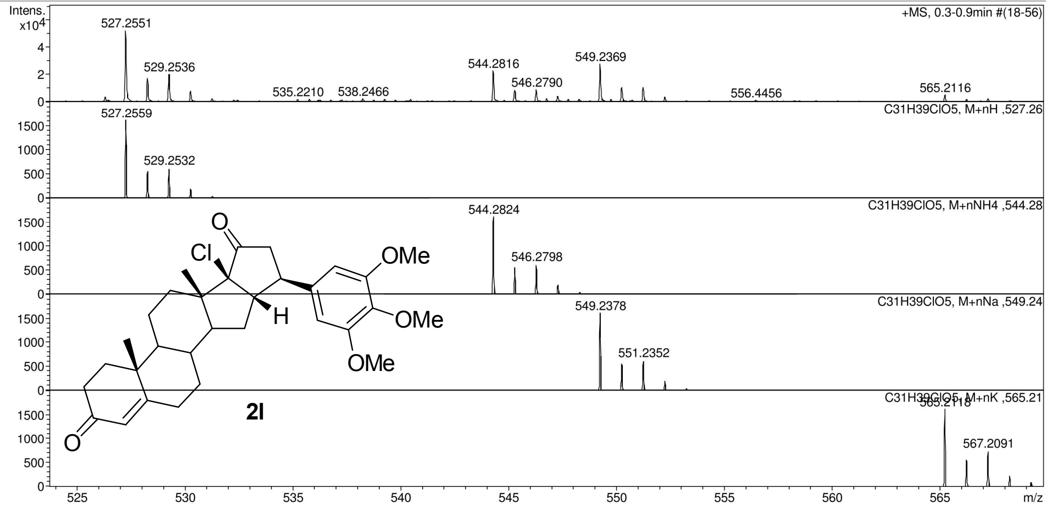

Compound 2m

Display Report

Analysis Info

Analysis Name	D:\Data\Kolotyrkina\2018\Zavarzin\1113041.d	Acquisition Date	13.11.2018 15:35:18
Method	tune_50-1600.m	Operator	BDAL@DE
Sample Name	/CHER VRC-238	Instrument / Ser#	micrOTOF 10248
Comment	C26H31ClO2S mH 443.1806 calibrant added		

Acquisition Parameter

Source Type	ESI	Ion Polarity	Positive	Set Nebulizer	1.0 Bar
Focus	Not active			Set Dry Heater	200 °C
Scan Begin	50 m/z	Set Capillary	4500 V	Set Dry Gas	4.0 l/min
Scan End	1600 m/z	Set End Plate Offset	-500 V	Set Divert Valve	Waste

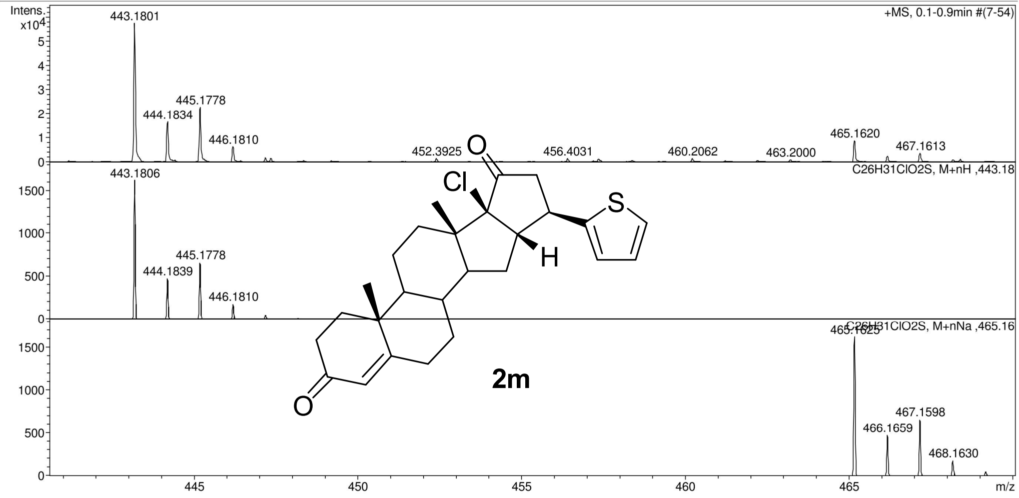

Bruker Compass DataAnalysis 4.0 printed: 13.11.2018 15:38:38 Page 1 of 1

VIII. References

[S1] Iselt, M.; Holtei, W.; Hilgard, P., "The tetrazolium dye assay for rapid in vitro assessment of cytotoxicity" *Arzneimittelforschung,* 1989, **39**, 747-749.

[S2] Volkova, Y.A.; Antonov, Y.S.; Komkov, A.V.; Scherbakov, A.M.; Shashkov, A.S.; Menchikov, L.G.; Chernoburova, E.I.; Zavarzin, I.V., "Access to steroidal pyridazines via modified thiohydrazides" *RSC Advances,* 2016, ***6***, 42863-42868.

[S3] Bruker. APEX-III. *Bruker AXS Inc.*, Madison, Wisconsin, USA, 2018.

[S4] Krause, L.; Herbst-Irmer, R.; Sheldrick, G. M.; Stalke, D. Comparison of silver and molybdenum microfocus X-ray sources for single-crystal structure determination. *J. Appl. Cryst.* 2015, **48**, 3–10. http://doi.org/10.1107/S1600576714022985

[S5] Sheldrick, G. M. SHELXT - Integrated space-group and crystal-structure determination. *ActaCryst.*2015, **A71**, 3-8. http://doi.org/10.1107/S2053273314026370

[S6] Sheldrick, G. M. Crystal structure refinement with SHELXL.*ActaCryst.*2015, C71, 3-8. http://doi.org/10.1107/S2053229614024218

[S7] Spek, A. L.PLATON SQUEEZE: a tool for the calculation of the disordered solvent contribution to the calculated structure factors. *ActaCryst.*, 2015, **C71**, 9-18, http://doi.org/10.1107/S2053229614024929

[S8] Spek, A. L. Structure validation in chemical crystallography.*ActaCryst.*, 2009, **D65**, 148-155, http://doi.org/10.1107/S090744490804362X

[S9] Flack, H.D. On enantiomorph-polarity estimation.*ActaCryst.*1983, **A39**, 876-881. http://doi.org/10.1107/S0108767383001762

[S10] Parsons, S.; Flack, H.D.; Wagner, T. Use of intensity quotients and differences inabsolute structure refinement. *ActaCryst.*2013, **B69**, 249-259. http://doi.org/10.1107/S2052519213010014

[S11] Gaussian 09, Revision D.01, M. J. Frisch, G. W. Trucks, H. B. Schlegel, G. E. Scuseria, M. A. Robb, J. R. Cheeseman, G. Scalmani, V. Barone, G. A. Petersson, H. Nakatsuji, X. Li, M. Caricato, A. Marenich, J. Bloino, B. G. Janesko, R. Gomperts, B. Mennucci, H. P. Hratchian, J. V. Ortiz, A. F. Izmaylov, J. L. Sonnenberg, D. Williams-Young, F. Ding, F. Lipparini, F. Egidi, J. Goings, B. Peng, A. Petrone, T. Henderson, D. Ranasinghe, V. G. Zakrzewski, J. Gao, N. Rega, G. Zheng, W. Liang, M. Hada, M. Ehara, K. Toyota, R. Fukuda, J. Hasegawa, M. Ishida, T. Nakajima, Y. Honda, O. Kitao, H. Nakai, T. Vreven, K. Throssell, J. A. Montgomery, Jr., J. E. Peralta, F. Ogliaro, M. Bearpark, J. J. Heyd, E. Brothers, K. N. Kudin, V. N. Staroverov, T. Keith, R. Kobayashi, J. Normand, K. Raghavachari, A. Rendell, J. C. Burant, S. S. Iyengar, J. Tomasi, M. Cossi, J. M. Millam, M. Klene, C. Adamo, R. Cammi, J. W. Ochterski, R. L. Martin, K. Morokuma, O. Farkas, J. B. Foresman, and D. J. Fox, Gaussian, Inc., Wallingford CT, 2016.

[S12] Chai, J.-D.; Head-Gordon M.; "Long-range corrected hybrid density functionals with damped atom-atom dispersion corrections" *Phys. Chem. Chem. Phys.*, 2008, **10**, 6615-6620.
